# Supplementary material for: Musculoskeletal defects associated with myosin heavy chain‐embryonic loss of function are mediated by the YAP signaling pathway
Source: EMBO Mol Med. 2023 Jul 26;15(9):e17187. doi: 10.15252/emmm.202217187 (PMC10493586; doi:10.15252/emmm.202217187)
Supplement: Supplementary file 1 — Appendix [file EMMM-15-e17187-s009.pdf]

Musculoskeletal defects associated with Myosin Heavy Chain-embryonic loss of function are mediated by the YAP signaling pathway.

Anushree Bharadwaj[1,*], Jaydeep Sharma[1,*], Jagriti Singh[1], Mahima Kumari[1], Tanushri Dargar[1,2], Bhargab Kalita[1,3] and Sam J. Mathew[1,#]

Appendix

Appendix Table S1: Antibodies used for immunofluorescence and western blots

Appendix Table S2: List of primers

Appendix Table S1: Antibodies used for immunofluorescence and western blots

Antibody	Type	Source	Product No.	Working Dilution
**Primary antibodies**				
Pax7	Mouse IgG1	Developmental Studies Hybridoma Bank	PAX7	1:10 (IF, WB)
Laminin	Rabbit polyclonal	Sigma	L9393	1:400 (IF)
MyHC slow	Mouse IgG1	Sigma	M8421 (NOQ7.5.4D)	1:1000 (IF, WB)
MyHC-IIa	Mouse IgG1	Developmental Studies Hybridoma Bank	SC-71	1:10 (IF, WB)
MyHC-IIb	Mouse IgM	Developmental Studies Hybridoma Bank	BFF3	1:10 (IF, WB)
MyHC-IIx	Mouse IgM	Developmental Studies Hybridoma Bank	6H1	1:50 (IF, WB)
Talin 1	Rabbit IgG1	Cloud-Clone Corp	RPA278Mu01	1:1000 (WB)
YAP (D8H1X)	Rabbit IgG	Cell Signaling Technology	14074	1:1000 (WB)
P-YAP (S109)	Rabbit IgG	Cell Signaling Technology	46931	1:1000 (WB)
P-YAP (S127)	Rabbit IgG	Cell Signaling Technology	13008	1:1000 (WB)
CTGF (D8Z8U)	Rabbit IgG	Cell Signaling Technology	86641	1:1000 (WB)
CYR61 (D4H5D)	Rabbit IgG	Cell Signaling Technology	14479	1:1000 (WB)
NEDD4	Rabbit IgG	Cell Signaling Technology	2740	1:1000 (WB)
Anti-Histone H3	Rabbit IgG	Abcam	Ab1791	1:2000 (WB)
GAPDH	Rabbit IgG	Cloud-Clone Corp.	PAB932Mu01	1:10,000 (WB)
Filamin B	Rabbit IgG	Cell Signaling Technology	12979S	1:1000 (WB)
Pan-TEAD (D3F7L)	Rabbit IgG	Cell Signaling Technology	13295	1:1000 (WB)
TAZ (D316D)	Rabbit IgG	Cell Signaling Technology	70148	1:1000 (WB)
Caspase3	Rabbit IgG	Cloud-Clone Corp.	PAA626Mu01	1:1000 (WB)
MyoD	Rabbit IgG	Santa Cruz	Sc-377460	1:400 (WB)

**Secondary antibodies**				
Cy2/Cy3 conjugated Goat anti-rabbit	Goat	Jackson ImmunoResearch Laboratories	111-225-144/ 111-165-144	1:500 (IF)
Biotin conjugated Goat anti-mouse	Goat	Jackson ImmunoResearch Laboratories	115-065-020	1:500 (IF)
Biotin conjugated Goat anti-rabbit	Goat	Jackson ImmunoResearch Laboratories	111-065-144	1:500 (IF)
Cy2 conjugated streptavidin	-	Jackson ImmunoResearch Laboratories	016-220-084	1:500 (IF)
Cy3 conjugated streptavidin	-	Jackson ImmunoResearch Laboratories	016-160-084	1:500 (IF)
Peroxidase-AffiniPure Goat anti-rabbit	Goat	Jackson ImmunoResearch Laboratories	111-035-144	1:20,000 (WB)
Peroxidase-AffiniPure Goat anti-mouse	Goat	Jackson ImmunoResearch Laboratories	111-035-003	1:20,000 (WB)

Appendix Table S2: List of primers

Gene	Direction	Primer sequence 5’ to 3’	Product size (bp) using cDNA template
CTGF	Forward<br>Reverse	TGGAAGACACATTTGGC<br>GTCATTGGTAACTCGGGT	122
CYR61	Forward<br>Reverse	GAGGCTTCCTGTCTTTGGCAC<br>ACTCTGGGTTGTCATTGGTAA	155
AMOT	Forward<br>Reverse	GATGTGCAACCCAGATAAGCC<br>TCTCTGCATCAGGCTCTTGC	101
MOB1A	Forward<br>Reverse	TCGGAAGTGGCAATCTGAGG<br>CAGCTTGCCTCAGTGCAGAA	145
MOB1B	Forward<br>Reverse	CTACGGATGGCTGTCATGCT<br>GACATCACCGGACAGCTCTC	143
