# Supplementary material for: Musculoskeletal defects associated with myosin heavy chain‐embryonic loss of function are mediated by the YAP signaling pathway
Source: EMBO Mol Med. 2023 Jul 26;15(9):e17187. doi: 10.15252/emmm.202217187 (PMC10493586; doi:10.15252/emmm.202217187)
Supplement: Supplementary file 2 — Expanded View Figures PDF [file EMMM-15-e17187-s003.pdf]

Expanded View Figures

Figure EV1. Loss of MyHC-embryonic leads to alterations in myofiber number, size and function.

A, B Quantification of the number of myofibers normalized to cross-sectional area (CSA) (mm^2) (n = 4 mice per genotype) through the gastrocnemius muscle of *Myh3*$^{+/+}$ and *Myh3*$^{\Delta/\Delta}$ mice at 8–10 weeks (A) and 6 months (B) of age.

C, D Quantification of the number of myofibers normalized to cross-sectional area (CSA) (mm^2) (n = 4 mice per genotype) through the soleus muscle of *Myh3*$^{+/+}$ and *Myh3*$^{\Delta/\Delta}$ mice at 8–10 weeks (C) and 6 months (D) of age.

E–I Quantification of the number of myofibers grouped according to area through the TA muscle (n = 4 mice per genotype) at 8–10 weeks of age (E), through the gastrocnemius muscle (n = 3 mice per genotype) at 8–10 weeks (F) and 6 months (G) of age, and through the soleus muscle (n = 3 mice per genotype) at 8–10 weeks (H) and 6 months (I) of age of *Myh3*$^{+/+}$ and *Myh3*$^{\Delta/\Delta}$ mice.

J–K Bright-field images of skeletal preparations of representative *Myh3*$^{+/+}$ and *Myh3*$^{\Delta/\Delta}$ 4-week-old mice showing the cervical region, stained with Alcian blue and Alizarin red.

L, M Quantification of the percentage of cervical (L) and lumbar (M) vertebral fusion in *Myh3*$^{+/+}$ and *Myh3*$^{\Delta/\Delta}$ mice.

N Graph depicting results from treadmill exhaustion test measured as distance traveled (meters) by 4-month-old *Myh3*$^{+/+}$ and *Myh3*$^{\Delta/\Delta}$ mice.

O Plots for myosin ATPase activity from hind limb muscle of embryonic day (E) 16.5 *Myh3*$^{+/+}$ and *Myh3*$^{\Delta/\Delta}$ embryos (n = 3 mice per genotype).

Data information: Data are presented as mean ± SEM. Student's t-test was performed, with $P \leq 0.05$ considered significant.

© 2023 The Authors

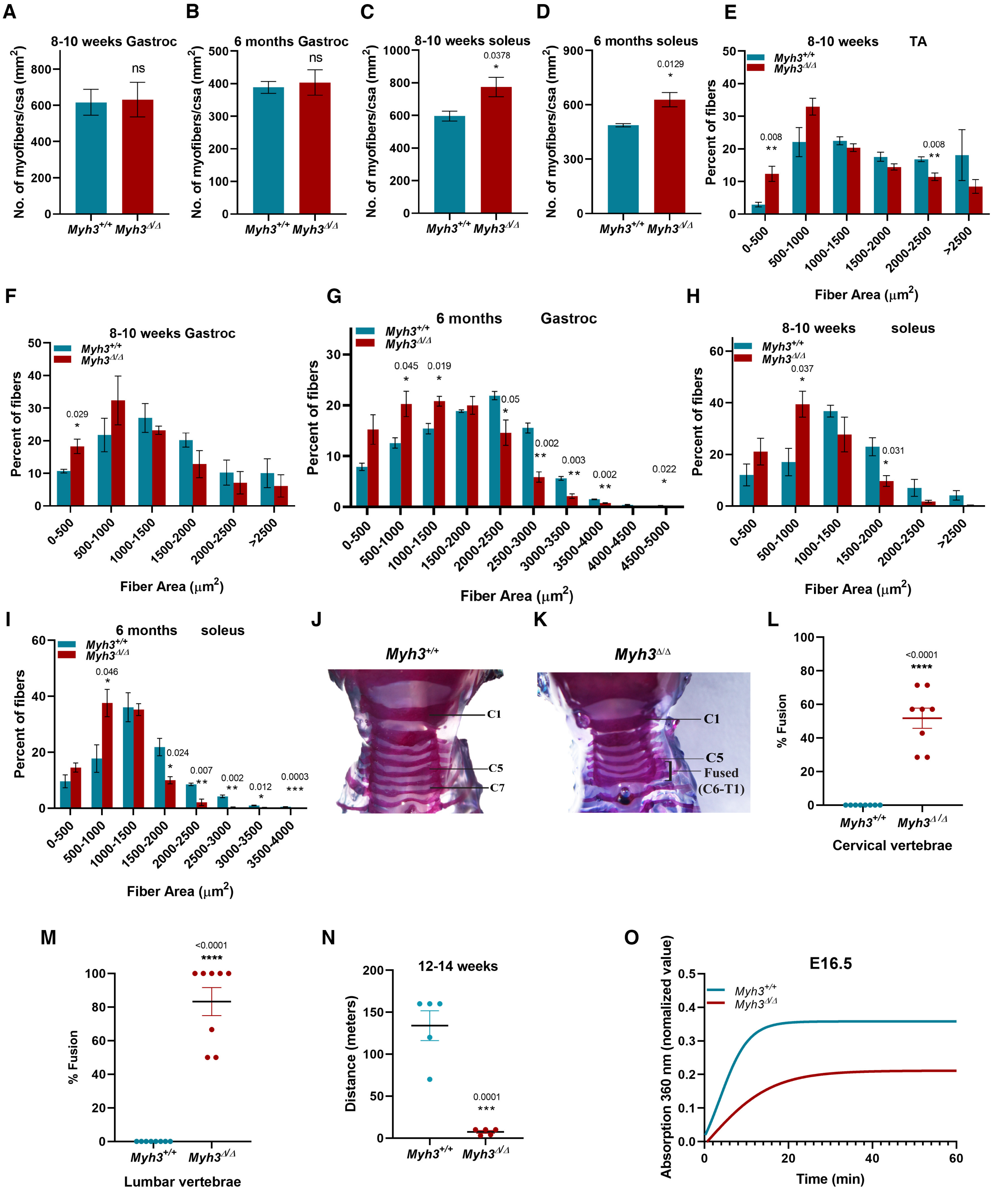
A
8-10 weeks Gastroc
No. of myofibers/csa (mm²)
ns
Myh3+/+ Myh3Δ/Δ
B
6 months Gastroc
ns
C
8-10 weeks soleus
0.0378
D
6 months soleus
0.0129
E
8-10 weeks TA
Percent of fibers
0.008
Fiber Area (μm²)
F
8-10 weeks Gastroc
0.029
G
6 months Gastroc
0.045
0.019
0.05
0.002
0.003
0.002
0.022
H
8-10 weeks soleus
0.037
0.031
I
6 months soleus
0.046
0.024
0.007
0.002
0.012
0.0003
J
Myh3+/+
C1
C5
C7
K
Myh3Δ/Δ
C1
C5
Fused
(C6-T1)
L
% Fusion
<0.0001
Cervical vertebrae
M
<0.0001
Lumbar vertebrae
N
12-14 weeks
Distance (meters)
0.0001
O
E16.5
Absorption 360 nm (normalized value)
Time (min)

Figure EV1.

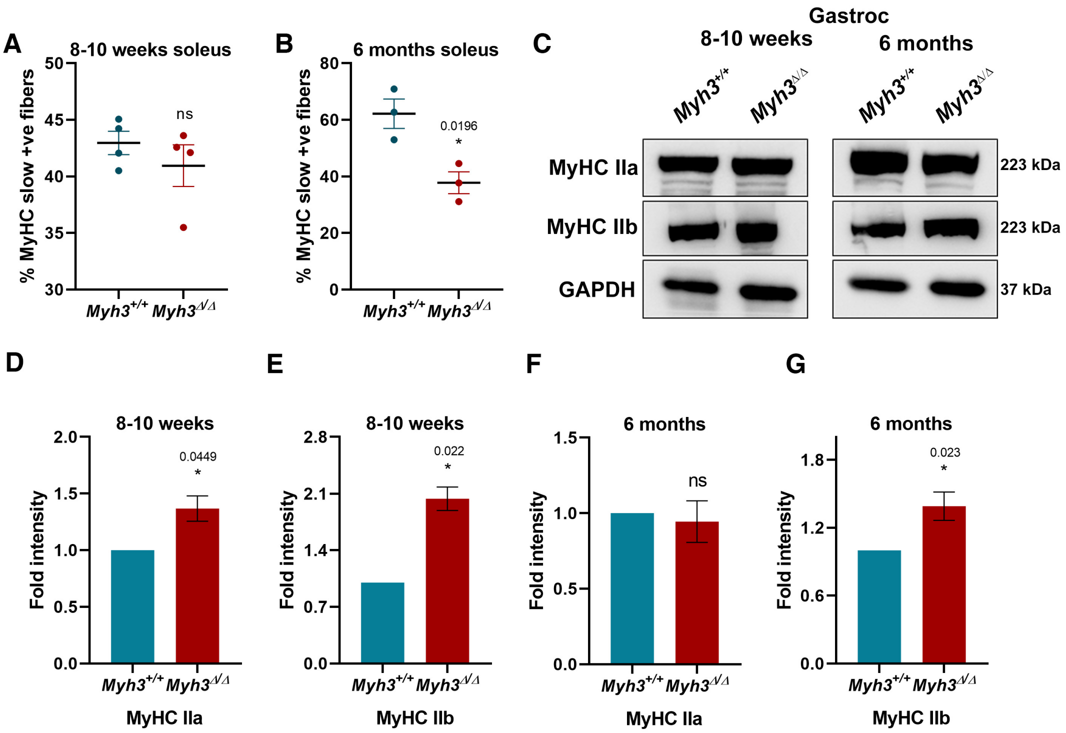

Figure EV2. Loss of MyHC-embryonic function leads to muscle fiber type changes.

A, B Quantification of the percentage of MyHC-slow-positive fibers normalized to total number of fibers through the soleus muscle of *Myh3*$^{+/+}$ and *Myh3*$^{\Delta/\Delta}$ mice at 8–10 weeks and 6 months of age, respectively.

C–G Representative western blots for MyHC-IIa, -IIb, and GAPDH using protein lysates from the gastrocnemius muscle of *Myh3*$^{+/+}$ and *Myh3*$^{\Delta/\Delta}$ mice at 8–10 weeks and 6 months of age (C) and their densitometric quantification (D–G) (n = 4 mice per genotype).

Data information: Data are presented as mean ± SEM. Student's t-test was performed, with $P \leq 0.05$ considered significant.

© 2023 The Authors

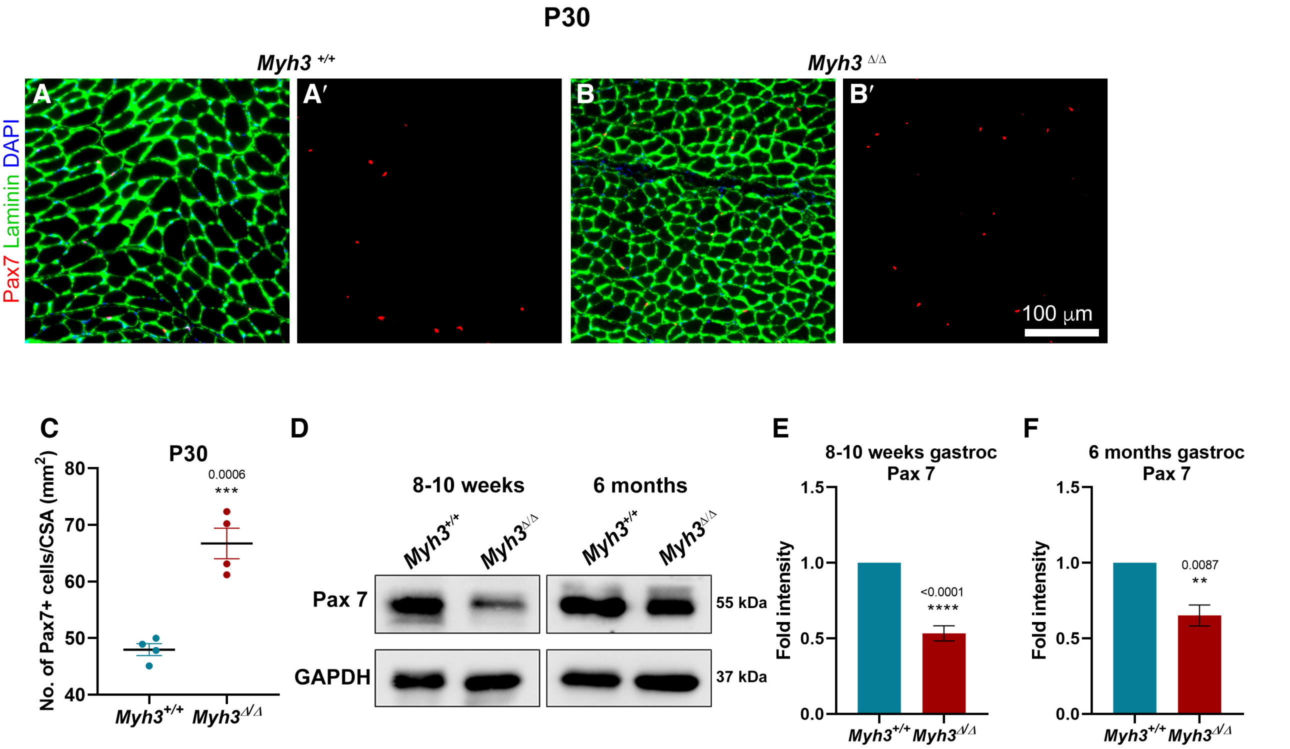

Figure EV3. Loss of MyHC-embryonic function leads to alterations in satellite cell numbers.

A–C Representative fluorescent micrographs of transverse sections through the TA muscle of *Myh3*$^{+/+}$ and *Myh3*$^{\Delta/\Delta}$ mice at postnatal Day 30 stained with Pax7 (red), Laminin (green) and DAPI (blue) (A–B′), and quantification of the number of Pax7-positive cells per unit area (mm^2) (C).

D–F Representative western blots for Pax7 and GAPDH using protein lysates from the gastrocnemius muscle of *Myh3*$^{+/+}$ and *Myh3*$^{\Delta/\Delta}$ mice at 8–10 weeks and 6 months of age (D) and their densitometric quantification (E, F) (n = 4 mice per genotype in E and n = 5 mice per genotype in F).

Data information: Data are presented as mean ± SEM. Student's *t*-test was performed, with $P \leq 0.05$ considered significant. Scale bar: 100 µm (B′).

© 2023 The Authors

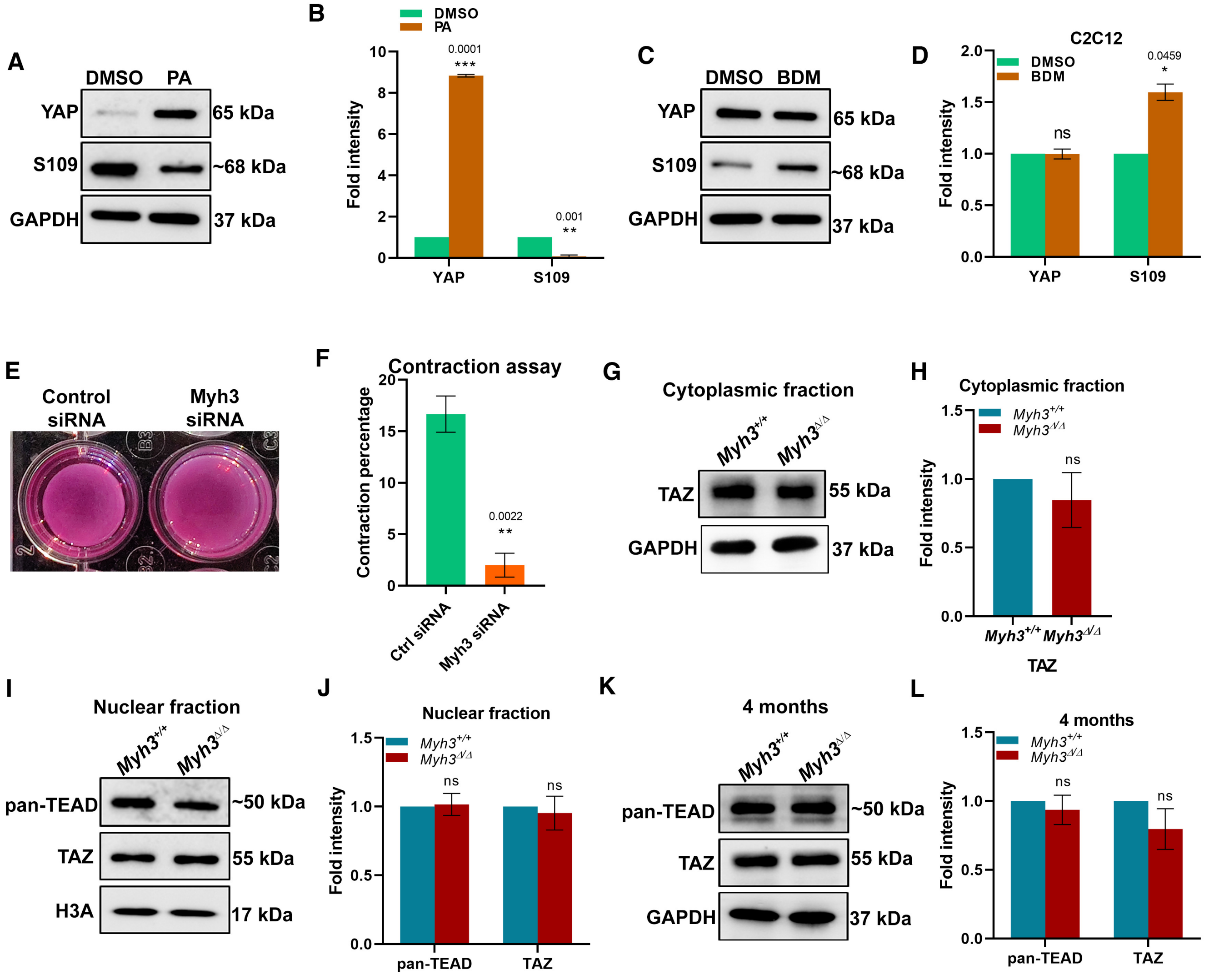

Figure EV4. Myosin inhibitors modulate YAP expression and activation.

A, B Representative western blots for total YAP, phospho-YAP (S109), and GAPDH using protein lysates from C2C12 cells treated with DMSO or para-aminoblebbistatin (A) and their densitometric quantification (B) (n = 3 biological replicates).

C, D Representative western blots for total YAP, phospho-YAP (S109), and GAPDH using protein lysates from C2C12 cells treated with DMSO or 2,3-Butanedione monoxime (BDM) (C) and their densitometric quantification (D) (n = 4 biological replicates).

E, F Representative micrograph showing collagen matrix following culture of control or *Myh3* siRNA-treated C2C12 cells (E) and quantification of the percentage of matrix contraction (F). (n = 3 biological replicates).

G, H Representative western blots for TAZ and GAPDH in the cytoplasmic fraction (G), isolated from the TA muscle of 4-month-old $Myh3^{+/+}$ and $Myh3^{\Delta/\Delta}$ mice (E) and densitometric quantification (H) (n = 5 mice per genotype).

I–L Representative western blots for pan-TEAD, TAZ, and histone H3A in the nuclear fraction (I) and pan-TEAD, TAZ, and GAPDH in the total lysate (K) isolated from the TA muscle of 4-month-old $Myh3^{+/+}$ and $Myh3^{\Delta/\Delta}$ mice and their respective densitometric quantification (J, L) (n = 4 mice per genotype in J and n = 5 mice per genotype in L).

 © 2023 The Authors

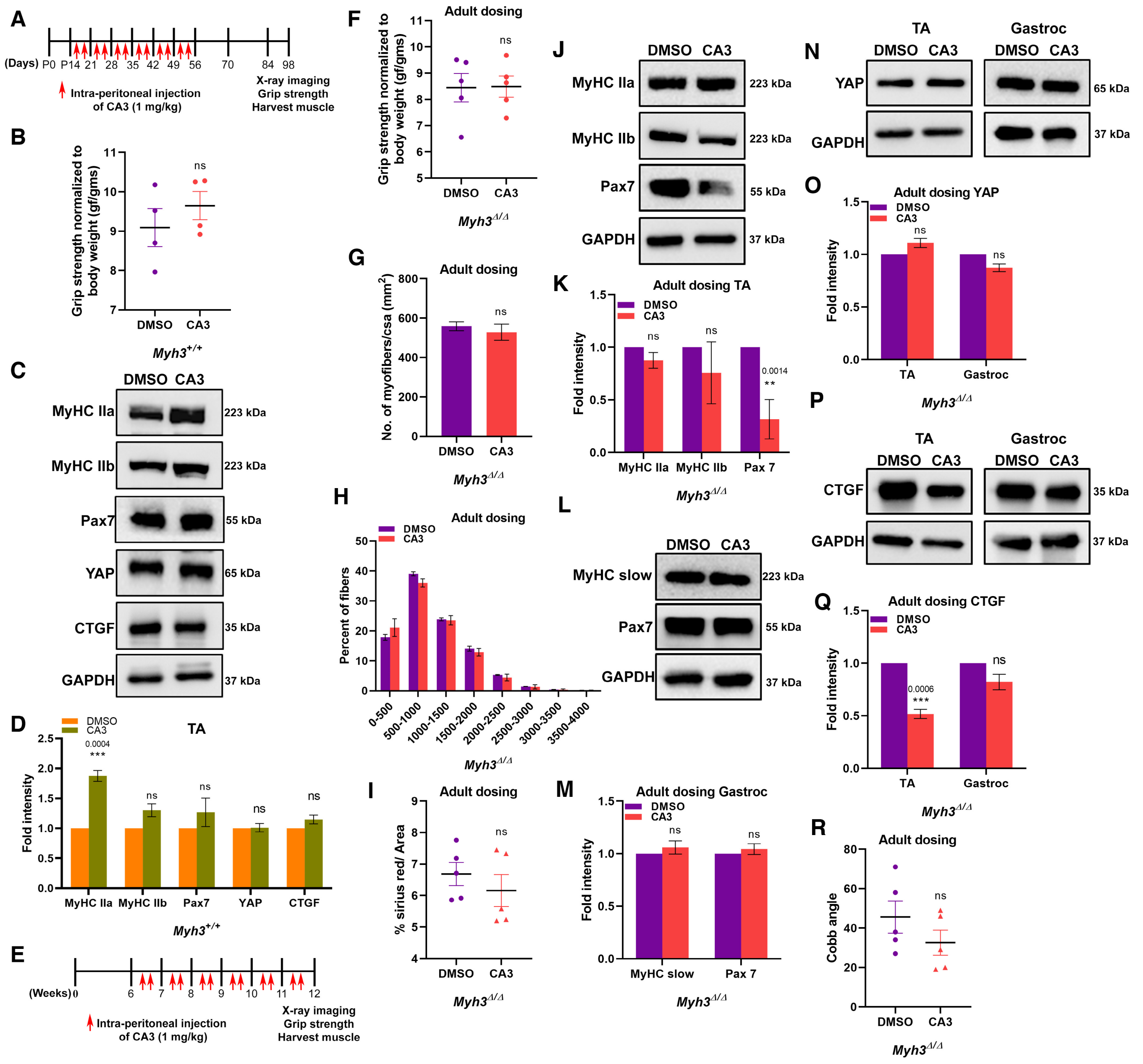
A
(Days) P0 P14 21 28 35 42 49 56 70 84 98
Intra-peritoneal injection of CA3 (1 mg/kg)
X-ray imaging
Grip strength
Harvest muscle
B
Grip strength normalized to body weight (gf/gms)
ns
DMSO CA3
Myh3+/+
C
DMSO CA3
MyHC IIa 223 kDa
MyHC IIb 223 kDa
Pax7 55 kDa
YAP 65 kDa
CTGF 35 kDa
GAPDH 37 kDa
D
DMSO
CA3
TA
Fold intensity
0.0004
***
ns
MyHC IIa MyHC IIb Pax7 YAP CTGF
Myh3+/+
E
(Weeks) 0 6 7 8 9 10 11 12
Intra-peritoneal injection of CA3 (1 mg/kg)
X-ray imaging
Grip strength
Harvest muscle
F
Adult dosing
Grip strength normalized to body weight (gf/gms)
ns
DMSO CA3
Myh3Δ/Δ
G
Adult dosing
No. of myofibers/csa (mm2)
ns
DMSO CA3
Myh3Δ/Δ
H
Adult dosing
DMSO
CA3
Percent of fibers
0-500 500-1000 1000-1500 1500-2000 2000-2500 2500-3000 3000-3500 3500-4000
Myh3Δ/Δ
I
Adult dosing
% sirius red/ Area
ns
DMSO CA3
Myh3Δ/Δ
J
DMSO CA3
MyHC IIa 223 kDa
MyHC IIb 223 kDa
Pax7 55 kDa
GAPDH 37 kDa
K
Adult dosing TA
DMSO
CA3
Fold intensity
ns
0.0014
**
MyHC IIa MyHC IIb Pax 7
Myh3Δ/Δ
L
DMSO CA3
MyHC slow 223 kDa
Pax7 55 kDa
GAPDH 37 kDa
M
Adult dosing Gastroc
DMSO
CA3
Fold intensity
ns
MyHC slow Pax 7
Myh3Δ/Δ
N
TA Gastroc
DMSO CA3 DMSO CA3
YAP 65 kDa
GAPDH 37 kDa
O
Adult dosing YAP
DMSO
CA3
Fold intensity
ns
TA Gastroc
Myh3Δ/Δ
P
TA Gastroc
DMSO CA3 DMSO CA3
CTGF 35 kDa
GAPDH 37 kDa
Q
Adult dosing CTGF
DMSO
CA3
Fold intensity
0.0006
***
ns
TA Gastroc
Myh3Δ/Δ
R
Adult dosing
Cobb angle
ns
DMSO CA3
Myh3Δ/Δ

Figure EV5.

◀ **Figure EV5. Administration of CA3 in adults fails to rescue most abnormalities seen in *Myh3*$^{\Delta/\Delta}$ mice.**

A Schematic depicting CA3 dosing strategy starting from postnatal day 15.

B Quantification of grip strength normalized to body weight of *Myh3*$^{+/+}$ mice at 12–14 weeks of age, treated with DMSO (vehicle) or CA3.

C, D Representative western blots for MyHC-IIa, -IIb, Pax7, YAP, CTGF, and GAPDH using protein lysates from the TA muscle of *Myh3*$^{+/+}$ mice at 12–14 weeks of age, treated with DMSO or CA3 (C) and their densitometric quantification (D) (n = 4 mice per genotype).

E Schematic depicting CA3 dosing strategy starting from 6 weeks after birth (adult dosing).

F Quantification of grip strength normalized to body weight of *Myh3*$^{\Delta/\Delta}$ mice at 12–14 weeks of age, treated with DMSO (vehicle) or CA3 starting from 6 weeks after birth.

G Quantification of the number of myofibers normalized to cross-sectional area (CSA) (mm^2) through the TA muscle of *Myh3*$^{\Delta/\Delta}$ mice at 12–14 weeks of age, treated with DMSO or CA3 starting from 6 weeks after birth (n = 5 mice per genotype).

H Quantification of the number of myofibers grouped according to myofiber area through the TA muscle of *Myh3*$^{\Delta/\Delta}$ mice at 12–14 weeks of age, treated with DMSO or CA3 starting from 6 weeks after birth (n = 3 mice per genotype).

I Quantification of the percentage of Sirius red-positive area as a fraction of total area in transverse sections through the TA muscle of *Myh3*$^{\Delta/\Delta}$ mice at 12–14 weeks of age, treated with DMSO or CA3 starting from 6 weeks after birth.

J, K Representative western blots for MyHC-IIa, -IIb, Pax7, and GAPDH using protein lysates from the TA muscle of *Myh3*$^{\Delta/\Delta}$ mice at 12–14 weeks of age, treated with DMSO or CA3 starting from 6 weeks after birth (J) and their densitometric quantification (K) (n = 5 mice per genotype).

L, M Representative western blots for MyHC-slow, Pax7, and GAPDH using protein lysates from the gastrocnemius muscle of *Myh3*$^{\Delta/\Delta}$ mice at 12–14 weeks of age, treated with DMSO or CA3 starting from 6 weeks after birth (L) and their densitometric quantification (M) (n = 5 mice per genotype).

N, O Representative western blots for total YAP and GAPDH in the TA and the gastrocnemius (N) protein lysates of *Myh3*$^{\Delta/\Delta}$ mice at 12–14 weeks of age, treated with DMSO or CA3 starting from 6 weeks after birth and their densitometric quantification (O) (n = 6 mice per genotype).

P, Q Representative western blots for CTGF and GAPDH in the TA and the gastrocnemius (P) protein lysates of *Myh3*$^{\Delta/\Delta}$ mice at 12–14 weeks of age, treated with DMSO or CA3 starting from 6 weeks after birth and their densitometric quantification (Q) (n = 4 mice per genotype).

R Quantification of the Cobb angle of *Myh3*$^{\Delta/\Delta}$ mice at 12–14 weeks of age, treated with DMSO or CA3 starting from 6 weeks after birth.

Data information: Data are presented as mean ± SEM. Student's t-test was performed, with $P \leq 0.05$ considered significant.

© 2023 The Authors
