# Supplementary material for: Musculoskeletal defects associated with myosin heavy chain‐embryonic loss of function are mediated by the YAP signaling pathway
Source: EMBO Mol Med. 2023 Jul 26;15(9):e17187. doi: 10.15252/emmm.202217187 (PMC10493586; doi:10.15252/emmm.202217187)
Supplement: Supplementary file 3 — PDF+ [file EMMM-15-e17187-s008.pdf]

SOURCE DATA TRANSPARENT PROCESS OPEN ACCESS

EMBO Molecular Medicine

Article

Musculoskeletal defects associated with myosin heavy chain-embryonic loss of function are mediated by the YAP signaling pathway

Anushree Bharadwaj[†], Jaydeep Sharma[†], Jagriti Singh, Mahima Kumari, Tanushri Dargar[‡], Bhargab Kalita[§] & Sam J Mathew*

Abstract

Mutations in *MYH3*, the gene encoding the developmental myosin heavy chain-embryonic (MyHC-embryonic) skeletal muscle-specific contractile protein, cause several congenital contracture syndromes. Among these, recessive loss-of-function *MYH3* mutations lead to spondylocarpotarsal synostosis (SCTS), characterized by vertebral fusions and scoliosis. We find that *Myh3* germline knockout adult mice display SCTS phenotypes such as scoliosis and vertebral fusion, in addition to reduced body weight, muscle weight, myofiber size, and grip strength. *Myh3* knockout mice also exhibit changes in muscle fiber type, altered satellite cell numbers and increased muscle fibrosis. A mass spectrometric analysis of embryonic skeletal muscle from *Myh3* knockouts identified integrin signaling and cytoskeletal regulation as the most affected pathways. These pathways are closely connected to the mechanosensing Yes-associated protein (YAP) transcriptional regulator, which we found to be significantly activated in the skeletal muscle of *Myh3* knockout mice. To test whether increased YAP signaling might underlie the musculoskeletal defects in *Myh3* knockout mice, we treated these mice with CA3, a small molecule inhibitor of YAP signaling. This led to increased muscle fiber size, rescue of most muscle fiber type alterations, normalization of the satellite cell marker Pax7 levels, increased grip strength, reduced fibrosis, and decline in scoliosis in *Myh3* knockout mice. Thus, increased YAP activation underlies the musculoskeletal defects seen in *Myh3* knockout mice, indicating its significance as a key pathway to target in SCTS and other *MYH3*-related congenital syndromes.

Keywords Homeostasis; Mice; Myosin heavy chain-embryonic; Skeletal muscle; YAP

Subject Categories Genetics, Gene Therapy & Genetic Disease; Musculoskeletal System

DOI 10.15252/emmm.202217187 | Received 16 November 2022 | Revised 13 July 2023 | Accepted 17 July 2023 | Published online 26 July 2023

EMBO Mol Med (2023) 15: e17187

Introduction

The mammalian skeletal muscle arises from the differentiation and fusion of progenitors or stem cells to give rise to contractile myofibers. Sarcomeres present in the myofibers are the units of muscle contraction and are made up of thin and thick filaments. Myosins are the major contractile proteins of the thick filaments; each myosin molecule is made up of a pair of heavy chains, and a pair each of essential and regulatory light chains. Myosin heavy chains (MyHCs) interact with actin in the thin filaments and are ATPases that generate the mechanical force of contraction, facilitating skeletal muscle function. Multiple MyHC isoforms with varying contractile velocities are present in the mammalian skeletal muscle, of which two are expressed only during embryonic development and transiently during regeneration following skeletal muscle injury or disease in adults. These are MyHC-embryonic and MyHC-perinatal, broadly referred to as developmental MyHCs. The other MyHC isoforms are MyHC-slow expressed both in embryonic and adult stages, and MyHC-IIa, -IIx, and -IIb expressed only during adult stages. Previously, we showed that MyHC-embryonic function is essential for proper myogenic differentiation during development, using targeted mouse models (Agarwal *et al*, 2020).

Mutations in *MYH3*, the gene encoding MyHC-embryonic, have been reported to result in a range of musculoskeletal disorders primarily of the distal arthrogryposis (DA) type. Dominant missense mutations in *MYH3* were shown to cause the DA type 1, DA type 2A Freeman–Sheldon syndrome, and the DA type 2B Sheldon–Hall syndrome (Toydemir *et al*, 2006; Beck *et al*, 2013, 2014). More recently, *MYH3* mutations have also been reported to lead to multiple pterygium syndrome (DA type 8) and spondylocarpotarsal synostosis (SCTS; Carapito *et al*, 2016; Chong *et al*, 2015). While all of these congenital abnormalities have some shared phenotypes such as contractures and scoliosis of varying severity, they also have unique characteristics, possibly related to the type of mutation, domain affected, and functions that may be altered. Several of these abnormalities result from missense mutations that might alter the

Developmental Genetics Laboratory, Regional Centre for Biotechnology (RCB), NCR Biotech Science Cluster, Faridabad, India
*Corresponding author. Tel: +91 129-284-8822; E-mail: sjmathew@rcb.res.in
[†]These authors contributed equally to this work
[‡]Present address: Faculte de Medicine, Institut NeuroMyoGene, Lyon, France
[§]Present address: Department of Pathology and Perlmutter Cancer Center, New York University School of Medicine, New York, NY, USA

© 2023 The Authors. Published under the terms of the CC BY 4.0 license

contractile properties of MyHC-embryonic, leading to dominant effects (Chong *et al*, 2015; Schiaffino *et al*, 2015). Interestingly, recent studies have started to identify recessive *MYH3* mutations in the compound heterozygous condition, which lead to SCTS (Cameron-Christie *et al*, 2018). A recent case study reports that homozygous or compound heterozygous *MYH3* mutations resulting in near-complete to total loss of function of MyHC-embryonic in three fetuses led to SCTS-like phenotypes with fetal lethality (Kamien *et al*, 2022). This suggests that *MYH3*-associated SCTS most likely results from the loss of function of MyHC-embryonic rather than dominant mutations that alter its contractile properties.

There are few reports on the muscle pathology of patients with *MYH3* mutation-associated musculoskeletal diseases. One study reported an increase in MyHC-perinatal-positive myofibers at 15 months, variability in fiber size, uneven distribution of MyHC-slow myofibers, and increase in muscle connective tissue in Freeman–Sheldon and Sheldon–Hall syndrome patients (Tajsharghi *et al*, 2008). Animal models for Freeman–Sheldon and Sheldon–Hall syndrome mutations have been generated in *Drosophila*, which exhibited severe muscle defects (Das *et al*, 2019; Rao *et al*, 2019). A zebrafish model for the Freeman–Sheldon syndrome mutation (R673H) incorporated in the *slow myosin heavy chain 1* (*smyhc1*) gene showed musculoskeletal abnormalities such as scoliosis, vertebral fusion, and shortened muscle, which were rescued by para-aminoblebbistatin, an inhibitor of myosin ATPase activity (Whittle *et al*, 2020). No mammalian models for the *Myh3*-associated mutations have been reported yet, although Dr. Leslie Leinwand's group generated transgenic mice with the *Myh3 R672H* mutation, where no obvious phenotypes were seen and the transgene stopped expressing after a few generations (personal communication from Dr. Leslie Leinwand).

The Yes-associated protein (YAP) is a transcriptional regulator of crucial cellular properties such as proliferation, size, differentiation, and apoptosis. YAP functions downstream of the Hippo signaling pathway, where serine/threonine phosphorylation by kinases and associated proteins such as MST, LATS, and MOB1 regulate YAP stability, localization, and activity (Zhao *et al*, 2007, 2010). YAP is also known to sense and respond to mechanical signals in the cellular microenvironment (Dupont *et al*, 2011). The integrin family of transmembrane receptors are key mechanotransduction mediators that link the actin cytoskeleton inside the cell to the extracellular matrix (Sun *et al*, 2016). Since both integrins and YAP are involved in mechanotransduction, it is not surprising that integrins are known to mediate signals leading to YAP pathway activation (Tang *et al*, 2013; Elbediwy *et al*, 2016; Yamashiro *et al*, 2020). YAP signaling is central to skeletal muscle function, where it is required for maintaining satellite cell quiescence and proliferation (Judson *et al*, 2012; Zhang *et al*, 2019), muscle protein synthesis, fiber size, and homeostasis (Judson *et al*, 2013; Watt *et al*, 2015).

Here, we characterize the musculoskeletal defects in *Myh3* germline knockout mice, a model for SCTS, at different stages of adult life. We find that loss of MyHC-embryonic leads to reduction in body weight, muscle weight, myofiber size, and grip strength in adult mice. *Myh3* germline knockout adult mice also exhibit scoliosis and vertebral fusion, increase in MyHC-IIb and -IIa fiber types and decrease in MyHC-slow fiber type, muscle-type, and age-dependent alterations in satellite cell numbers and increased muscle fibrosis. A mass spectrometric analysis suggested that proteins related to integrin signaling and cytoskeletal regulation are maximally dysregulated in *Myh3* knockout embryos. YAP signaling was tested as a potential downstream mechanotransduction pathway that might be altered upon loss of MyHC-embryonic which was indeed found to be the case. *Myh3* knockout mice exhibited increase in total YAP, decrease in phospho-YAP, and elevated expression of YAP target genes, indicating that increased activation of YAP signaling might underlie the musculoskeletal defects seen upon loss of MyHC-embryonic function. This was tested by inhibiting YAP signaling by treatment with the small molecule CA3, which led to increase in muscle fiber size, decrease in MyHC-IIa fibers, increase in MyHC-slow fibers, normalization of Pax7 levels, increased grip strength, decline in fibrosis, and reduction in scoliosis in adult *Myh3* knockout mice. Thus, our results indicate that augmented YAP signaling underlies many of the musculoskeletal defects seen upon loss of MyHC-embryonic function and might be an important potential pathway to target in patients with SCTS and other *MYH3*-related congenital syndromes.

Results

Loss of MyHC-embryonic function leads to adult musculoskeletal defects

Previously, we characterized the essential functions of MyHC-embryonic (encoded by the *Myh3* gene) in skeletal muscle differentiation during embryonic and early postnatal stages of development (Agarwal *et al*, 2020). As previously described, we observed 11% lethality of *Myh3* germline knockout ($Myh3^{\Delta/\Delta}$) embryos during developmental stages; the remaining 14% completed embryonic development and were born as pups (Agarwal *et al*, 2020). Although these surviving *Myh3* germline knockout mice exhibited severe muscle defects affecting myofiber number, area, fiber type, and satellite cell numbers in embryonic and early postnatal stages, this did not result in increased postnatal lethality compared with controls (Agarwal *et al*, 2020). On the other hand, additional defects such as scoliosis became apparent in $Myh3^{\Delta/\Delta}$ mice at adult stages (Agarwal *et al*, 2020), indicating that the embryonic loss of MyHC-embryonic might have effects at later stages or that it might have essential functions in adult life, which we wished to characterize.

$Myh3^{\Delta/\Delta}$ mice exhibited significantly reduced total adult body weight consistently, as shown in male mice at 8–10 weeks and 6 months of age (Fig 1A and B). Muscle weights for the tibialis anterior (TA) and gastrocnemius, taken as representative muscles of differing fiber type composition, also showed a significant reduction of > 25% at 8–10 weeks and 6 months, indicating that reduced muscle weight contributed to the reduction in total body weight in $Myh3^{\Delta/\Delta}$ male mice (Fig 1C–F). Interestingly, the number of myofibers per unit area of TA showed no significant difference at 8–10 weeks in $Myh3^{\Delta/\Delta}$ mice, whereas a significant increase was seen at 6 months (Fig 1G–J). The number of myofibers per unit area showed no significant difference at 8–10 weeks and 6 months in the gastrocnemius of $Myh3^{\Delta/\Delta}$ mice (Fig EV1A and B), whereas a significant increase was seen in the soleus muscle of $Myh3^{\Delta/\Delta}$ mice at both time points (Fig EV1C and D). These results suggest that some of the consequences of loss of MyHC-embryonic are dynamic and muscle specific. To characterize this further, myofibers were grouped

© 2023 The Authors

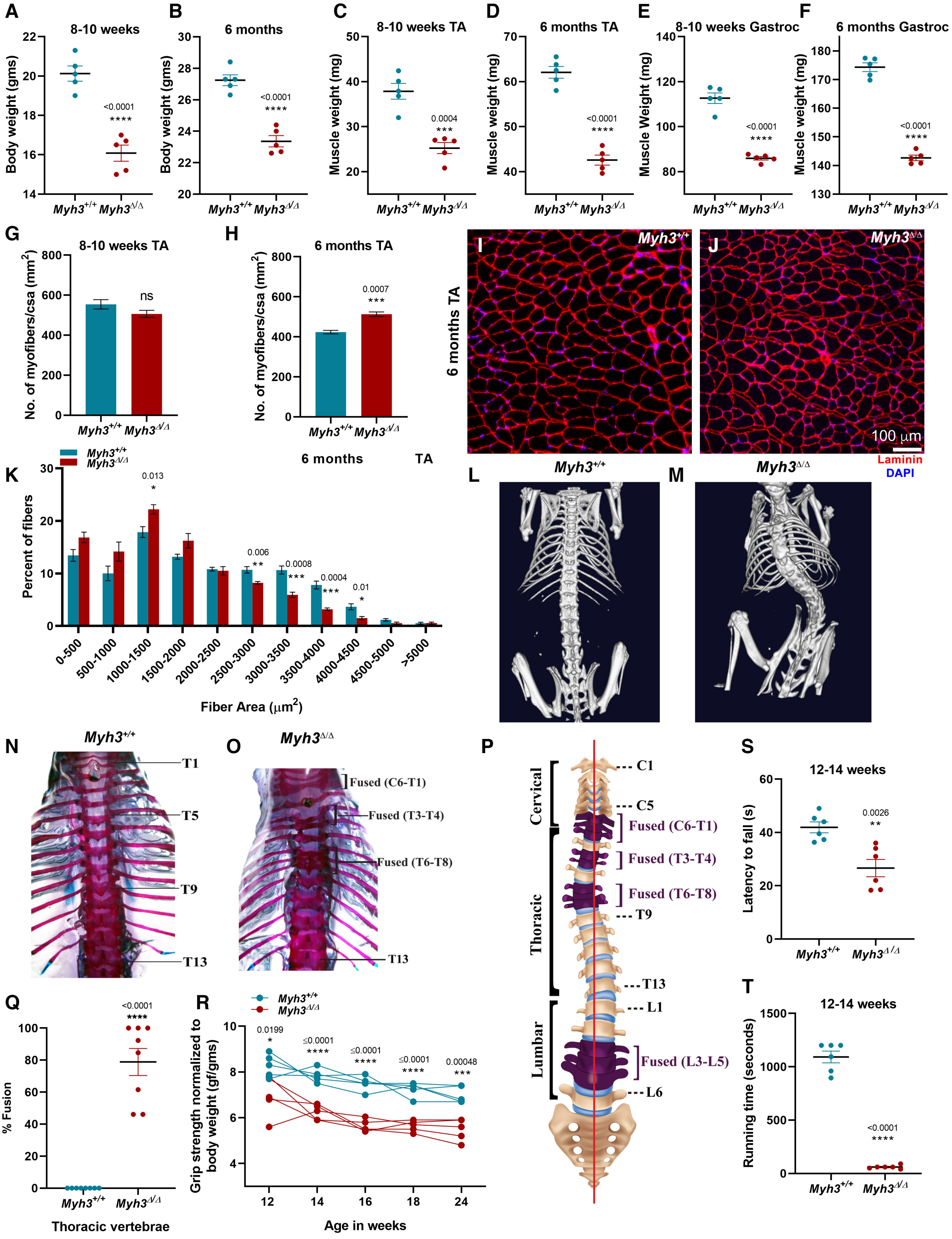

A 8-10 weeks
Body weight (gms)
<0.0001 ****
B 6 months
Body weight (gms)
<0.0001 ****
C 8-10 weeks TA
Muscle weight (mg)
0.0004 ***
D 6 months TA
Muscle weight (mg)
<0.0001 ****
E 8-10 weeks Gastroc
Muscle Weight (mg)
<0.0001 ****
F 6 months Gastroc
Muscle weight (mg)
<0.0001 ****
Myh3+/+ Myh3Δ/Δ
G 8-10 weeks TA
No. of myofibers/csa (mm2)
ns
H 6 months TA
No. of myofibers/csa (mm2)
0.0007 ***
6 months TA
I Myh3+/+
J Myh3Δ/Δ
100 μm
Laminin
DAPI
6 months TA
K
Percent of fibers
0.013 *
0.006 **
0.0008 ***
0.0004 ***
0.01 *
0-500
500-1000
1000-1500
1500-2000
2000-2500
2500-3000
3000-3500
3500-4000
4000-4500
4500-5000
>5000
Fiber Area (μm2)
L Myh3+/+
M Myh3Δ/Δ
N Myh3+/+
T1
T5
T9
T13
O Myh3Δ/Δ
Fused (C6-T1)
Fused (T3-T4)
Fused (T6-T8)
T13
P
Cervical
Thoracic
Lumbar
C1
C5
Fused (C6-T1)
Fused (T3-T4)
Fused (T6-T8)
T9
T13
L1
Fused (L3-L5)
L6
S 12-14 weeks
Latency to fall (s)
0.0026 **
T 12-14 weeks
Running time (seconds)
<0.0001 ****
Q
% Fusion
<0.0001 ****
Thoracic vertebrae
R
Grip strength normalized to body weight (gf/gms)
0.0199 *
≤0.0001 ****
≤0.0001 ****
≤0.0001 ****
0.00048 ***
Age in weeks

Figure 1.

Figure 1. Loss of MyHC-embryonic leads to adult muscle and skeletal defects in mice.

A, B Total body weight (grams) of control (*Myh3*$^{+/+}$) and MyHC-embryonic knockout (*Myh3*$^{\Delta/\Delta}$) male mice measured at 8–10 weeks and 6 months of age.

C–F Individual muscle weight of tibialis anterior (TA) and gastrocnemius (Gastroc) of *Myh3*$^{+/+}$ and *Myh3*$^{\Delta/\Delta}$ male mice measured at 8–10 weeks and 6 months of age, respectively.

G, H Quantification of the number of myofibers normalized to cross-sectional area (CSA) (mm^2) through the TA muscle of *Myh3*$^{+/+}$ and *Myh3*$^{\Delta/\Delta}$ mice at 8–10 weeks and 6 months of age, respectively (n = 5 mice per genotype).

I, J Representative fluorescent micrographs of transverse sections through the TA muscle of *Myh3*$^{+/+}$ and *Myh3*$^{\Delta/\Delta}$ mice at 6 months of age stained with Laminin (red), and DAPI (blue).

K Quantification of the number of myofibers grouped according to myofiber area through the TA muscle of *Myh3*$^{+/+}$ and *Myh3*$^{\Delta/\Delta}$ mice at 6 months of age (n = 5 mice per genotype).

L, M MicroCT images through the dorsal region of representative *Myh3*$^{+/+}$ and *Myh3*$^{\Delta/\Delta}$ mice.

N, O Bright-field images of whole-mount skeletal preparations of representative *Myh3*$^{+/+}$ and *Myh3*$^{\Delta/\Delta}$ 4-month-old mice stained with Alcian blue and Alizarin red.

P Schematic representation depicting the vertebral fusion observed in a representative *Myh3*$^{\Delta/\Delta}$ mouse shown in panel O.

Q Quantification of the percentage of thoracic vertebral fusion in *Myh3*$^{+/+}$ and *Myh3*$^{\Delta/\Delta}$ mice.

R Quantification of grip strength of *Myh3*$^{+/+}$ and *Myh3*$^{\Delta/\Delta}$ mice normalized to body weight over the 12–24-week time course.

S Rotarod analysis of 12–14-week-old *Myh3*$^{+/+}$ and *Myh3*$^{\Delta/\Delta}$ mice where each dot depicts the mean time of latency to fall (in seconds) of individual mice.

T Graph quantifying treadmill exhaustion (in seconds) of 12–14-week-old *Myh3*$^{+/+}$ and *Myh3*$^{\Delta/\Delta}$ mice.

Data information: Data are presented as mean ± SEM. Student's t-test was performed, with $P \leq 0.05$ considered significant. Scale bar: 100 μm (J).
Source data are available online for this figure.

into discrete bins according to their cross-sectional area and the number of myofibers falling in each group quantified. We observed a significant increase in the number of smaller-sized myofibers (1,000–1,500 μm^2 bin) and a significant decrease in the number of larger-sized myofibers (2,500–3,000, 3,000–3,500, 3,500–4,000, and 4,000–4,500 μm^2 bins) in the TA from 6-month-old *Myh3*$^{\Delta/\Delta}$ mice (Fig 1I–K). Similar results were observed for 8–10-week-old TA (Fig EV1E), 8–10 week and 6-month-old gastrocnemius and soleus muscles from *Myh3*$^{\Delta/\Delta}$ mice (Fig EV1F–I). Scoliosis was observed in all *Myh3*$^{\Delta/\Delta}$ mice, which became apparent by 6 weeks of age and persisted thereafter; an example at 8–10 weeks along with a control, imaged using microCT is shown (Fig 1L and M). Vertebral fusion was also seen in *Myh3*$^{\Delta/\Delta}$ mice in the cervical (Fig EV1J and K), thoracic (Fig 1N–P), and lumbar region, with severe reduction in intervertebral disc region observed between fused vertebrae (Fig 1O and P). Upon quantification, approximately 50, 78, and 82% fused vertebrae were seen in the cervical, thoracic, and lumbar regions, respectively, in *Myh3*$^{\Delta/\Delta}$ mice (Figs 1Q and EV1L and M). At the level of muscle function, *Myh3*$^{\Delta/\Delta}$ mice exhibited significantly reduced grip strength normalized to body weight compared with controls, consistently, over 2-week time points from 12 to 24 weeks of age (Fig 1R). In functional activity assays, *Myh3*$^{\Delta/\Delta}$ mice exhibited reduced latency to fall in rotarod tests (Fig 1S) and reduced time to exhaustion (Fig 1T) as well as distance ran (Fig EV1N) in treadmill tests. The myosin ATPase activity was found to be significantly reduced in embryonic day (E) 16.5 myosin extracts from *Myh3*$^{\Delta/\Delta}$ mice compared with controls (Fig EV1O).

MyHC-embryonic function is required for adult muscle fiber type maintenance

Previously, we reported that loss of MyHC-embryonic function leads to increased MyHC-slow+ fiber numbers and elevated MyHC-slow and -IIa protein levels in neonatal stages (Agarwal *et al*, 2020). Interestingly, although the increased MyHC-slow+ fiber numbers persisted at postnatal day (P) 15 in *Myh3*$^{\Delta/\Delta}$ mice, there were no significant differences by P30 (Agarwal *et al*, 2020).

To characterize the effect of MyHC-embryonic on adult muscle fiber type, we carried out immunofluorescence labeling for adult MyHC isoforms, on cross sections through the TA muscle of control *Myh3*$^{+/+}$ and *Myh3*$^{\Delta/\Delta}$ mice, at early (8–10 week) and late (6-month) time points. The percentage of MyHC-IIb+ and MyHC-IIa+ fiber numbers compared with total fiber numbers were increased significantly in *Myh3*$^{\Delta/\Delta}$ mice at both time points, indicating that MyHC-embryonic is required for proper adult fiber type maintenance (Fig 2A–H). No significant effect was observed on the percentage of MyHC-IIx+ fibers in the TA in *Myh3*$^{\Delta/\Delta}$ mice (Fig 2I–L). Since the TA has very few MyHC-slow+ fibers, we characterized the effect of loss of MyHC-embryonic on MyHC-slow fibers in the gastrocnemius, where we observed a significant reduction in the percentage of MyHC-slow+ fibers in *Myh3*$^{\Delta/\Delta}$ mice, both at 8–10 weeks and 6 months (Fig 2M–P). The soleus is one of the muscles with a high percentage of MyHC-slow+ fibers, where we observed no significant differences in the percentage of MyHC-slow+ fibers in *Myh3*$^{\Delta/\Delta}$ mice at the 8–10-week time point (Fig EV2A). Interestingly, the percentage of MyHC-slow+ fibers exhibited a significant decrease in *Myh3*$^{\Delta/\Delta}$ mice at the 6-month time point (Fig EV2B).

We found that MyHC-IIb and -IIa protein levels were elevated in *Myh3*$^{\Delta/\Delta}$ mice TA muscle by western blots and densitometry, at 8–10 weeks and 6 months (Fig 2Q–U). MyHC-slow protein levels were reduced in *Myh3*$^{\Delta/\Delta}$ mice gastrocnemius muscle by western blots and densitometry, at 8–10 weeks and 6 months (Fig 2V–X). Thus, these results validate the immunofluorescence data with respect to the effect of MyHC-embryonic on adult muscle fiber type. Western blots indicated that MyHC-IIb protein levels were elevated at both time points, whereas MyHC-IIa protein levels were increased only at the 8–10-week time point and not at 6 months, in the gastrocnemius of *Myh3*$^{\Delta/\Delta}$ mice (Fig EV2C–G).

These results demonstrate that MyHC-embryonic function is crucial for adult muscle fiber type maintenance, with the most consistent effect seen on MyHC-IIb fibers, which were increased in the TA and gastrocnemius at both time points (Figs 2A–D, Q, R, and T and EV2C, E, and G). Since MyHC-IIb is the predominant fiber type present in both TA and gastrocnemius, minor differences are unlikely to be statistically significant, suggesting that loss of MyHC-embryonic has the clearest effect on MyHC-IIb fiber type. Loss of MyHC-embryonic had perhaps the strongest effect on MyHC-slow, with the percentage of MyHC-slow+ fibers decreased to half and

 © 2023 The Authors

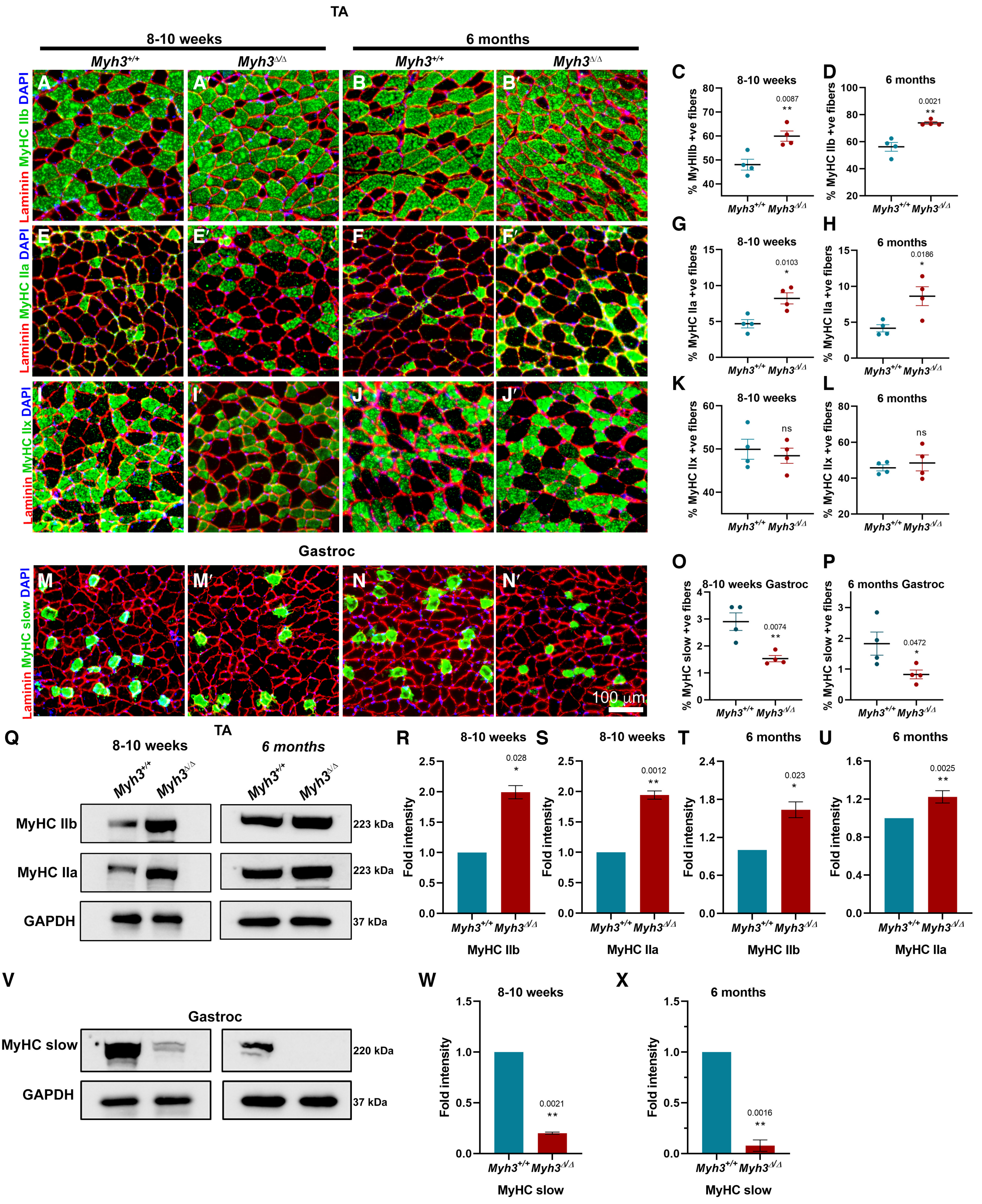

TA
8-10 weeks
6 months
Myh3+/+
Myh3Δ/Δ
Laminin MyHC IIb DAPI
Laminin MyHC IIa DAPI
Laminin MyHC IIx DAPI
Gastroc
Laminin MyHC slow DAPI
100 μm
% MyHIIb +ve fibers
% MyHC IIb +ve fibers
% MyHC IIa +ve fibers
% MyHC IIx +ve fibers
% MyHC slow +ve fibers
8-10 weeks Gastroc
6 months Gastroc
0.0087
0.0021
0.0103
0.0186
ns
0.0074
0.0472
MyHC IIb
MyHC IIa
GAPDH
223 kDa
37 kDa
Fold intensity
0.028
0.0012
0.023
0.0025
MyHC slow
220 kDa
0.0021
0.0016

Figure 2.

Figure 2. ***Myh3***$^{\Delta/\Delta}$ **mice exhibit muscle fiber type changes.**

A–D Representative fluorescent micrographs of transverse sections through the TA muscle of *Myh3*$^{+/+}$ and *Myh3*$^{\Delta/\Delta}$ mice at 8–10 weeks and 6 months of age stained with Laminin (red), MyHC-IIb (green) and DAPI (blue) (A–B′), and quantification of the percentage of MyHC-IIb-positive fibers (C, D).

E–H Representative fluorescent micrographs of transverse sections through the TA muscle of *Myh3*$^{+/+}$ and *Myh3*$^{\Delta/\Delta}$ mice at 8–10 weeks and 6 months of age stained with Laminin (red), MyHC-IIa (green) and DAPI (blue) (E–F′), and quantification of the percentage of MyHC-IIa-positive fibers (G, H).

I–L Representative fluorescent micrographs of transverse sections through the TA muscle of *Myh3*$^{+/+}$ and *Myh3*$^{\Delta/\Delta}$ mice at 8–10 weeks and 6 months of age stained with Laminin (red), MyHC-IIx (green) and DAPI (blue) (I–J′), and quantification of the percentage of MyHC-IIx-positive fibers (K, L).

M–P Representative fluorescent micrographs of transverse sections through the gastrocnemius muscle of *Myh3*$^{+/+}$ and *Myh3*$^{\Delta/\Delta}$ mice at 8–10 weeks and 6 months of age stained with Laminin (red), MyHC-slow (green) and DAPI (blue) (M–N′), and quantification of the percentage of MyHC-slow-positive fibers (O, P).

Q–U Representative western blots for MyHC-IIb, -IIa, and GAPDH using protein lysates from the TA muscle of *Myh3*$^{+/+}$ and *Myh3*$^{\Delta/\Delta}$ mice at 8–10 weeks and 6 months of age (Q) and their densitometric quantification (R–U) (n = 4 mice per genotype).

V–X Representative western blots for MyHC-slow and GAPDH using protein lysates from the gastrocnemius muscle of *Myh3*$^{+/+}$ and *Myh3*$^{\Delta/\Delta}$ mice at 8–10 weeks and 6 months of age (V) and their densitometric quantification (W, X) (n = 4 mice per genotype).

Data information: Quantification of MyHC-positive fibers is represented as percent of MyHC-positive fibers normalized to total number of fibers. Data are presented as mean ± SEM. Student's t-test was performed, with $P \leq 0.05$ considered significant. Scale bar: 100 μm (N′).
Source data are available online for this figure.

MyHC-slow protein levels reduced by about fivefold, in the gastrocnemius at both time points (Fig 2M–P and V–X). The percentage of MyHC-slow+ fibers were not affected in the soleus at 8–10 weeks, but was significantly reduced at 6 months (Fig EV2A and B). This indicates that some of the effects of MyHC-embryonic on adult muscle fiber type are age- and muscle-dependent. *Myh3*$^{\Delta/\Delta}$ mice also exhibited an increase in MyHC-IIa fiber type in the TA at both time points, the effect was restricted to the early time point in the gastrocnemius, confirming that loss of MyHC-embryonic function has distinct effects on adult fiber type in different muscles (Figs 2E–H, Q, S, and U and EV2C, D, and F).

Loss of MyHC-embryonic function results in altered satellite cell numbers and increased fibrosis

Loss of MyHC-embryonic function led to an increase in Pax7+ muscle stem (satellite) cell numbers per unit area, in the 8–10-week-old TA (Fig 3A–C). Satellite cell numbers were increased at P30, an earlier time point, in the TA of *Myh3*$^{\Delta/\Delta}$ mice (Fig EV3A–C). However, by 6 months of age, *Myh3*$^{\Delta/\Delta}$ mice exhibited a significant reduction in satellite cell numbers in the TA (Fig 3D–F). These results were confirmed by western blots followed by densitometry of the TA, where an increase in Pax7 levels was observed at 8–10 weeks and a reduction at 6 months in *Myh3*$^{\Delta/\Delta}$ mice (Fig 3G–I). In the gastrocnemius, Pax7 levels were decreased in *Myh3*$^{\Delta/\Delta}$ mice at both the 8–10-week and 6-month time points (Fig EV3D–F). Previously, we had found that Pax7 levels were significantly reduced upon embryonic and fetal-specific *Myh3* knockout, which was back to normal by P0 (Agarwal *et al*, 2020). Thus, loss of MyHC-embryonic leads to a postnatal increase in satellite cell numbers and Pax7 levels in the TA, followed by a decline at the 6-month later time point (Figs 3A–I and EV3A–C). Unlike the TA, the gastrocnemius exhibited a decrease in Pax7 levels at both the 8–10-week and 6-month time points, suggesting that loss of MyHC-embryonic has distinct effects on satellite cell numbers in different muscles.

The altered satellite cell numbers could be because they are activated early on, leading to exhaustion of the satellite cell pool. It is also possible that satellite cells in *Myh3*$^{\Delta/\Delta}$ mice undergo increased apoptosis, resulting in their depletion. To distinguish between these, protein levels of the differentiation marker MyoD and the apoptotic marker Caspase3 were quantified by western blots of the TA at 8–10 weeks and 6 months (Fig 3G). Interestingly, densitometry showed a significant increase in MyoD levels at the 8–10-week time point and a significant reduction at the 6-month time point in *Myh3*$^{\Delta/\Delta}$ mice, indicating that the satellite cells in *Myh3*$^{\Delta/\Delta}$ mice are activated at early time points, leading to their depletion by later stages (Fig 3G, J, and K). Caspase3 levels exhibited a decrease at the 8–10-week time point and no change at 6 months in *Myh3*$^{\Delta/\Delta}$ mice, suggesting that increased apoptosis does not lead to the altered satellite cell numbers in *Myh3*$^{\Delta/\Delta}$ mice (Fig 3G, L, and M). To further validate this, individual muscle fibers along with resident satellite cells were isolated from the extensor digitorum longus (EDL) muscle of 8–10-week-old *Myh3*$^{+/+}$ and *Myh3*$^{\Delta/\Delta}$ mice, cultured, and labeled for MyoD as a marker for satellite cell activation (Fig 3N–O″). Significantly elevated number of activated Pax7+ MyoD+ satellite cells were observed at 6 and 12 h after fiber isolation in *Myh3*$^{\Delta/\Delta}$ mice, confirming that satellite cells are indeed activated faster in *Myh3*$^{\Delta/\Delta}$ mice (Fig 3N–P).

We also observed that the area occupied by connective tissue (marked by red in Sirius red staining) was at least double in the *Myh3*$^{\Delta/\Delta}$ TA compared with controls, at both 8–10 weeks and 6 months (Fig 3Q–V). This suggests that loss of MyHC-embryonic leads to increased fibrosis, possibly explaining the altered satellite cell activation status and numbers.

Cytoskeletal regulation and mechanotransduction pathways are dysregulated upon loss of MyHC-embryonic function

To further characterize the effect of loss of MyHC-embryonic function, we carried out a mass spectrometric experiment comparing protein expression from the hind limb skeletal muscle of E16.5 *Myh3*$^{+/+}$ and *Myh3*$^{\Delta/\Delta}$ embryos. Upon analysis, 42 proteins were uniquely expressed in *Myh3*$^{+/+}$, 77 in *Myh3*$^{\Delta/\Delta}$ and 394 were common to both genotypes (Fig 4A). Of the 394 proteins expressed in *Myh3*$^{+/+}$ and *Myh3*$^{\Delta/\Delta}$ embryos, 48 were downregulated and 61 upregulated in the *Myh3*$^{\Delta/\Delta}$ muscle (Fig 4A). Further analysis of signaling pathways and processes identified proteins involved in integrin signaling, cytoskeletal regulation by RhoGTPase, ubiquitin-proteasome, and FGF signaling as the four pathways most affected upon loss of MyHC-embryonic function (Fig 4B). The presence of FGF signaling validates our previous study, where we had reported the role of FGF signaling in regulating the rate of differentiation upon loss of MyHC-embryonic function (Agarwal *et al*, 2020).

 © 2023 The Authors

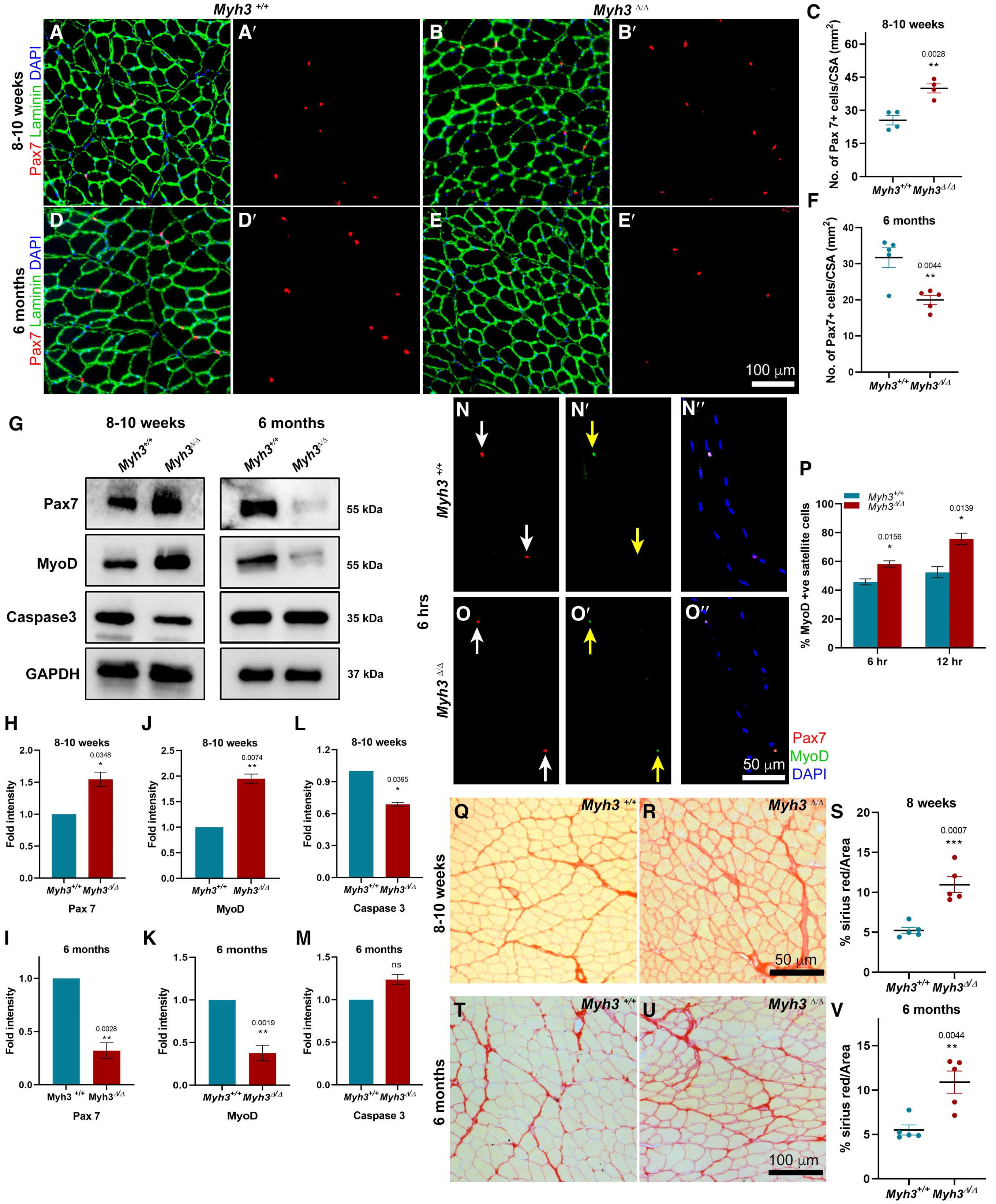

Myh3 +/+
Myh3 Δ/Δ
8-10 weeks
6 months
Pax7 Laminin DAPI
100 µm
8-10 weeks
No. of Pax 7+ cells/CSA (mm²)
0.0028
**
6 months
No. of Pax7+ cells/CSA (mm²)
0.0044
**
Pax7
MyoD
Caspase3
GAPDH
55 kDa
55 kDa
35 kDa
37 kDa
Fold intensity
0.0348
0.0074
0.0395
0.0028
0.0019
ns
Pax 7
MyoD
Caspase 3
6 hrs
Pax7
MyoD
DAPI
50 µm
% MyoD +ve satellite cells
0.0156
0.0139
6 hr
12 hr
8 weeks
% sirius red/Area
0.0007
***
0.0044
**

Figure 3.

Figure 3. *Myh3*$^{\Delta/\Delta}$ mice exhibit alterations in satellite cell numbers and increased fibrosis.

A–F Representative fluorescent micrographs of transverse sections through the TA muscle of *Myh3*$^{+/+}$ and *Myh3*$^{\Delta/\Delta}$ mice at 8–10 weeks and 6 months of age stained with Pax7 (red), Laminin (green) and DAPI (blue) (A–B′, D–E′), and quantification of the number of Pax7-positive cells per unit area (mm^2) (C, F).

G–M Representative western blots for Pax7, MyoD, Caspase 3, and GAPDH using protein lysates from the TA muscle of *Myh3*$^{+/+}$ and *Myh3*$^{\Delta/\Delta}$ mice at 8–10 weeks and 6 months of age (G) and their densitometric quantification (H-M) (n = 4 mice per genotype).

N–P Representative fluorescent micrographs of single myofibers isolated and cultured for 6 h from 8–10 week-old *Myh3*$^{+/+}$ and *Myh3*$^{\Delta/\Delta}$ mice, stained for Pax7 (red), MyoD (green) and DAPI (blue) (N–O″), and quantification of Pax7+ MyoD+ satellite cells at 6 and 12 h of culture (P) (n = 3 mice per genotype).

Q–V Representative bright-field micrographs of transverse sections through the TA muscle of *Myh3*$^{+/+}$ and *Myh3*$^{\Delta/\Delta}$ mice at 8–10 weeks and 6 months of age stained for Sirius red to label the extracellular matrix (Q, R, T, U), and quantification of the percentage of Sirius red-positive area as a fraction of total area (S, V).

Data information: Data are presented as mean ± SEM. Student's t-test was performed, with $P \leq 0.05$ considered significant. Scale bar: 100 μm (E′, U); 50 μm (O″, R). Source data are available online for this figure.

Integrins are transmembrane proteins that connect the cellular cytoskeleton to the extracellular matrix, performing a wide range of functions such as adhesion, signaling etc. (Kechagia *et al*, 2019). Integrins are crucial for skeletal muscle function, with integrin α7 mutations leading to congenital myopathy in humans (Hayashi *et al*, 1998); integrin α7 knockout mice (Mayer *et al*, 1997), and integrin α5 mutant chimeric mouse embryos (Taverna *et al*, 1998) exhibit novel muscular dystrophy symptoms, and β1-integrin deficient myoblasts show myoblast fusion and sarcomere assembly defects (Schwander *et al*, 2003). Integrins are localized primarily to the myotendinous junctions and costameres in the skeletal muscle and are crucial for sensing, responding to, and transmitting mechanical forces (Boppart & Mahmassani, 2019). Talin, filamin, and α-actinin are integrin-binding cytoplasmic proteins that connect to the actin cytoskeleton (Calderwood *et al*, 2001). Interestingly, we observed that Talin1 and Talin 2, key cytoplasmic proteins that link integrins to the actin cytoskeleton, are upregulated in *Myh3*$^{\Delta/\Delta}$ muscle (Fig 4C). Previous studies indicate that mouse knockouts for Talin1 or Talin2 exhibit myopathy, and defects in myotendinous junctions, myoblast fusion and sarcomere assembly, phenotypes similar to integrin mutant mice (Conti *et al*, 2008, 2009). We also find that FLNB, a filamin protein, is detected only in the control *Myh3*$^{+/+}$ samples and not in *Myh3*$^{\Delta/\Delta}$ muscle (Fig 4C). Interestingly, filamins are known to compete with talins to bind β-integrin cytoplasmic tails, which can influence integrin activation and signaling (Kiema *et al*, 2006). We also found several cytoskeletal proteins that were dysregulated in the *Myh3*$^{\Delta/\Delta}$ muscle (Fig 4D). Proteins under the category "cytoskeletal regulation by RhoGTPase," which was the second most affected pathway upon loss of MyHC-embryonic, are also known to crosstalk with integrins to regulate their signaling, activation, and mechanotransduction (Parsons *et al*, 2010; Lawson & Burridge, 2014).

Upon observing the dysregulation of key proteins in the integrin and RhoGTPase pathways in the *Myh3*$^{\Delta/\Delta}$ muscle, we hypothesized that loss of MyHC-embryonic might affect mechanotransduction. Western blots and densitometry confirmed the upregulation of Talin1 and reduction in Filamin B levels in *Myh3*$^{\Delta/\Delta}$ muscle seen in the mass spectrometry experiment (Fig 4E and F). Several studies indicate that the Yes-associated protein (YAP) signaling pathway functions downstream of integrin signaling in diverse contexts, for instance in skin homeostasis (Elbediwy *et al*, 2016), skeletal stem cell lineage commitment (Tang *et al*, 2013), prostate cancer (Varzavand *et al*, 2016), vascular remodeling (Yamashiro *et al*, 2020), atherosclerosis (Wang *et al*, 2016), and cancer cell invasion (Liu *et al*, 2018). Therefore, we studied YAP expression in skeletal muscle lysates from *Myh3*$^{+/+}$ and *Myh3*$^{\Delta/\Delta}$ E16.5 embryos, where we observed a significant, twofold upregulation of YAP levels upon loss of MyHC-embryonic (Fig 4E and F). One of the most upregulated candidate proteins in the *Myh3*$^{\Delta/\Delta}$ muscle in our mass spectrometric analysis was neural precursor cell expressed developmentally down-regulated 4 (NEDD4), an E3 ubiquitin ligase. We validated the mass spectrometric analysis by western blots, where we found NEDD4 levels to be significantly elevated in the *Myh3*$^{\Delta/\Delta}$ muscle (Fig 4E and F). Interestingly, NEDD4 has been shown to regulate components of the Hippo pathway, such as the serine/threonine kinase large tumor suppressor (LATS), essential for YAP localization and transcriptional activity (Salah *et al*, 2013; Bae *et al*, 2015; Jeon *et al*, 2019). Increased NEDD4 levels lead to LATS degradation and increased YAP activation (Salah *et al*, 2013; Bae *et al*, 2015; Jeon *et al*, 2019). To confirm the increased YAP activity, we carried out a luciferase reporter assay by transfecting the YAP responsive *8xGTIIC-luciferase* plasmid into C2C12 mouse myogenic cells treated with a control siRNA or *Myh3* siRNA and allowing the cells to differentiate for 24 h (Dupont *et al*, 2011). Significantly elevated luciferase activity was observed in the *Myh3* siRNA-treated C2C12 cells, confirming that loss of MyHC-embryonic function leads to increased YAP activity (Fig 4G). These results were validated by western blots for total YAP which was elevated and phosphorylated YAP (S109), its inactive form, which was significantly decreased upon *Myh3* knockdown in C2C12 cells (Fig 4H and I). To test whether this effect on YAP is unique to MyHC-embryonic, we depleted another developmental MyHC, MyHC-perinatal encoded by the *Myh8* gene. This resulted in increased total YAP and reduced phosphorylated YAP (S109), indicating that in addition to MyHC-embryonic, other developmental MyHCs are also capable of regulating YAP (Fig 4J and K). Thus, our results suggest that integrin and cytoskeletal pathway dysregulation in the skeletal muscle of *Myh3*$^{\Delta/\Delta}$ embryos result in altered mechanotransduction leading to elevated YAP activation.

YAP signaling is dysregulated in the skeletal muscle of postnatal and adult *Myh3*$^{\Delta/\Delta}$ mice

Next, we wanted to study YAP signaling at postnatal and adult stages to decipher whether the musculoskeletal defects seen in *Myh3*$^{\Delta/\Delta}$ mice are linked to altered YAP pathway activation. Western blots and densitometry for total YAP indicated significantly elevated levels in the *Myh3*$^{\Delta/\Delta}$ muscle at postnatal day (P) 0 and 4-month-old adult stages (Fig 5A and B). Serine phosphorylation of YAP is known to inactivate YAP by preventing its nuclear localization or promoting its degradation (Basu *et al*, 2003; Zhao *et al*, 2007, 2010; Llado *et al*, 2015; Meng *et al*, 2015; Zhang *et al*, 2015). Therefore, we tested the levels of phosphorylated YAP

 © 2023 The Authors

using the S109- and S127-phospho-specific YAP antibodies, which upon densitometry showed consistent decrease in the *Myh3*$^{\Delta/\Delta}$ muscle at P0 and 4-month stages (Fig 5A, C, and D). The reduced levels of phosphorylated YAP indicate that YAP signaling is upregulated upon loss of MyHC-embryonic function.

Since YAP localization is serine phosphorylation-dependent, we next isolated the nuclear and cytoplasmic fractions from the muscle of 4-month-old *Myh3*$^{+/+}$ and *Myh3*$^{\Delta/\Delta}$ mice, which were then subjected to western blotting for YAP, H3A as nuclear marker and GAPDH as cytoplasmic marker (Fig 5E and F). The nuclear fraction

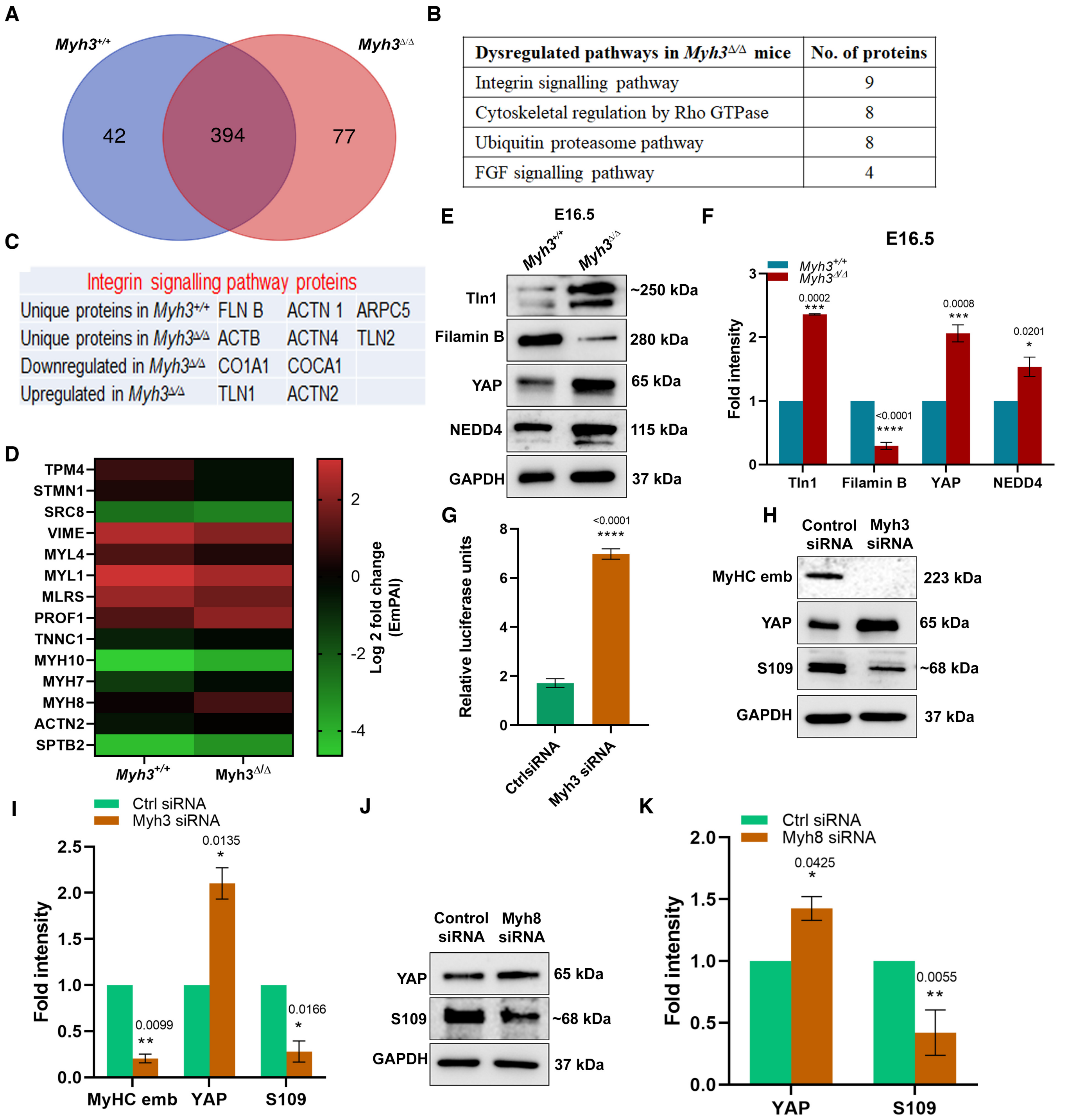

Figure 4.

◀ **Figure 4. Cytoskeletal regulation and mechanotransduction pathways are dysregulated in *Myh3*$^{\Delta/\Delta}$ embryos.**

- A Venn diagram depicting the number of unique and shared proteins expressed in the hind limb muscles of *Myh3*$^{+/+}$ and *Myh3*$^{\Delta/\Delta}$ embryos at embryonic day (E)16.5 (n = 4 mice per genotype).
- B Table showing key dysregulated pathways and the number of proteins affected in each, in *Myh3*$^{+/+}$ and *Myh3*$^{\Delta/\Delta}$ embryos at E16.5.
- C Integrin signaling pathway proteins mis-regulated in *Myh3*$^{+/+}$ and *Myh3*$^{\Delta/\Delta}$ E16.5 embryos.
- D Heat map of differentially regulated proteins involved in cytoskeletal reorganization quantified using EmPAI value, in *Myh3*$^{+/+}$ and *Myh3*$^{\Delta/\Delta}$ embryos at E16.5.
- E, F Representative western blots for Talin1 (n = 3 mice per genotype), Filamin B (n = 5 mice per genotype), YAP (n = 5 mice per genotype), NEDD4 (n = 5 mice per genotype), and GAPDH using protein lysates from the hind limb muscle of *Myh3*$^{+/+}$ and *Myh3*$^{\Delta/\Delta}$ embryos at E16.5 (E) and their densitometric quantification (F).
- G Luciferase reporter assay for the YAP responsive *8xGTIIC-luciferase* plasmid transfected into C2C12 cells treated with control or *Myh3* siRNA (n = 3).
- H, I Representative western blots for MyHC-embryonic (MyHC-emb), YAP, phospho-YAP S109, and GAPDH using protein lysates from C2C12 cells transfected with control or *Myh3* siRNA (H) and their densitometric quantification (I) (n = 3).
- J, K Representative western blots for YAP, phospho-YAP S109, and GAPDH using protein lysates from C2C12 cells transfected with control or *Myh8* siRNA (J) and their densitometric quantification (K) (n = 3).

Data information: Data are presented as mean ± SEM. Student's t-test was performed, with $P \leq 0.05$ considered significant.
Source data are available online for this figure.

exhibited ~threefold increase in YAP levels whereas the cytoplasmic fraction showed reduced YAP levels in *Myh3*$^{\Delta/\Delta}$ samples, substantiating that the reduced levels of phosphorylated YAP in *Myh3*$^{\Delta/\Delta}$ mice indeed led to increased nuclear accumulation of YAP (Fig 5E–H). To confirm that the increased nuclear YAP levels led to the upregulation of YAP signaling, we checked the transcript expression of two YAP target genes, connective tissue growth factor (*CTGF*) and cysteine-rich angiogenic inducer 61 (*CYR61*) by qPCR (Meng *et al*, 2015), and found both to be upregulated in the muscle of 4-month-old *Myh3*$^{\Delta/\Delta}$ mice (Fig 5I). Western blots for CTGF and CYR61 also showed significantly increased expression for both proteins in muscle protein lysates from 4-month-old *Myh3*$^{\Delta/\Delta}$ mice, validating these findings (Fig 5J–M). The transcript levels of Angiomotin (*Amot*), MOB kinase activator 1A (*Mob1a*), and MOB kinase activator 1B (*Mob1b*), which are upstream regulators of YAP in the Hippo signaling pathway (Zhao *et al*, 2010; Chan *et al*, 2011; Nishio *et al*, 2016), were significantly reduced in the muscle of 4-month-old *Myh3*$^{\Delta/\Delta}$ mice (Fig 5N).

Next, we tested whether the effect on YAP observed upon loss of MyHC-embryonic function is recapitulated by inhibitors of cellular contraction. Treatment with para-aminoblebbistatin, a myosin inhibitor, resulted in increased total YAP and reduced phosphorylated YAP (S109) in C2C12 cells (Fig EV4A and B). Treatment with 2,3-Butanedione monoxime (BDM), another contractility inhibitor, led to no change in total YAP but increased phosphorylated YAP (S109) levels (Fig EV4C and D). Thus, treatment with inhibitors of cellular contractility leads to altered YAP levels and activity in myogenic cells.

Cell contraction was measured by culturing C2C12 cells treated with control or *Myh3* siRNA on a collagen matrix, followed by the release of the matrix (Fig EV4E). A significant reduction in contraction was observed in *Myh3* siRNA-treated cells compared with controls, validating the altered contractility upon loss of MyHC-embryonic function (Fig EV4F).

Levels of the YAP paralog TAZ were unchanged in the cytoplasmic and nuclear fractions and in the total lysate in muscle protein lysates from 4-month-old *Myh3*$^{\Delta/\Delta}$ mice (Fig EV4G–L). Levels of TEAD, the YAP/TAZ interacting transcription factors, were undetectable in the cytoplasmic fraction and unchanged in the nuclear fraction as well as total lysate in muscle protein lysates from 4-month-old *Myh3*$^{\Delta/\Delta}$ mice (Fig EV4G–L). Thus, the mechanotransduction-mediated effects of loss of MyHC-embryonic function specifically affect YAP and not TAZ or TEAD.

Overall, these results indicate that YAP signaling is upregulated in early postnatal and adult *Myh3*$^{\Delta/\Delta}$ mouse skeletal muscle and may underlie the musculoskeletal defects seen upon loss of MyHC-embryonic function.

Inhibition of YAP signaling by CA3 rescues the musculoskeletal defects exhibited by *Myh3*$^{\Delta/\Delta}$ mice

Since we observed increased YAP pathway activation in *Myh3*$^{\Delta/\Delta}$ mice, we next tested whether inhibiting the YAP pathway might rescue the defects seen in these mice. We used the small molecule CA3, which has been reported to be a potent inhibitor of YAP activity (Song *et al*, 2018; Kandasamy *et al*, 2020). We found that CA3 treatment at 1 mg/kg body weight, starting at P15, twice a week for 6 weeks, followed by analysis at 12–14 weeks of age led to a significant increase in the grip strength in *Myh3*$^{\Delta/\Delta}$ mice, compared with vehicle-treated mice (Figs 6A and EV5A). Control *Myh3*$^{+/+}$ mice did not exhibit any significant change in grip strength upon CA3 treatment (Fig EV5B). The number of myofibers per unit area (mm^2) exhibited a significant decrease, suggesting an increase in the proportion of larger myofibers upon CA3 treatment (Fig 6B). This turned out to be the case when we grouped the myofibers according to their cross-sectional area, where the proportion of myofibers with > 2,500 mm^2 significantly increased upon CA3 treatment (Fig 6C). The elevated fibrosis measured by Sirius red staining seen in *Myh3*$^{\Delta/\Delta}$ mice was significantly reduced in the TA muscle upon CA3 treatment compared with vehicle (Fig 6D–F). Next, we studied the levels of MyHC and Pax7 proteins upon CA3 treatment in the TA; MyHC-IIa levels which were increased in the TA of *Myh3*$^{\Delta/\Delta}$ mice showed a significant reduction upon CA3 treatment (Fig 6G–H), MyHC-IIb levels which were increased in the TA of *Myh3*$^{\Delta/\Delta}$ mice showed no change upon CA3 treatment (Fig 6G and I), whereas levels of Pax7, which was increased at early time points in the TA showed a significant reduction upon CA3 treatment (Fig 6G and J). MyHC-slow levels which were downregulated in the gastrocnemius of *Myh3*$^{\Delta/\Delta}$ mice exhibited a significant increase upon CA3 treatment (Fig 6K and L). Interestingly, levels of Pax7 which were decreased consistently in the gastrocnemius of *Myh3*$^{\Delta/\Delta}$ mice showed the opposite trend upon CA3 treatment, where Pax7 levels were elevated (Fig 6K and M). The proportion of MyHC-slow+ fibers also exhibited a significant increase in the soleus upon CA3 treatment (Fig 6N). Expectedly, CA3 treatment did not alter the levels of YAP in the TA or gastrocnemius, since it has been reported to

© 2023 The Authors

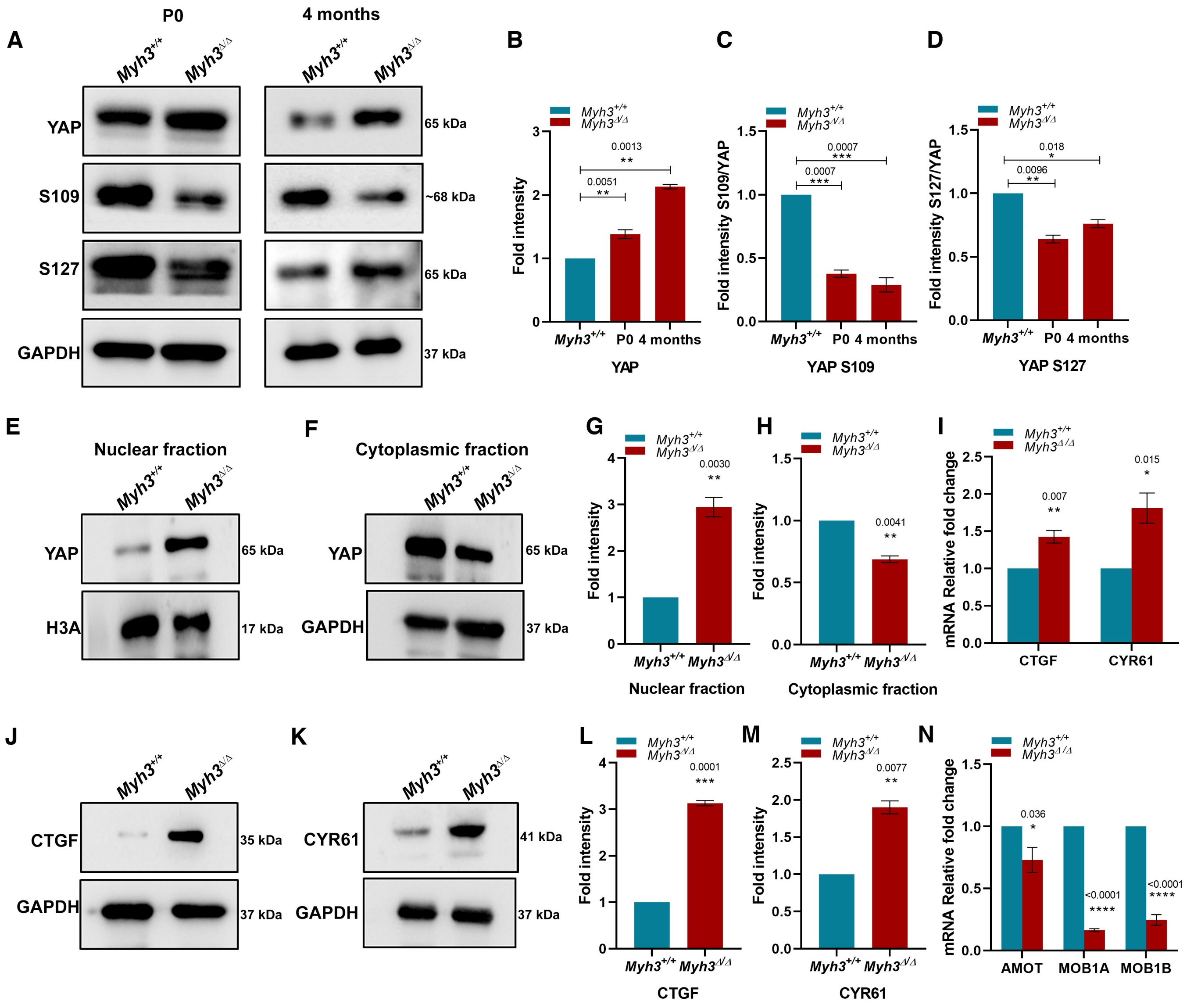

Figure 5. YAP signaling is activated in the skeletal muscle of *Myh3*$^{\Delta/\Delta}$ postnatal and adult mice.

A–D Representative western blots for total YAP, phospho-YAP (S109 and S127), and GAPDH using protein lysates from the TA muscle of postnatal day (P) 0 and 4-month-old *Myh3*$^{+/+}$ and *Myh3*$^{\Delta/\Delta}$ mice (A) and their densitometric quantification (B–D) (n = 4 mice per genotype).

E–H Representative western blots for total YAP and histone H3A in the nuclear fraction (E), and total YAP and GAPDH in the cytoplasmic fraction (F), isolated from the TA muscle of 4-month-old *Myh3*$^{+/+}$ and *Myh3*$^{\Delta/\Delta}$ mice and their densitometric quantification (G, H) (n = 6 mice per genotype).

I Graph depicting the transcript expression of *CTGF* and *CYR61* in the TA muscle of 4-month-old *Myh3*$^{+/+}$ and *Myh3*$^{\Delta/\Delta}$ mice (n = 3 mice per genotype).

J–M Representative western blots for CTGF and GAPDH (J), and CYR61 and GAPDH (K), using protein lysates from the TA muscle of 4-month-old *Myh3*$^{+/+}$ and *Myh3*$^{\Delta/\Delta}$ mice and their densitometric quantification (L, M) (n = 4 mice per genotype).

N Graph showing the transcript expression of *AMOT*, *MOB1A*, and *MOB1B* in the TA muscle of 4-month-old *Myh3*$^{+/+}$ and *Myh3*$^{\Delta/\Delta}$ mice (n = 4 mice per genotype).

Data information: Data are presented as mean ± SEM. Student's t-test was performed, with $P \leq 0.05$ considered significant.
Source data are available online for this figure.

inhibit YAP activity rather than levels (Song *et al*, 2018; Fig 6O–R). Levels of the YAP targets CTGF and Cyr61 were significantly reduced in both TA and gastrocnemius, validating that CA3 treatment indeed led to decreased YAP signaling (Fig 6S–V). Treatment with CA3 led to a significant increase in MyHC-IIa protein levels but did not significantly affect the levels of MyHC-IIb, Pax7, YAP, or CTGF in control *Myh3*$^{+/+}$ mice (Fig EV5C and D). Remarkably, a robust reduction in scoliosis was seen in *Myh3*$^{\Delta/\Delta}$ mice treated with CA3, compared with vehicle-treated mice (Fig 6W and X). Upon quantification of the Cobb angle which measures the severity of scoliosis (Wang *et al*, 2018), a significant reduction was seen in CA3-treated *Myh3*$^{\Delta/\Delta}$ mice (Fig 6Y).

To test whether administration of CA3 starting in adult life had similar effects on the musculoskeletal abnormalities exhibited by

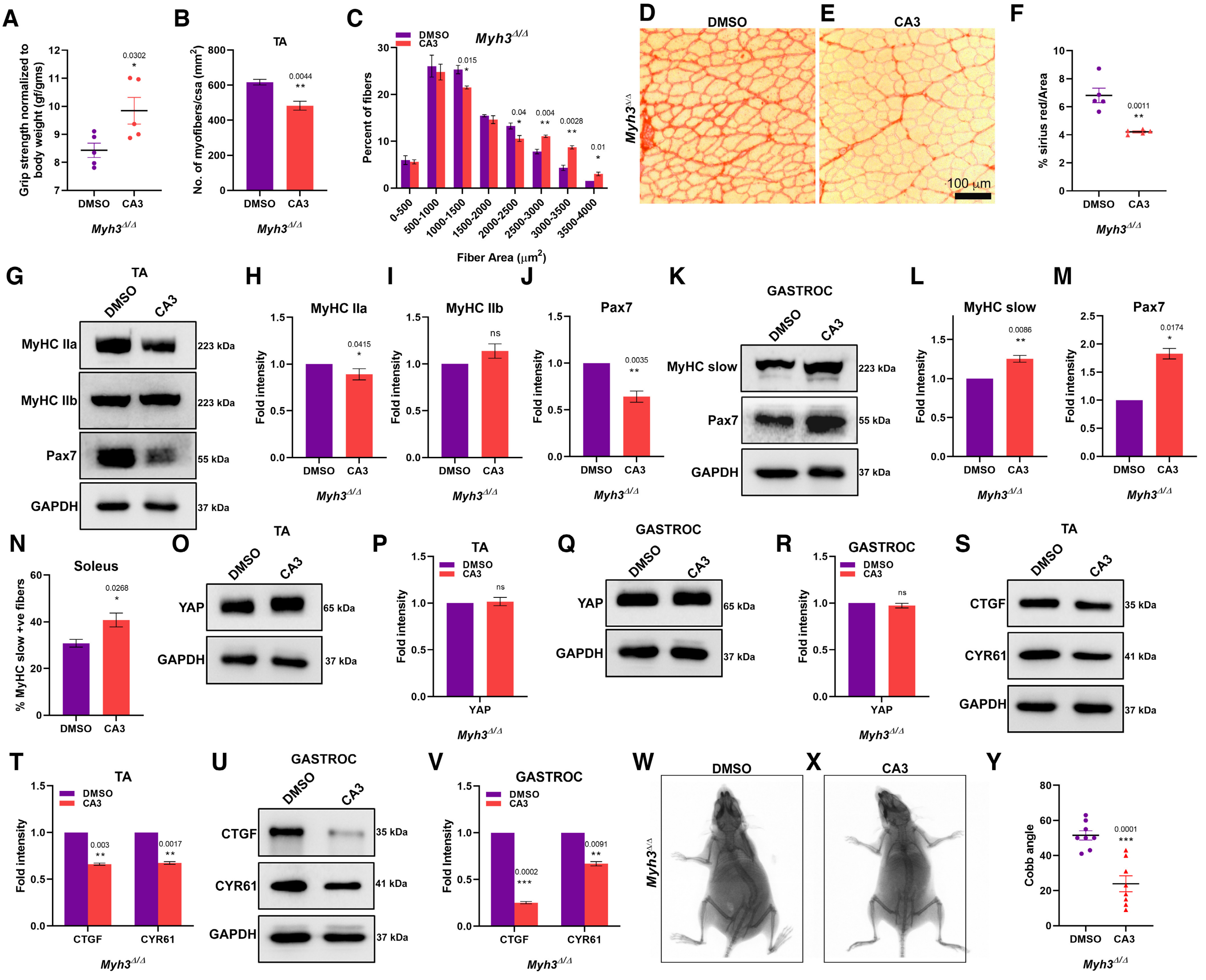

Figure 6. Inhibition of YAP activation by CA3 rescues the musculoskeletal defects in *Myh3*$^{\Delta/\Delta}$ mice.

A Quantification of grip strength normalized to body weight of *Myh3*$^{\Delta/\Delta}$ mice at 12–14 weeks of age, treated with DMSO (vehicle) or CA3.

B Quantification of the number of myofibers normalized to cross-sectional area (CSA) (mm^2) through the TA muscle of *Myh3*$^{\Delta/\Delta}$ mice at 12–14 weeks of age, treated with DMSO or CA3 (n = 4 mice per genotype).

C Quantification of the number of myofibers grouped according to myofiber area through the TA muscle of *Myh3*$^{\Delta/\Delta}$ mice at 12–14 weeks of age, treated with DMSO or CA3 (n = 3 mice per genotype).

D–F Representative bright-field micrographs of transverse sections through the TA muscle of *Myh3*$^{\Delta/\Delta}$ mice at 12–14 weeks of age, treated with DMSO or CA3, stained for Sirius red to label the extracellular matrix (D, E), and quantification of the percentage of Sirius red-positive area as a fraction of total area (F).

G–J Representative western blots for MyHC-IIa, -IIb, Pax7, and GAPDH using protein lysates from the TA muscle of *Myh3*$^{\Delta/\Delta}$ mice at 12–14 weeks of age, treated with DMSO or CA3 (G) and their densitometric quantification (H-J) (n = 5 mice per genotype).

K–M Representative western blots for MyHC-slow, Pax7, and GAPDH using protein lysates from the gastrocnemius muscle of *Myh3*$^{\Delta/\Delta}$ mice at 12–14 weeks of age, treated with DMSO or CA3 (K) and their densitometric quantification (L, M) (n = 4 mice per genotype).

N Quantification of the percentage of MyHC-slow-positive fibers normalized to total number of fibers through the soleus muscle of *Myh3*$^{\Delta/\Delta}$ mice at 12–14 weeks of age, treated with DMSO or CA3 (n = 4 mice per genotype).

O–R Representative western blots for total YAP and GAPDH in the TA (O), and the gastrocnemius (Q) protein lysates of *Myh3*$^{\Delta/\Delta}$ mice at 12–14 weeks of age, treated with DMSO or CA3 and their densitometric quantification (P, R) (n = 5 mice per genotype in P and n = 6 mice per genotype in R).

S–V Representative western blots for CTGF, CYR61, and GAPDH in the TA (S) and the gastrocnemius (U) protein lysates of *Myh3*$^{\Delta/\Delta}$ mice at 12–14 weeks of age, treated with DMSO or CA3 and their densitometric quantification (T, V) (n = 4 mice per genotype).

W–Y X-ray images through the dorsal region of *Myh3*$^{\Delta/\Delta}$ mice at 12–14 weeks of age, treated with DMSO (W) or CA3 (X) and quantification of the Cobb angle (Y).

Data information: Data are presented as mean ± SEM. Student's t-test was performed, with $P \leq 0.05$ considered significant. Scale bar: 100 μm (E).
Source data are available online for this figure.

© 2023 The Authors

Myh3$^{\Delta/\Delta}$ mice, we performed identical experiments as detailed above, except that CA3 dosing was initiated at 6 weeks of age (adult dosing), as opposed to the P15 time point. Thereafter, a similar dosing strategy was followed until 12 weeks of age when the mice were tested (Fig EV5E). Adult CA3 dosing regimen did not result in any significant changes in grip strength, number of myofibers per unit area (mm^2), proportion of larger myofibers, or fibrotic area (Fig EV5F–I). A slight increase in grip strength is observed in the vehicle-treated *Myh3*$^{\Delta/\Delta}$ mice (Figs 6A and EV5F) compared with untreated ones (Fig 1R), which could suggest an effect of the vehicle DMSO on muscle function. No significant change in protein levels of MyHC-IIa or MyHC-IIb in the TA or MyHC-slow in the gastrocnemius was observed upon adult CA3 dosing (Fig EV5J–M). However, Pax7 protein levels were significantly decreased in the TA but unaffected in the gastrocnemius upon adult CA3 dosing (Fig EV5J–M). Total YAP protein levels were unaffected in the TA and gastrocnemius, while the levels of the YAP target CTGF were decreased in the TA but unchanged in the gastrocnemius following adult CA3 dosing (Fig EV5N–Q). Adult CA3 administration also did not have any significant effect on the severity of scoliosis in *Myh3*$^{\Delta/\Delta}$ mice (Fig EV5R).

Thus, our results indicate that most defects seen in *Myh3*$^{\Delta/\Delta}$ mice such as reduced grip strength and fiber size, elevated muscle fibrosis, altered levels of MyHC-IIa and -slow, changed Pax7 levels and scoliosis are mediated by increased YAP pathway activation, which are rescued by treatment with the YAP inhibitor CA3 during early postnatal stages. Administration of CA3 initiated in adult stages did not have much effect on the musculoskeletal abnormalities exhibited by *Myh3*$^{\Delta/\Delta}$ mice. This indicates that the severity of the *Myh3*$^{\Delta/\Delta}$ musculoskeletal abnormalities progresses with age and early administration of CA3 possibly arrests or reverts the progression of these defects, which may not be the case in adult dosing regimen.

Discussion

In this study, we characterize the musculoskeletal defects seen in *Myh3* knockout mice during adult stages. Although missense mutations in *MYH3* lead to several monoallelic musculoskeletal diseases such as Freeman–Sheldon syndrome, Sheldon–Hall syndrome, and multiple pterygium syndrome, the disorder where *MYH3* mutations lead to partial or total loss of MyHC-embryonic function is spondylocarpotarsal synostosis (Cameron-Christie *et al*, 2018; Whittle *et al*, 2021; Kamien *et al*, 2022). Hence, the germline knockout *Myh3* mice serve as the first mammalian animal model to study *MYH3*-associated SCTS. Some of the most severe abnormalities seen in SCTS are vertebral fusion and scoliosis, which are also seen in *Myh3* knockout mice. Little is known about the muscle defects in *MYH3*-associated SCTS in human patients; however, we observe an array of muscle defects in the *Myh3* knockout mice, ranging from fiber type alterations, decrease in myofiber size, changes in satellite cell numbers, increase in fibrosis and reduced grip strength. Thus, further characterization of *MYH3*-associated SCTS patient muscle properties and function is needed to better understand this disorder.

Mutations in *FLNB*, the gene encoding the actin-binding filamin B, were the first identified cause for autosomal recessive SCTS (Krakow *et al*, 2004). Later studies confirmed this by generating a mouse knockout for *Flnb*, which exhibited similar abnormalities as seen in human patients with SCTS (Farrington-Rock *et al*, 2008). More recently, dominant and recessive *MYH3* mutations have been reported to lead to SCTS (Carapito *et al*, 2016; Cameron-Christie *et al*, 2018; Kamien *et al*, 2022). Both FLNB and MyHC-embryonic have been reported to alter transforming growth factor-β (TGF-β) signaling in SCTS, suggesting that they might function through conserved pathways (Zieba *et al*, 2016, 2017). Remarkably, our mass spectrometric analysis comparing proteins expressed in control and *Myh3*$^{\Delta/\Delta}$ embryos picked up FLNB as uniquely expressed in control skeletal muscle and not in *Myh3*$^{\Delta/\Delta}$ muscle, which was validated by western blots (Fig 4C, E, and F). This indicates that FLNB might have muscle-related functions during development, and its expression in the muscle seems to be dependent on MyHC-embryonic. We also find that talins, which link integrins to the actin cytoskeleton and regulate integrin-mediated mechanotransduction and signaling, are upregulated in *Myh3*$^{\Delta/\Delta}$ muscle (Fig 4C, E, and F). Filamins compete with talins to bind β-integrin cytoplasmic domains, to modulate integrin activation and signaling (Kiema *et al*, 2006). Thus, *MYH3*-associated SCTS might be closely linked to FLNB and Talin expression, which in turn mediate integrin activation and function, which would be of interest to be explored further using patient samples.

YAP signaling has been studied in the context of the mammalian skeletal muscle, where it has been proposed to have multiple functions. In a synergist ablation model where the soleus and distal gastrocnemius muscles were surgically removed to induce mechanical overload on the plantaris muscle in mice, YAP levels were shown to increase, suggesting that YAP responds to mechanical cues in the skeletal muscle (Goodman *et al*, 2015). Overexpression of YAP by electroporation of a *YAP* plasmid into the TA muscle of mice led to muscle hypertrophy, independent of the mammalian target of rapamycin complex 1 (Goodman *et al*, 2015). In another study where adeno-associated viral vector-mediated *YAP* shRNA was delivered into the mouse TA muscle to knock down *YAP* expression, a decrease in muscle mass and myofiber cross-sectional area was seen (Watt *et al*, 2015). In the same study, adeno-associated viral vector-mediated overexpression of *YAP* in the skeletal muscle led to muscle hypertrophy characterized by increased muscle mass and myofiber cross-sectional area; however, YAP overexpression at greater levels led to initial muscle hypertrophy followed by muscle fiber degeneration (Watt *et al*, 2015). Expression of a constitutively active version of YAP in the mouse skeletal muscle led to reduction in adult body weight, decrease in muscle fiber cross-sectional area, muscle fiber atrophy, necrosis, myopathy, and kyphosis (Judson *et al*, 2013). Thus, the effects of YAP on the adult skeletal muscle seem to be variable, dependent on the method, time point and level of overexpression. The *Myh3*$^{\Delta/\Delta}$ musculoskeletal defects that we report show similarities to the transgene-mediated expression of constitutively active YAP in the mouse skeletal muscle, especially with respect to the body weight, muscle fiber size and skeletal defects (Judson *et al*, 2013). This is also an independent confirmation that activation of YAP signaling underlies the musculoskeletal defects seen upon loss of MyHC-embryonic function. YAP signaling could be a conserved dysregulated target for multiple myosin isoforms, since MyHC-perinatal depletion also results in altered YAP levels (Fig 4J and K). This is further confirmed by altered YAP signaling observed upon treatment of myogenic cells with contractility inhibitors such as para-aminoblebbistatin (Fig EV4A–D).

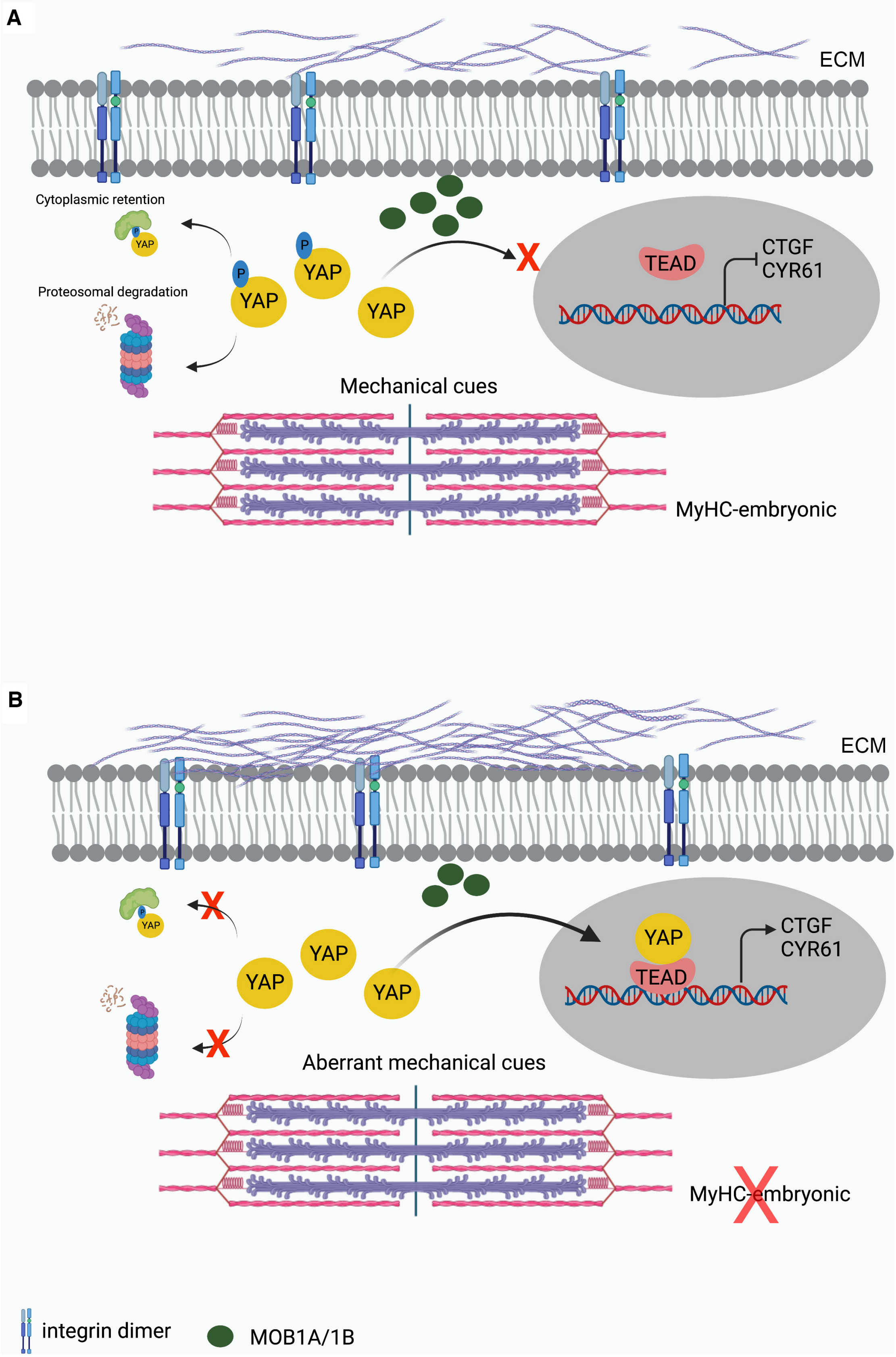

Figure 7.

 © 2023 The Authors

Figure 7. Model.

A Presence of MyHC-embryonic in the developing skeletal muscle sarcomeres results in normal contractile properties, mechanical cues, and integrin signaling, leading to activation of the upstream kinases in the Hippo signaling pathway which phosphorylate YAP aided by adaptors such as MOB1A/1B, causing cytoplasmic retention or proteasomal degradation of YAP. Reduced levels of nuclear YAP prevent the activation of YAP target genes such as CTGF and CYR61 and reduced fibrosis.

B Absence of MyHC-embryonic in the sarcomeres leads to aberrant contractility, mechanical cues and integrin signaling, reduction in levels of Hippo pathway adaptors such as MOB1A/1B resulting in lack of YAP phosphorylation leading to its stabilization, nuclear entry, binding to TEAD, activation of downstream targets such as CTGF and CYR61 and increased fibrosis.

The expression of the mammalian skeletal muscle-specific developmental MyHCs- MyHC-embryonic and MyHC-perinatal during adult life are mostly restricted to damaged, diseased and regenerating skeletal muscle fibers, except in the case of some muscles such as the masseter where MyHC-embryonic is expressed in adult life (d'Albis *et al*, 1986; Butler-Browne *et al*, 1988). Although there are no reports on normal adult limb muscles expressing MyHC-embryonic, it is likely that transient expression of MyHC-embryonic occurs during homeostasis. This is because satellite cells have been shown to contribute to adult myofibers in the absence of injury, and transient expression of MyHC-embryonic is seen during muscle stem cell differentiation, both during development and regeneration (Keefe *et al*, 2015). Interestingly, three of the muscle phenotypes seem to change with age in adult *Myh3*$^{\Delta/\Delta}$ mice. First, we reported that MyHC-slow levels and the number of MyHC-slow+ fibers increase during development and perinatal stages in mice lacking MyHC-embryonic function (Agarwal *et al*, 2020). However, muscles of 8–10-week-old *Myh3*$^{\Delta/\Delta}$ mice exhibit a severe reduction in MyHC-slow levels and MyHC-slow+ fibers, which becomes even more reduced by 6 months of age (Fig 2M–P and V–X). Second, the number of myofibers in the TA showed no difference between control and *Myh3*$^{\Delta/\Delta}$ mice at 8–10 weeks, but was significantly increased in *Myh3*$^{\Delta/\Delta}$ mice at 6 months of age (Fig 1G and H). Third, the levels of Pax7 and the number of Pax7+ cells were increased in the muscles of 8–10-week-old *Myh3*$^{\Delta/\Delta}$ mice, whereas these were decreased by 6 months of age (Fig 3A–I). Using isolated single muscle fiber culture experiments, we show that Pax7+ satellite cells exhibit increased activation, which likely explains the elevated satellite cell numbers at early time points and an exhaustion of the satellite cell pool with age (Fig 3G, I, L, and N–P). Thus, loss of MyHC-embryonic in adult muscle seems to alter certain muscle characteristics with age, hinting at possible adult expression and function of MyHC-embryonic, although this might also result from muscle adaptation-related effects.

We find that loss of MyHC-embryonic during embryonic development leads to altered expression of different myosin proteins (Fig 4D), which probably results in reduced myosin ATPase activity that we observe (Fig EV1M). We observe increased expression of the two other developmental MyHCs, MyHC-perinatal, and MyHC-slow, encoded by the *Myh8* and *Myh7* genes, respectively, which are co-expressed with MyHC-embryonic during developmental stages (Fig 4D). We had reported similar results previously and these results validate our previous findings (Agarwal *et al*, 2020). This is along expected lines, since loss of one MyHC is likely to be compensated by others expressed at similar stages, and has been reported in other MyHC knockouts such as the MyHC-IIx knockout (Sartorius *et al*, 1998). Knockdown of *Myh8* has similar effects on YAP and phospho-YAP levels, indicating conserved signaling and mechanotransduction pathways involving developmental MyHCs (Fig 4J and K). We also observe a slight increase in nonmuscle myosin heavy chain-IIb (encoded by the *Myh10* gene; Fig 4D). Interestingly, nonmuscle myosin heavy chain-IIb is thought to be important in early stages of sarcomere formation where it is replaced at later stages by muscle myosin isoforms (White *et al*, 2014). These results might thus point to sarcomere formation abnormalities in *Myh3*$^{\Delta/\Delta}$ mice. We also observe reduction in protein levels of two myosin essential light chain isoforms, namely myosin light chain-1/3 fast (encoded by the *Myl1* gene) and myosin light chain-embryonic/atrial fast (encoded by the *Myl4* gene; Fig 4D). These are light chain isoforms known to be specifically expressed during embryonic and fetal stages of development and their downregulation could indicate that these light chain isoforms bind to MyHC-embryonic specifically to regulate its function (Lyons *et al*, 1990; Schiaffino *et al*, 2015). Inhibitors of cell contractility and myosin function have effects on YAP signaling, validating the role of myosins in mechanotransduction (Fig EV4A–D).

As with perinatal stages, the consequence of loss of MyHC-embryonic seems to vary across different muscles. Most notable is that Pax7 levels and Pax7+ cell numbers are increased in the TA of *Myh3*$^{\Delta/\Delta}$ mice at 8–10 weeks and decrease at 6 months of age (Fig 3A–G, H, and K). Interestingly, at P30, an earlier time point, Pax7+ cell numbers in the TA are increased in *Myh3*$^{\Delta/\Delta}$ mice (Fig EV3A–C), indicating that the increase in Pax7+ cells in *Myh3*$^{\Delta/\Delta}$ mice happens postnatally, which starts decreasing post-8–10 weeks. In the case of the gastrocnemius, Pax7 levels exhibit a consistent decrease in *Myh3*$^{\Delta/\Delta}$ mice at the 8–10-week, and 6-month time points (Fig EV3D–F). It is likely that fiber type composition, muscle function and activity levels, metabolic properties, and related aspects play important roles in determining satellite cell numbers, which could explain the observed differences in distinct muscles.

Model and conclusions

Based on our findings, we propose that MyHC-embryonic in the developing skeletal muscle sarcomeres is required for normal contractility, mechanical cues, and integrin signaling. This leads to activation of the upstream kinases in the Hippo signaling pathway which phosphorylate YAP with the help of adaptors such as MOB1A/1B. Phosphorylated YAP is retained in the cytoplasm or degraded by the proteasome, resulting in reduced levels of nuclear YAP, preventing the activation of YAP target genes such as CTGF and CYR61 and reduced fibrosis (Fig 7A). In *Myh3*$^{\Delta/\Delta}$ mice, loss of MyHC-embryonic in the sarcomeres leads to aberrant contractility, mechanical cues, and integrin signaling. This in turn causes reduction in levels of the Hippo pathway adaptors such as MOB1A/1B preventing YAP phosphorylation leading to its stabilization, nuclear entry, binding to TEAD, activation of downstream targets such as

© 2023 The Authors

CTGF and CYR61 and increased fibrosis (Fig 7B). Inhibiting the YAP signaling pathway by CA3 during early postnatal stages reduces YAP target gene activation and normalizes most of the musculoskeletal defects exhibited upon loss of MyHC-embryonic function. Thus, YAP signaling is a crucial therapeutic target in *MYH3*-associated musculoskeletal diseases such as spondylocarpotarsal synostosis.

Materials and Methods

Mice

Myh3 knockout mice (*Myh3*$^{\Delta/\Delta}$) were generated and genotyped as described previously (Agarwal *et al*, 2020). Mice were euthanized at selected time points (E16.5, P0, 4 weeks, 8–10 weeks, 12–14 weeks [4 months] and 6 months), as required for the study. All muscles of the hind limb were used at E16.5 and P0, whereas specific muscles (TA, gastrocnemius and soleus) were used at all other stages. Mice were maintained and all animal experiments performed according to the protocols approved by the institutional animal ethics committee (protocol numbers: RCB/IAEC/2016/005 and RCB/IAEC/2019/056).

DMSO (Sigma; D8418) or CA3 (Selleck chemicals; CIL56) resuspended in DMSO at 25 mg/ml, was administered intraperitoneally to control *Myh3*$^{+/+}$ or *Myh3*$^{\Delta/\Delta}$ mice at a dose of 1 mg/kg body weight, starting at 15-day postbirth, twice a week, for 6 weeks. To study its effect during adult life, 6–7-week-old mice were administered CA3. The treated animals were allowed to age until 12–14 weeks. Post-treatment, x-ray images of the mice were captured using an *in vivo* imaging system (Lago X, Spectral instruments imaging) to measure the Cobb angle. To determine scoliosis, the Cobb angle was measured manually using the vertebrae tilt method (Wang *et al*, 2018; Chen *et al*, 2021). The hind limb muscles of the treated mice were harvested following euthanasia and used for further experiments. MicroCT imaging of control and *Myh3*$^{\Delta/\Delta}$ mice was carried out using an *in vivo* imaging system (Perkin Elmer; Quantum GX2 microCT). Fore and hind limb grip measurements were recorded using a grip strength meter (GT3, Panlab, Harvard Apparatus). For the rotarod test, mice were trained on a standard accelerating rotarod (Harvard Apparatus) for 3 days. In the final experiment, each mouse was placed on the rotating drum of the apparatus, rotating at a ramp-up speed of 0–50 rpm for 50 s. The falling mice land on a platform connected to a timer which records the duration spent on the rotating drum. Results were represented as three technical readings per mouse. For the treadmill assay, mice were acclimatized to running on the treadmill apparatus (Columbus Instruments) at a speed of 10 m/min for 5 min, for 3 days. For the final experiment, each mouse was allowed to run on the treadmill at 10 m/min for 5 min, following which the speed was increased by 2 m/min every 2 min until exhaustion, when the duration and distance were noted. Male mice were used for body weight, muscle weight, grip strength, rotarod, and treadmill assays, where there are known differences between sexes. For all other parameters and analyses, gender segregation was not done. Control *Myh3*$^{+/+}$ and *Myh3*$^{\Delta/\Delta}$ littermates were randomly selected for treatments. For grip strength assay, the investigators were blind to the treatment given.

Immunofluorescence, histology, microscopy, and image analysis

The muscle tissues were harvested and embedded in cryomatrix (Thermo Scientific; 6769006) cryo-sectioning media and flash-frozen in cooled 2-methyl butane (Sigma; M32631). The embedded tissue was sectioned at 10 μm thickness using a cryomicrotome (Thermo Scientific; Microm HM 550) and adjacent sections collected on charged glass slides (Avantor; 631-0108). Adjacent sections were processed for the detection of adult myosin heavy chain isoforms (MyHC-IIa, -IIb, -IIx), laminin, and Pax7, respectively. To stain for adult MyHC isoforms, sections were blocked with 10% goat serum (BioAbChem, 72-0480) and 1% Tergitol solution (Sigma; NP-40) in ice-cold phosphate-buffered saline (PBS), after acetone fixation (MyHC-IIa, -IIx) or no fixation (MyHC-IIb), incubated with the cocktail of adult MyHC isoform-specific sera and laminin antibody in appropriate dilutions in 10% goat serum-PBS, at 4°C overnight. Slides were rinsed with ice-cold PBS twice and 0.5% PBS-Triton X-100 (PBST) once, incubated in biotin-conjugated secondary antibody for amplification at 4°C overnight, followed by incubation in streptavidin-coupled fluorophore for 2 h at 4°C. The sections were then rinsed in PBS, stained with Hoechst (ThermoFisher Scientific; H3570), fixed in 4% paraformaldehyde (PFA), and mounted using Fluoromount-G (Southern Biotech; 0100-01). To stain for Pax7, sections were fixed with 4% PFA and the antigens were retrieved using an antigen retriever (2100 PickCell Retriever; Aptum Biologics) in citrate buffer (1.8 mM citric acid and 8.2 mM sodium citrate in water). The sections were blocked using TNB blocking buffer (Perkin Elmer; FP1012) for 1 h and incubated with Pax7 sera in appropriate concentration diluted in TNB buffer. The signals were amplified using vectastain (Vector Laboratories; PK-6100) and TSA reagent kits (Perkin Elmer; NEL741B001KT, NEL744B001KT). Details of all antibodies used are provided in Appendix Table S1. The sections were imaged using an Olympus fluorescence microscope (Olympus BX63F), with a Hamamatsu ORCA-Flash 4.0 camera. Each image was maximally projected, and the number of MyHC+ fibers and fiber area were quantified using SMASH (Semiautomatic muscle analysis using segmentation of histology software; Smith & Barton, 2014). The cross-sectional area was measured using CellSens Dimension 1.16 software and statistically analyzed (GraphPad Prism 5).

Single fibers were isolated from the EDL muscle as described previously (Agarwal *et al*, 2022) of 8–10-week-old *Myh3*$^{+/+}$ and *Myh3*$^{\Delta/\Delta}$ mice and fixed in 4% PFA for 15 min. The fibers were blocked in 5% goat serum in 1x PBS containing 0.1% Triton X-100 (MP Biomedicals; 194854) for 1 h and incubated in primary antibodies overnight at 4°C. The fibers were then rinsed in PBS, incubated with secondary antibodies for 2 h at room temperature, rinsed in PBS, and mounted in fluoromount-G. The fibers were imaged using a Nikon Eclipse Ti microscope with a DS-Qi2 camera.

To study fibrosis, muscle sections were stained with Sirius red. Briefly, muscle sections were fixed for 1 h in Bouin's fixative (75% saturated picric acid, 25% Formaldehyde, and 5% Glacial acetic acid) at 56°C, washed with tap water, and stained with Picro-Sirius red (Sigma; 365548) for 1 h, followed by washing with 0.5% acetic acid. The sections were dehydrated in 50, 70, and 100% ethanol, equilibrated with xylene, and mounted in DPX (Sigma; 06522). The sections were imaged using a Nikon Eclipse Ti microscope with a DS-Fi2 camera.

© 2023 The Authors

To document spinal fusion, whole-mount skeletal staining of bone and cartilage was done using Alizarin red and Alcian blue as described (Rigueur & Lyons, 2014). Briefly, 4-month-old *Myh3*$^{+/+}$ and *Myh3*$^{\Delta/\Delta}$ mice were euthanized, deskinned and all organs removed. The samples were dehydrated in 95% ethanol and incubated in acetone to remove fats and permeabilize. This was followed by incubation in Alcian blue (Loba Chemie Pvt. Ltd; 0083000005), destaining in 70% ethanol and 95% ethanol. The specimens were cleared in 1% potassium hydroxide, transferred to Alizarin red (Loba Chemie Pvt. Ltd; 0086000025) prepared in potassium hydroxide and destained in 1% potassium hydroxide. The samples were stored in 100% glycerol. Imaging of cervical, thoracic, and lumbar vertebrae was performed on a bright-field stereozoom microscope (Nikon; SMZ 745T). The percentage of vertebral fusion was calculated by manually counting the number of fused vertebrae as a fraction of vertebrae for that group.

Western blotting

Hind limb muscles were harvested from *Myh3*$^{+/+}$ and *Myh3*$^{\Delta/\Delta}$ embryos or mice and flash-frozen in liquid nitrogen. For protein lysate preparation, the muscles were transferred to ice-cold radioimmunoprecipitation assay (RIPA) buffer (Sigma; R0278-500ml) containing protease/phosphatase inhibitor cocktail (Cell Signaling Technology; 5872S). The muscles were homogenized, vortexed intermittently for 30 min on ice, followed by centrifugation at 16,000 *g* for 15 min at 4°C. The supernatant was collected and protein samples were quantified using the Pierce BCA Protein Assay kit (Thermo Scientific; 23225). Protein samples were separated on SDS–PAGE followed by western blots using standard protocols. Blots were blocked in 5% BSA (phospho-antibodies) or 5% milk, incubated with primary antibodies at 4°C, overnight, and subsequently with HRP-conjugated secondary antibodies at room temperature for 2 h, followed by detection using the HRP substrate (Millipore; WBLUF0100). The blots were imaged on an ImageQuant LAS 4000 (GE) and densitometric quantification was performed using ImageJ, where protein levels were normalized to GAPDH levels.

The nuclear and cytoplasmic protein fractions were prepared from the hind limb muscles of *Myh3*$^{+/+}$ and *Myh3*$^{\Delta/\Delta}$ mice according to published protocols (Dimauro *et al*, 2012). Briefly, tissues were homogenized in ice-cold STM buffer (250 mM sucrose, 50 mM Tris–HCl pH 7.4, 5 mM magnesium chloride), vortexed, incubated on ice for 30 min, and centrifuged at 800 *g* for 15 min. The pellet obtained was processed for nuclear fraction and the supernatant for cytoplasmic fraction. The pellet was resuspended in STM buffer, vortexed, and centrifuged again at 800 *g* to get rid of debris. Finally, the pellet was resuspended in NET buffer (20 mM HEPES pH 7.9, 1.5 mM magnesium chloride, 0.5 M sodium chloride, 0.2 mM EDTA, 20% glycerol, and 1% triton x-100), vortexed, and incubated on ice for 30 min. The nuclei were lysed by sonication and centrifuged to obtain pure nuclear lysate fraction. The supernatant for the cytoplasmic fraction was centrifuged twice at 11,000 *g* for 10 min and the pellet was discarded. The proteins in the supernatant were precipitated using 100% acetone and resuspended in STM buffer after centrifugation. The quantity of protein was estimated as mentioned above, and western blots were performed. H3A and GAPDH were used to normalize the quantity of proteins in the nuclear and cytoplasmic fractions, respectively. Details of all antibodies used are provided in Appendix Table S1.

ATPase assay

Actin was isolated from mouse hind limb skeletal muscle using standard protocol (Racusen & Thompson, 1997; Das *et al*, 2019). Myosin heavy chain proteins were isolated in 300 µl of myosin extraction buffer (1.0 M KCl, 0.15 M potassium phosphate pH 6.8, 10 mM sodium pyrophosphate, 5 mM $MgCl_2$, 0.5 mM EGTA, 8 mM DTT and 1x protease inhibitor cocktail). The extracted myosin was loaded onto a 100 kDa cut-off column and washed in myosin storage buffer (0.5 M KCl, 20 mM MOPS pH 7.0, 2 mM $MgCl_2$, and 8 mM DTT) with 5 mM ATP thrice followed by thrice without ATP. The extracted myosin was quantified using BCA kit, and ATPase hydrolysis assay was performed using the EnzCheck Phosphate Assay kit (E6646 ThermoFisher Scientific) according to the manufacturer's protocol. The absorbance was measured at 360 nm at 10-s intervals for 1 h.

RNA isolation, cDNA synthesis, and quantitative PCR (qPCR)

The TA muscle was harvested and flash-frozen in liquid nitrogen from 12 to 14-week-old *Myh3*$^{+/+}$ and *Myh3*$^{\Delta/\Delta}$ mice. Muscle samples were homogenized, RNA isolated using the RNeasy Lipid Tissue Mini Kit (Qiagen; 74804), and cDNA synthesized using SuperScript III reverse transcriptase (Invitrogen; 18080-044) and oligo (dT) (Invitrogen; 58862) as per manufacturer's instructions. qPCR was performed with TB Green Premix Ex Taq (Takara; RR420A) on an ABI 7500 Fast Real Time PCR system (Applied Biosystems) and normalized to *Gapdh* transcript levels. Details of all primers used are provided in Appendix Table S2. A minimum of three biological replicates were used for the expression analysis.

Mass spectrometry

Timed matings were set up and *Myh3*$^{+/+}$ and *Myh3*$^{\Delta/\Delta}$ embryos were collected at E16.5, hind limb muscles isolated, homogenized, and sonicated in RIPA buffer followed by centrifugation at 13,000 *g* at 4°C for 10 min. The supernatant was subjected to acetone (Sigma; 179124) precipitation, incubated first at −20°C for 10 min, and then at −80°C for 30 min. After centrifugation at 3,500 *g* for 45 min at 4°C, the pellet was washed twice with acetone and dissolved in 8 M urea (Sigma; U5378). Following quantification by BCA assay, 100 µg of protein was processed further. Briefly, after adding Dithiothreitol (Sigma; 53819), the samples were incubated for 1 h at 56°C, which was followed by incubation in dark at room temperature for 30 min, with the addition of cysteine blocking agent (IAA; Sigma; 15161) to the samples. Samples were then digested with trypsin (Thermo Scientific; 90057) at 37°C overnight. Peptide extraction was performed by ultrasonication for 20 min in an extraction solution (50% Acetonitrile, 0.1% Formic acid) and the extract was dried in a speed vacuum concentrator at 25°C. The samples were then desalted using Pierce C18 Zip-tips (Thermo Scientific; 87782) and run on an LC–MS/MS mass spectrometer (Triple TOF 5600 SCIEX).

Mascot generic files (mgf) were generated by using the raw files of the MS–MS run, which were then conferred protein identification after database searches using Search GUI. Sample peptides were

© 2023 The Authors

scored in the form of emPAI (exponentially modified Protein Abundance Index) using MASCOT software, run with a false discovery rate of 1%.

Cell culture and luciferase assay

C2C12 mouse myoblasts (ATCC; CRL-1722) were maintained according to ATCC guidelines in culture media containing DMEM-Dulbecco's Modified Eagle Medium (Gibco; 12800-017) supplemented with 10% (v/v) fetal bovine serum (Sigma; F2442) and 2% penicillin–streptomycin (Gibco; 15140122).

For luciferase assay, ~ 30,000 C2C12 cells were seeded in each well of a 24-well plate (Nunc; 142485) and the cells were reverse transfected with control or *Myh3* siRNA using Lipofectamine RNAiMAX (Thermo Fisher; 13778150). After 24 h, growth media containing siRNA was removed and the cells were transfected with an *8XGTIIC-luciferase* (Addgene; 34615) reporter plasmid (1 µg/well; Dupont *et al*, 2011) and *pHRL-CMV renilla luciferase* (Promega; E6271) plasmid (20 pg/well) using Lipofectamine 2000 (Thermo Fisher; 11668019). Post-24 h, the media was replaced with fresh media for 24 h, thereafter replaced with differentiation media (DMEM containing 2% [v/v] horse serum [BioAbChem; 72-0460] and 1% penicillin–streptomycin). Luciferase assay was performed after 24 h of culture in differentiation media, using a luminometer (Promega) with the Dual-Glo Luciferase assay system (Promega; E2920).

For protein lysates, ~ 1,20,000 cells were seeded in each well of a 6-well plate and transfected with control or *Myh3* siRNA using Lipofectamine RNAiMAX as described above. The cells were shifted to complete media after 24 h, cultured for another 24 h, following which the complete media was changed to differentiation media. The cells were allowed to differentiate in this media for 24 h, harvested, protein lysates prepared in RIPA buffer and processed for western blots.

For cell contractility assay, ~ 50,000 C2C12 cells were reverse transfected with control or *Myh3* siRNA in each well of a 24-well plate. After 24 h, the cells were trypsinized and mixed with a collagen matrix as per manufacturer's protocol (Cell Biolabs, Cell contraction assay, Cat No. CBA-201). The cells were supplemented with complete media, which was replaced with differentiation media after 24 h. The cells were cultured in differentiation media for 48 h, and the collagen was gently released by a pipette tip and monitored for 24 h.

The effect of cell contraction modulators on YAP signaling in C2C12 cells was studied by plating ~ 30,000 cells in each well of a 24-well plate. The cells were cultured in complete media for 2 days, followed by replacement with differentiation media supplemented with the contraction modulators. The contraction modulators used were Para-aminoblebbistatin (10 µM; Cayman Chemicals, Cat. No. 22699) and 2,3 Butanedione monoxime (BDM; 10 mM; Cell Biolabs, Cat. No. 20105). Cells were treated with the contraction modulators or DMSO for 24 h and lysates prepared for western blots as described above.

Statistical analysis

Experimental data were analyzed using GraphPad Prism 8 software (GraphPad Software Inc., CA, USA) and plotted as mean ± standard error of the mean. Parametric, two-tailed unpaired *t*-test was used to test the probability of significant differences, and *P*-values ≤ 0.05 were considered significant (asterisk). All the animal studies were conducted using at least three biological replicates. Control $Myh3^{+/+}$ or $Myh3^{\Delta/\Delta}$ littermates were randomly selected for treatments. For grip strength tests, the investigator was blind to the treatments given.

The paper explained

Problem
Mutations in the *MYH3* gene encoding the myosin heavy chain-embryonic (MyHC-embryonic) protein lead to several human congenital musculoskeletal diseases such as Freeman–Sheldon syndrome, Sheldon–Hall syndrome, multiple pterygium syndrome and spondylocarpotarsal synostosis (SCTS). A clear characterization of the abnormalities seen in such patients is lacking. To develop treatments for such diseases, it is vital to decipher the normal functions of MyHC-embryonic and identify target signaling pathways by generating mammalian animal models.

Results
We report the first mouse model for the *MYH3*-loss of function associated SCTS, which recapitulates characteristics of the human syndrome. *Myh3*-loss of function mice exhibit several structural and functional abnormalities of the musculoskeletal system. In addition, *Myh3* knockout mice exhibit altered mechanical signals, leading to elevated signaling through the mechanoresponsive Yes-associated protein (YAP) transcriptional regulator.

Impact
Our results identify MyHC-embryonic as a critical regulator of mechanical cues and the YAP signaling pathway in the skeletal muscle. An inhibitor of YAP signaling rescues most musculoskeletal defects exhibited by *Myh3* knockout mice, indicating that YAP could be a promising therapeutic target to treat SCTS and other *MYH3*-associated congenital musculoskeletal diseases.

Data availability

This manuscript does not have data that need to be deposited in a public database.

Expanded View for this article is available online.

Acknowledgements
This work was funded by a grant from the Department of Biotechnology (grant number BT/PR32938/BRB/10/1851/2020) and an Intermediate Fellowship from the Wellcome Trust/DBT India Alliance (IA/I/13/1/500872) to SJM. We also acknowledge funding from the Regional Centre for Biotechnology (RCB). AB is currently funded by a senior research fellowship from the Indian Council of Medical Research (ICMR) and previously by the University Grants Commission (UGC), JSh by a Department of Science and Technology INSPIRE junior research fellowship, MK by an ICMR junior research fellowship, and BK funded by a national postdoctoral fellowship from the Science and Engineering Research Board. We acknowledge the small animal facility (SAF) for the animal work and imaging facility at RCB for the

 © 2023 The Authors

microscopy. We thank Sebanta Pokhrel for help with mouse breeding and maintenance, Dr. Pankaj Kumar for help with the grip strength assay, and Dr. P. Nagarajan at the National Institute of Immunology (NII) for help with the whole animal imaging. The vertebral column schematic was designed using Freepik.com. We also acknowledge past and present members of SJM laboratory for valuable suggestions and inputs. The authors acknowledge the support of DBT e-Library Consortium (DeLCON) for providing access to e-resources.

Author contributions

Anushree Bharadwaj: Conceptualization; data curation; formal analysis; validation; investigation; visualization; methodology; writing – review and editing. **Jaydeep Sharma:** Conceptualization; data curation; formal analysis; validation; investigation; visualization; methodology. **Jagriti Singh:** Data curation; formal analysis; validation; investigation; visualization. **Mahima Kumari:** Data curation; formal analysis; validation; investigation; visualization; methodology. **Tanushri Dargar:** Data curation; formal analysis; validation; investigation; visualization. **Bhargab Kalita:** Data curation; formal analysis; validation; investigation. **Sam J Mathew:** Conceptualization; resources; data curation; formal analysis; supervision; funding acquisition; investigation; methodology; writing – original draft; project administration; writing – review and editing.

Disclosure and competing interests statement

The authors declare that they have no conflict of interest.

For more information

https://www.omim.org/entry/272460.

References

Agarwal M, Sharma A, Kumar P, Kumar A, Bharadwaj A, Saini M, Kardon G, Mathew SJ (2020) Myosin heavy chain-embryonic regulates skeletal muscle differentiation during mammalian development. *Development* 147: dev184507

Agarwal M, Bharadwaj A, Mathew SJ (2022) TLE4 regulates muscle stem cell quiescence and skeletal muscle differentiation. *J Cell Sci* 135: jcs256008

Bae SJ, Kim M, Kim SH, Kwon YE, Lee JH, Kim J, Chung CH, Lee WJ, Seol JH (2015) NEDD4 controls intestinal stem cell homeostasis by regulating the Hippo signalling pathway. *Nat Commun* 6: 6314

Basu S, Totty NF, Irwin MS, Sudol M, Downward J (2003) Akt phosphorylates the Yes-associated protein, YAP, to induce interaction with 14-3-3 and attenuation of p73-mediated apoptosis. *Mol Cell* 11: 11–23

Beck AE, McMillin MJ, Gildersleeve HI, Kezele PR, Shively KM, Carey JC, Regnier M, Bamshad MJ (2013) Spectrum of mutations that cause distal arthrogryposis types 1 and 2B. *Am J Med Genet A* 161A: 550–555

Beck AE, McMillin MJ, Gildersleeve HI, Shively KM, Tang A, Bamshad MJ (2014) Genotype-phenotype relationships in Freeman–Sheldon syndrome. *Am J Med Genet A* 164A: 2808–2813

Boppart MD, Mahmassani ZS (2019) Integrin signaling: linking mechanical stimulation to skeletal muscle hypertrophy. *Am J Physiol Cell Physiol* 317: C629–C641

Butler-Browne GS, Eriksson PO, Laurent C, Thornell LE (1988) Adult human masseter muscle fibers express myosin isozymes characteristic of development. *Muscle Nerve* 11: 610–620

Calderwood DA, Huttenlocher A, Kiosses WB, Rose DM, Woodside DG, Schwartz MA, Ginsberg MH (2001) Increased filamin binding to beta-integrin cytoplasmic domains inhibits cell migration. *Nat Cell Biol* 3: 1060–1068

Cameron-Christie SR, Wells CF, Simon M, Wessels M, Tang CZN, Wei W, Takei R, Aarts-Tesselaar C, Sandaradura S, Sillence DO *et al* (2018) Recessive spondylocarpotarsal synostosis syndrome due to compound heterozygosity for variants in MYH3. *Am J Hum Genet* 102: 1115–1125

Carapito R, Goldenberg A, Paul N, Pichot A, David A, Hamel A, Dumant-Forest C, Leroux J, Ory B, Isidor B *et al* (2016) Protein-altering MYH3 variants are associated with a spectrum of phenotypes extending to spondylocarpotarsal synostosis syndrome. *Eur J Hum Genet* 24: 1746–1751

Chan SW, Lim CJ, Chong YF, Pobbati AV, Huang C, Hong W (2011) Hippo pathway-independent restriction of TAZ and YAP by angiomotin. *J Biol Chem* 286: 7018–7026

Chen ZY, Luk KDK, Song YQ, Gao B, Cheung JPY (2021) Proper positioning of mice for Cobb angle radiographic measurements. *BMC Musculoskelet Disord* 22: 72

Chong JX, Burrage LC, Beck AE, Marvin CT, McMillin MJ, Shively KM, Harrell TM, Buckingham KJ, Bacino CA, Jain M *et al* (2015) Autosomal-dominant multiple pterygium syndrome is caused by mutations in MYH3. *Am J Hum Genet* 96: 841–849

Conti FJ, Felder A, Monkley S, Schwander M, Wood MR, Lieber R, Critchley D, Muller U (2008) Progressive myopathy and defects in the maintenance of myotendinous junctions in mice that lack talin 1 in skeletal muscle. *Development* 135: 2043–2053

Conti FJ, Monkley SJ, Wood MR, Critchley DR, Muller U (2009) Talin 1 and 2 are required for myoblast fusion, sarcomere assembly and the maintenance of myotendinous junctions. *Development* 136: 3597–3606

d'Albis A, Janmot C, Bechet JJ (1986) Comparison of myosins from the masseter muscle of adult rat, mouse and Guinea-pig. Persistence of neonatal-type isoforms in the murine muscle. *Eur J Biochem* 156: 291–296

Das S, Kumar P, Verma A, Maiti TK, Mathew SJ (2019) Myosin heavy chain mutations that cause Freeman-Sheldon syndrome lead to muscle structural and functional defects in Drosophila. *Dev Biol* 449: 90–98

Dimauro I, Pearson T, Caporossi D, Jackson MJ (2012) A simple protocol for the subcellular fractionation of skeletal muscle cells and tissue. *BMC Res Notes* 5: 513

Dupont S, Morsut L, Aragona M, Enzo E, Giulitti S, Cordenonsi M, Zanconato F, Le Digabel J, Forcato M, Bicciato S *et al* (2011) Role of YAP/TAZ in mechanotransduction. *Nature* 474: 179–183

Elbediwy A, Vincent-Mistiaen ZI, Spencer-Dene B, Stone RK, Boeing S, Wculek SK, Cordero J, Tan EH, Ridgway R, Brunton VG *et al* (2016) Integrin signalling regulates YAP and TAZ to control skin homeostasis. *Development* 143: 1674–1687

Farrington-Rock C, Kirilova V, Dillard-Telm L, Borowsky AD, Chalk S, Rock MJ, Cohn DH, Krakow D (2008) Disruption of the Flnb gene in mice phenocopies the human disease spondylocarpotarsal synostosis syndrome. *Hum Mol Genet* 17: 631–641

Goodman CA, Dietz JM, Jacobs BL, McNally RM, You JS, Hornberger TA (2015) Yes-Associated Protein is up-regulated by mechanical overload and is sufficient to induce skeletal muscle hypertrophy. *FEBS Lett* 589: 1491–1497

Hayashi YK, Chou FL, Engvall E, Ogawa M, Matsuda C, Hirabayashi S, Yokochi K, Ziober BL, Kramer RH, Kaufman SJ *et al* (1998) Mutations in the integrin alpha7 gene cause congenital myopathy. *Nat Genet* 19: 94–97

Jeon SA, Kim DW, Cho JY (2019) Neural precursor cell-expressed, developmentally down-regulated 4 (NEDD4) regulates hydrogen

© 2023 The Authors

peroxide-induced cell proliferation and death through inhibition of Hippo signaling. *FASEB J* 33: 14772 – 14783

Judson RN, Tremblay AM, Knopp P, White RB, Urcia R, De Bari C, Zammit PS, Camargo FD, Wackerhage H (2012) The Hippo pathway member Yap plays a key role in influencing fate decisions in muscle satellite cells. *J Cell Sci* 125: 6009 – 6019

Judson RN, Gray SR, Walker C, Carroll AM, Itzstein C, Lionikas A, Zammit PS, De Bari C, Wackerhage H (2013) Constitutive expression of Yes-associated protein (Yap) in adult skeletal muscle fibres induces muscle atrophy and myopathy. *PloS One* 8: e59622

Kamien B, Clayton JS, Lee HS, Abeysuriya D, McNamara E, Martinovic J, Gonzales M, Melki J, Ravenscroft G (2022) Bi-allelic MYH3 loss-of-function variants cause a lethal form of contractures, pterygia, and spondylocarpotarsal fusion syndrome 1B. *Neuromuscul Disord* 32: 445 – 449

Kandasamy S, Adhikary G, Rorke EA, Friedberg JS, Mickle MB, Alexander HR, Eckert RL (2020) The YAP1 signaling inhibitors, verteporfin and CA3, suppress the mesothelioma cancer stem cell phenotype. *Mol Cancer Res* 18: 343 – 351

Kechagia JZ, Ivaska J, Roca-Cusachs P (2019) Integrins as biomechanical sensors of the microenvironment. *Nat Rev Mol Cell Biol* 20: 457 – 473

Keefe AC, Lawson JA, Flygare SD, Fox ZD, Colasanto MP, Mathew SJ, Yandell M, Kardon G (2015) Muscle stem cells contribute to myofibres in sedentary adult mice. *Nat Commun* 6: 7087

Kiema T, Lad Y, Jiang P, Oxley CL, Baldassarre M, Wegener KL, Campbell ID, Ylanne J, Calderwood DA (2006) The molecular basis of filamin binding to integrins and competition with talin. *Mol Cell* 21: 337 – 347

Krakow D, Robertson SP, King LM, Morgan T, Sebald ET, Bertolotto C, Wachsmann-Hogiu S, Acuna D, Shapiro SS, Takafuta T *et al* (2004) Mutations in the gene encoding filamin B disrupt vertebral segmentation, joint formation and skeletogenesis. *Nat Genet* 36: 405 – 410

Lawson CD, Burridge K (2014) The on-off relationship of Rho and Rac during integrin-mediated adhesion and cell migration. *Small GTPases* 5: e27958

Liu H, Dai X, Cao X, Yan H, Ji X, Zhang H, Shen S, Si Y, Zhang H, Chen J *et al* (2018) PRDM4 mediates YAP-induced cell invasion by activating leukocyte-specific integrin beta2 expression. *EMBO Rep* 19: e45180

Llado V, Nakanishi Y, Duran A, Reina-Campos M, Shelton PM, Linares JF, Yajima T, Campos A, Aza-Blanc P, Leitges M *et al* (2015) Repression of intestinal stem cell function and tumorigenesis through direct phosphorylation of beta-catenin and yap by PKCzeta. *Cell Rep* 10: 740 – 754

Lyons GE, Ontell M, Cox R, Sassoon D, Buckingham M (1990) The expression of myosin genes in developing skeletal muscle in the mouse embryo. *J Cell Biol* 111: 1465 – 1476

Mayer U, Saher G, Fassler R, Bornemann A, Echtermeyer F, von der Mark H, Miosge N, Poschl E, von der Mark K (1997) Absence of integrin alpha 7 causes a novel form of muscular dystrophy. *Nat Genet* 17: 318 – 323

Meng Z, Moroishi T, Mottier-Pavie V, Plouffe SW, Hansen CG, Hong AW, Park HW, Mo JS, Lu W, Lu S *et al* (2015) MAP4K family kinases act in parallel to MST1/2 to activate LATS1/2 in the Hippo pathway. *Nat Commun* 6: 8357

Nishio M, Sugimachi K, Goto H, Wang J, Morikawa T, Miyachi Y, Takano Y, Hikasa H, Itoh T, Suzuki SO *et al* (2016) Dysregulated YAP1/TAZ and TGF-beta signaling mediate hepatocarcinogenesis in Mob1a/1b-deficient mice. *Proc Natl Acad Sci USA* 113: E71 – E80

Parsons JT, Horwitz AR, Schwartz MA (2010) Cell adhesion: integrating cytoskeletal dynamics and cellular tension. *Nat Rev Mol Cell Biol* 11: 633 – 643

Racusen RH, Thompson KV (1997) Isolation of myosin and actin from chicken muscle. In *Tested studies for laboratory teaching*, Glase JC (ed), Vol. 18, pp 97 – 111. Proceedings of the 18th Workshop/Conference of the Association for Biology Laboratory Education (ABLE), 322 pages

Rao DS, Kronert WA, Guo Y, Hsu KH, Sarsoza F, Bernstein SI (2019) Reductions in ATPase activity, Actin sliding velocity, and myofibril stability yield muscle dysfunction in Drosophila models of myosin-based Freeman-Sheldon syndrome. *Mol Biol Cell* 30: 30 – 41

Rigueur D, Lyons KM (2014) Whole-mount skeletal staining. *Methods Mol Biol* 1130: 113 – 121

Salah Z, Cohen S, Itzhaki E, Aqeilan RI (2013) NEDD4 E3 ligase inhibits the activity of the Hippo pathway by targeting LATS1 for degradation. *Cell Cycle* 12: 3817 – 3823

Sartorius CA, Lu BD, Acakpo-Satchivi L, Jacobsen RP, Byrnes WC, Leinwand LA (1998) Myosin heavy chains IIa and IId are functionally distinct in the mouse. *J Cell Biol* 141: 943 – 953

Schiaffino S, Rossi AC, Smerdu V, Leinwand LA, Reggiani C (2015) Developmental myosins: expression patterns and functional significance. *Skelet Muscle* 5: 22

Schwander M, Leu M, Stumm M, Dorchies OM, Ruegg UT, Schittny J, Muller U (2003) Beta1 integrins regulate myoblast fusion and sarcomere assembly. *Dev Cell* 4: 673 – 685

Smith LR, Barton ER (2014) SMASH - semi-automatic muscle analysis using segmentation of histology: a MATLAB application. *Skelet Muscle* 4: 21

Song S, Xie M, Scott AW, Jin J, Ma L, Dong X, Skinner HD, Johnson RL, Ding S, Ajani JA (2018) A novel YAP1 inhibitor targets CSC-enriched radiation-resistant cells and exerts strong antitumor activity in esophageal adenocarcinoma. *Mol Cancer Ther* 17: 443 – 454

Sun Z, Guo SS, Fassler R (2016) Integrin-mediated mechanotransduction. *J Cell Biol* 215: 445 – 456

Tajsharghi H, Kimber E, Kroksmark AK, Jerre R, Tulinius M, Oldfors A (2008) Embryonic myosin heavy-chain mutations cause distal arthrogryposis and developmental myosin myopathy that persists postnatally. *Arch Neurol* 65: 1083 – 1090

Tang Y, Rowe RG, Botvinick EL, Kurup A, Putnam AJ, Seiki M, Weaver VM, Keller ET, Goldstein S, Dai J *et al* (2013) MT1-MMP-dependent control of skeletal stem cell commitment via a beta1-integrin/YAP/TAZ signaling axis. *Dev Cell* 25: 402 – 416

Taverna D, Disatnik MH, Rayburn H, Bronson RT, Yang J, Rando TA, Hynes RO (1998) Dystrophic muscle in mice chimeric for expression of alpha5 integrin. *J Cell Biol* 143: 849 – 859

Toydemir RM, Rutherford A, Whitby FG, Jorde LB, Carey JC, Bamshad MJ (2006) Mutations in embryonic myosin heavy chain (MYH3) cause Freeman–Sheldon syndrome and Sheldon–Hall syndrome. *Nat Genet* 38: 561 – 565

Varzavand A, Hacker W, Ma D, Gibson-Corley K, Hawayek M, Tayh OJ, Brown JA, Henry MD, Stipp CS (2016) $\alpha3\beta1$ integrin suppresses prostate cancer metastasis via regulation of the hippo pathway. *Cancer Res* 76: 6577 – 6587

Wang L, Luo JY, Li B, Tian XY, Chen LJ, Huang Y, Liu J, Deng D, Lau CW, Wan S *et al* (2016) Integrin-YAP/TAZ-JNK cascade mediates atheroprotective effect of unidirectional shear flow. *Nature* 540: 579 – 582

Wang J, Zhang J, Xu R, Chen TG, Zhou KS, Zhang HH (2018) Measurement of scoliosis Cobb angle by end vertebra tilt angle method. *J Orthop Surg Res* 13: 223

Watt KI, Turner BJ, Hagg A, Zhang X, Davey JR, Qian H, Beyer C, Winbanks CE, Harvey KF, Gregorevic P (2015) The Hippo pathway effector YAP is a critical regulator of skeletal muscle fibre size. *Nat Commun* 6: 6048

© 2023 The Authors

White J, Barro MV, Makarenkova HP, Sanger JW, Sanger JM (2014) Localization of sarcomeric proteins during myofibril assembly in cultured mouse primary skeletal myotubes. *Anat Rec* 297: 1571 – 1584

Whittle J, Antunes L, Harris M, Upshaw Z, Sepich DS, Johnson AN, Mokalled M, Solnica-Krezel L, Dobbs MB, Gurnett CA (2020) MYH3-associated distal arthrogryposis zebrafish model is normalized with para-aminoblebbistatin. *EMBO Mol Med* 12: e12356

Whittle J, Johnson A, Dobbs MB, Gurnett CA (2021) Models of distal arthrogryposis and lethal congenital contracture syndrome. *Genes* 12: 943

Yamashiro Y, Thang BQ, Ramirez K, Shin SJ, Kohata T, Ohata S, Nguyen TAV, Ohtsuki S, Nagayama K, Yanagisawa H (2020) Matrix mechanotransduction mediated by thrombospondin-1/integrin/YAP in the vascular remodeling. *Proc Natl Acad Sci USA* 117: 9896 – 9905

Zhang L, Tang F, Terracciano L, Hynx D, Kohler R, Bichet S, Hess D, Cron P, Hemmings BA, Hergovich A *et al* (2015) NDR functions as a physiological YAP1 kinase in the intestinal epithelium. *Curr Biol* 25: 296 – 305

Zhang L, Noguchi YT, Nakayama H, Kaji T, Tsujikawa K, Ikemoto-Uezumi M, Uezumi A, Okada Y, Doi T, Watanabe S *et al* (2019) The CalcR-PKA-Yap1 axis is critical for maintaining quiescence in muscle stem cells. *Cell Rep* 29: e2155

Zhao B, Wei X, Li W, Udan RS, Yang Q, Kim J, Xie J, Ikenoue T, Yu J, Li L *et al* (2007) Inactivation of YAP oncoprotein by the Hippo pathway is involved in cell contact inhibition and tissue growth control. *Genes Dev* 21: 2747 – 2761

Zhao B, Li L, Tumaneng K, Wang CY, Guan KL (2010) A coordinated phosphorylation by Lats and CK1 regulates YAP stability through SCF (beta-TRCP). *Genes Dev* 24: 72 – 85

Zieba J, Forlenza KN, Khatra JS, Sarukhanov A, Duran I, Rigueur D, Lyons KM, Cohn DH, Merrill AE, Krakow D (2016) TGFβ and BMP dependent cell fate changes due to loss of Filamin B produces disc degeneration and progressive vertebral fusions. *PLoS Genet* 12: e1005936

Zieba J, Zhang W, Chong JX, Forlenza KN, Martin JH, Heard K, Grange DK, Butler MG, Kleefstra T, Lachman RS *et al* (2017) A postnatal role for embryonic myosin revealed by MYH3 mutations that alter TGFβ signaling and cause autosomal dominant spondylocarpotarsal synostosis. *Sci Rep* 7: 41803

License: This is an open access article under the terms of the Creative Commons Attribution License, which permits use, distribution and reproduction in any medium, provided the original work is properly cited.

Expanded View Figures

Figure EV1. Loss of MyHC-embryonic leads to alterations in myofiber number, size and function.

A, B Quantification of the number of myofibers normalized to cross-sectional area (CSA) (mm^2) (n = 4 mice per genotype) through the gastrocnemius muscle of *Myh3*$^{+/+}$ and *Myh3*$^{\Delta/\Delta}$ mice at 8–10 weeks (A) and 6 months (B) of age.

C, D Quantification of the number of myofibers normalized to cross-sectional area (CSA) (mm^2) (n = 4 mice per genotype) through the soleus muscle of *Myh3*$^{+/+}$ and *Myh3*$^{\Delta/\Delta}$ mice at 8–10 weeks (C) and 6 months (D) of age.

E–I Quantification of the number of myofibers grouped according to area through the TA muscle (n = 4 mice per genotype) at 8–10 weeks of age (E), through the gastrocnemius muscle (n = 3 mice per genotype) at 8–10 weeks (F) and 6 months (G) of age, and through the soleus muscle (n = 3 mice per genotype) at 8–10 weeks (H) and 6 months (I) of age of *Myh3*$^{+/+}$ and *Myh3*$^{\Delta/\Delta}$ mice.

J–K Bright-field images of skeletal preparations of representative *Myh3*$^{+/+}$ and *Myh3*$^{\Delta/\Delta}$ 4-week-old mice showing the cervical region, stained with Alcian blue and Alizarin red.

L, M Quantification of the percentage of cervical (L) and lumbar (M) vertebral fusion in *Myh3*$^{+/+}$ and *Myh3*$^{\Delta/\Delta}$ mice.

N Graph depicting results from treadmill exhaustion test measured as distance traveled (meters) by 4-month-old *Myh3*$^{+/+}$ and *Myh3*$^{\Delta/\Delta}$ mice.

O Plots for myosin ATPase activity from hind limb muscle of embryonic day (E) 16.5 *Myh3*$^{+/+}$ and *Myh3*$^{\Delta/\Delta}$ embryos (n = 3 mice per genotype).

Data information: Data are presented as mean ± SEM. Student's t-test was performed, with $P \leq 0.05$ considered significant.

© 2023 The Authors

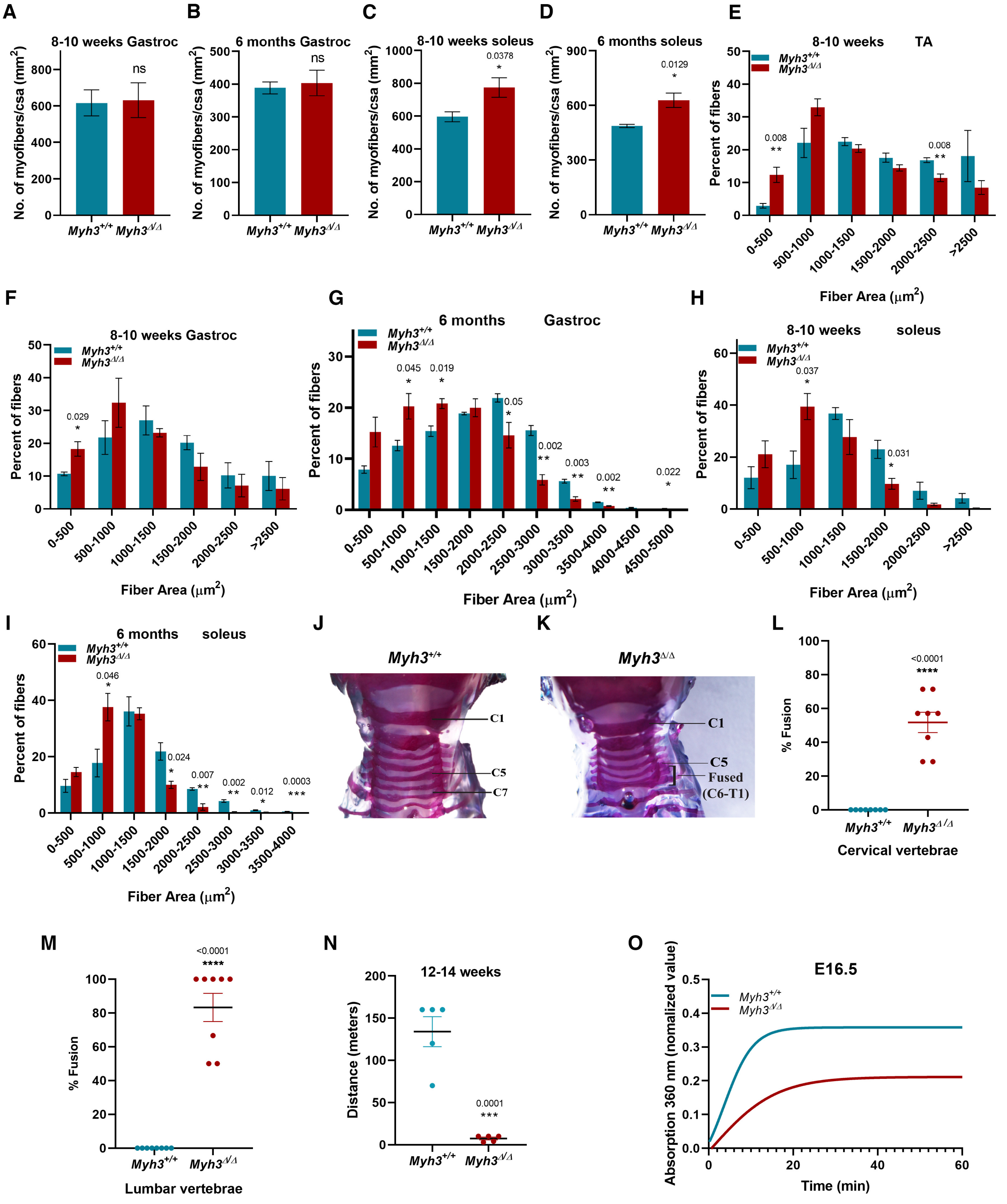
A
8-10 weeks Gastroc
No. of myofibers/csa (mm²)
ns
Myh3+/+ Myh3Δ/Δ
B
6 months Gastroc
ns
C
8-10 weeks soleus
0.0378
D
6 months soleus
0.0129
E
8-10 weeks TA
Percent of fibers
0.008
0.008
Fiber Area (μm²)
F
8-10 weeks Gastroc
0.029
G
6 months Gastroc
0.045
0.019
0.05
0.002
0.003
0.002
0.022
H
8-10 weeks soleus
0.037
0.031
I
6 months soleus
0.046
0.024
0.007
0.002
0.012
0.0003
J
Myh3+/+
C1
C5
C7
K
MyH3Δ/Δ
C1
C5
Fused
(C6-T1)
L
% Fusion
<0.0001
Cervical vertebrae
M
<0.0001
Lumbar vertebrae
N
12-14 weeks
Distance (meters)
0.0001
O
E16.5
Absorption 360 nm (nomalized value)
Time (min)

Figure EV1.

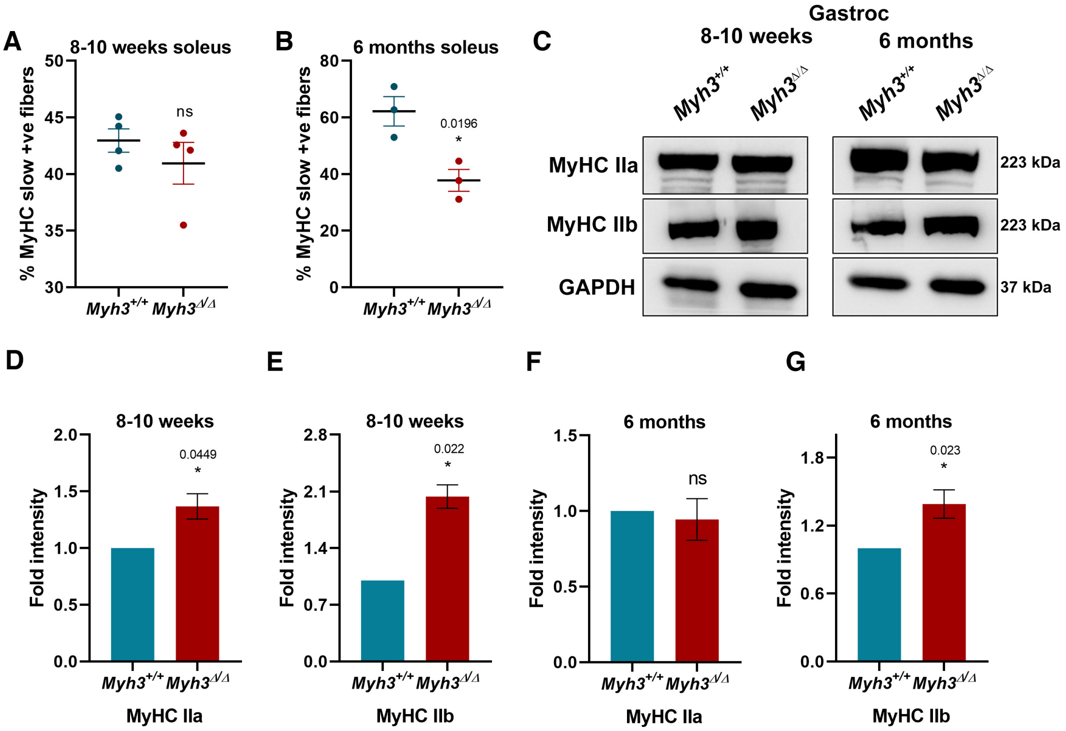

Figure EV2. Loss of MyHC-embryonic function leads to muscle fiber type changes.

A, B Quantification of the percentage of MyHC-slow-positive fibers normalized to total number of fibers through the soleus muscle of *Myh3*$^{+/+}$ and *Myh3*$^{\Delta/\Delta}$ mice at 8–10 weeks and 6 months of age, respectively.

C–G Representative western blots for MyHC-IIa, -IIb, and GAPDH using protein lysates from the gastrocnemius muscle of *Myh3*$^{+/+}$ and *Myh3*$^{\Delta/\Delta}$ mice at 8–10 weeks and 6 months of age (C) and their densitometric quantification (D–G) (n = 4 mice per genotype).

Data information: Data are presented as mean ± SEM. Student's t-test was performed, with $P \leq 0.05$ considered significant.

 © 2023 The Authors

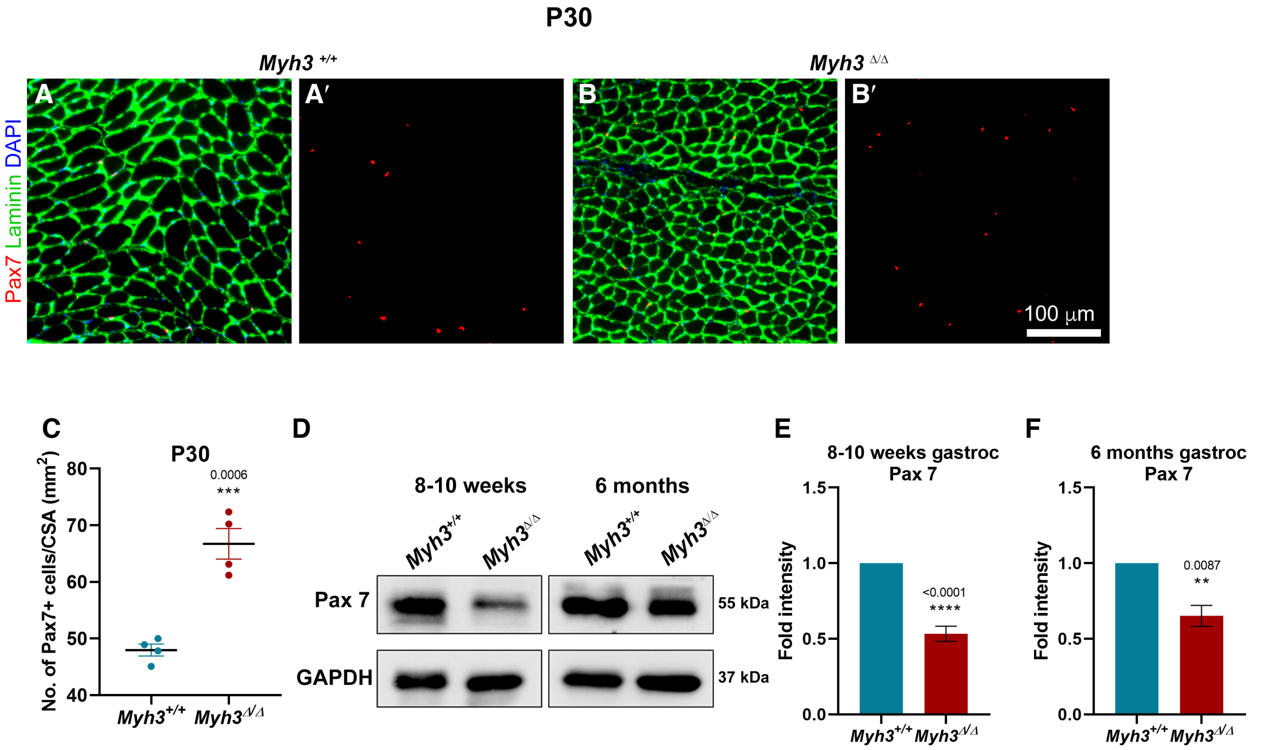

Figure EV3. Loss of MyHC-embryonic function leads to alterations in satellite cell numbers.

A–C Representative fluorescent micrographs of transverse sections through the TA muscle of *Myh3*$^{+/+}$ and *Myh3*$^{\Delta/\Delta}$ mice at postnatal Day 30 stained with Pax7 (red), Laminin (green) and DAPI (blue) (A–B′), and quantification of the number of Pax7-positive cells per unit area (mm^2) (C).

D–F Representative western blots for Pax7 and GAPDH using protein lysates from the gastrocnemius muscle of *Myh3*$^{+/+}$ and *Myh3*$^{\Delta/\Delta}$ mice at 8–10 weeks and 6 months of age (D) and their densitometric quantification (E, F) (n = 4 mice per genotype in E and n = 5 mice per genotype in F).

Data information: Data are presented as mean ± SEM. Student's *t*-test was performed, with $P \leq 0.05$ considered significant. Scale bar: 100 μm (B′).

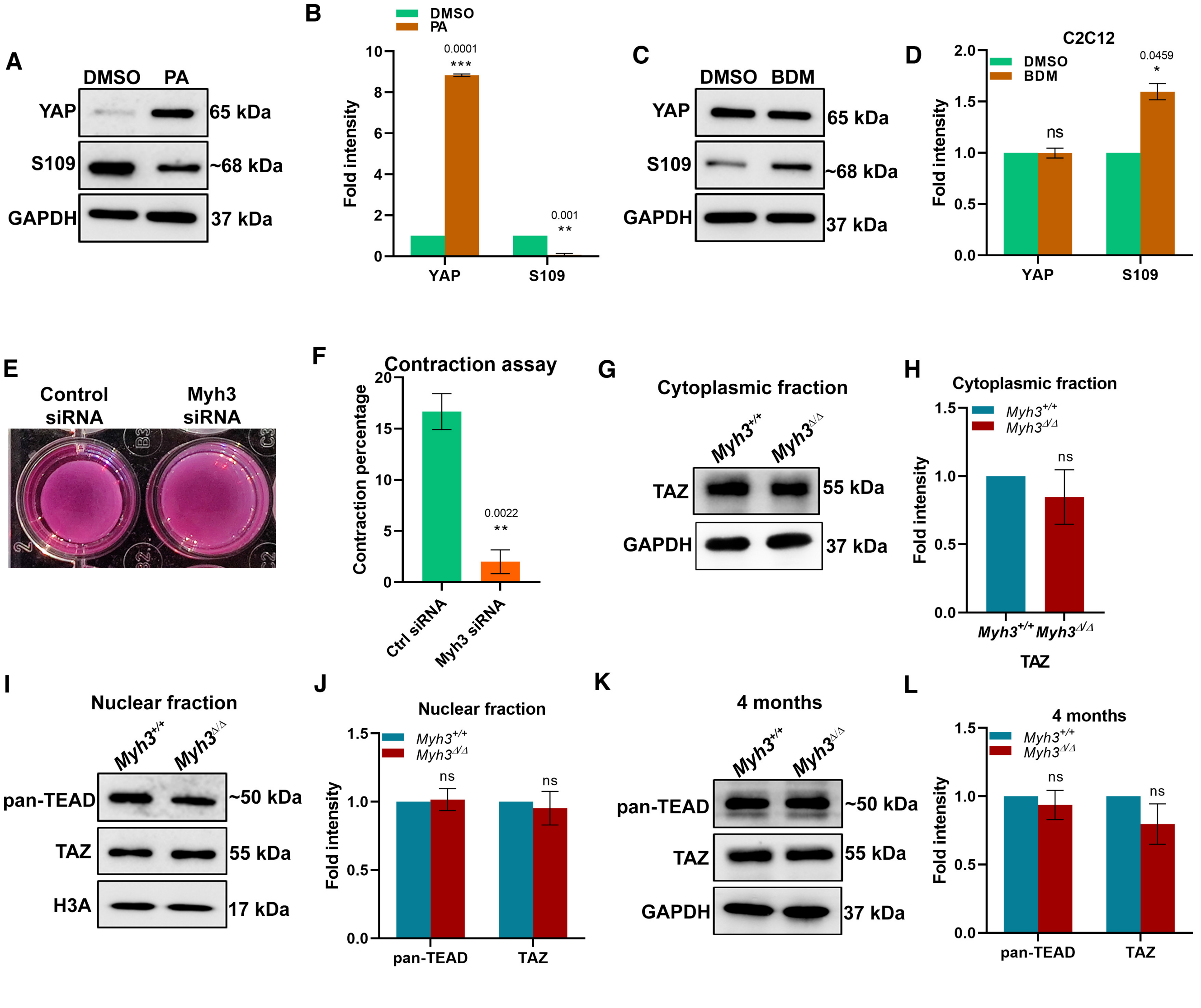

Figure EV4. Myosin inhibitors modulate YAP expression and activation.

A, B Representative western blots for total YAP, phospho-YAP (S109), and GAPDH using protein lysates from C2C12 cells treated with DMSO or para-aminoblebbistatin (A) and their densitometric quantification (B) (n = 3 biological replicates).

C, D Representative western blots for total YAP, phospho-YAP (S109), and GAPDH using protein lysates from C2C12 cells treated with DMSO or 2,3-Butanedione monoxime (BDM) (C) and their densitometric quantification (D) (n = 4 biological replicates).

E, F Representative micrograph showing collagen matrix following culture of control or *Myh3* siRNA-treated C2C12 cells (E) and quantification of the percentage of matrix contraction (F). (n = 3 biological replicates).

G, H Representative western blots for TAZ and GAPDH in the cytoplasmic fraction (G), isolated from the TA muscle of 4-month-old $Myh3^{+/+}$ and $Myh3^{\Delta/\Delta}$ mice (E) and densitometric quantification (H) (n = 5 mice per genotype).

I–L Representative western blots for pan-TEAD, TAZ, and histone H3A in the nuclear fraction (I) and pan-TEAD, TAZ, and GAPDH in the total lysate (K) isolated from the TA muscle of 4-month-old $Myh3^{+/+}$ and $Myh3^{\Delta/\Delta}$ mice and their respective densitometric quantification (J, L) (n = 4 mice per genotype in J and n = 5 mice per genotype in L).

 © 2023 The Authors

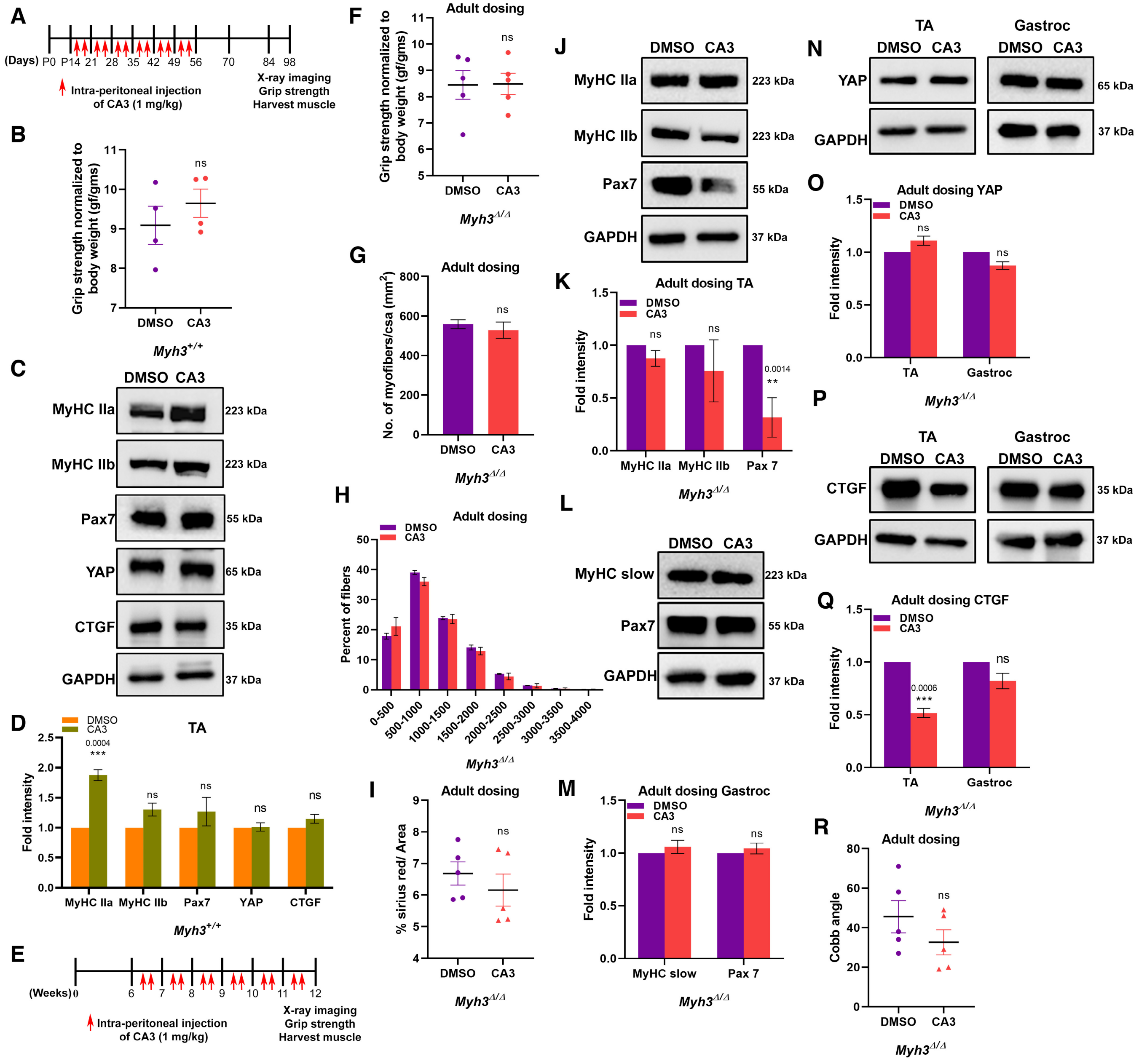
A
(Days) P0 P14 21 28 35 42 49 56 70 84 98
Intra-peritoneal injection of CA3 (1 mg/kg)
X-ray imaging
Grip strength
Harvest muscle
B
Grip strength normalized to body weight (gf/gms)
ns
DMSO CA3
Myh3+/+
C
DMSO CA3
MyHC IIa 223 kDa
MyHC IIb 223 kDa
Pax7 55 kDa
YAP 65 kDa
CTGF 35 kDa
GAPDH 37 kDa
D
DMSO
CA3
TA
Fold intensity
0.0004
***
ns
MyHC IIa MyHC IIb Pax7 YAP CTGF
Myh3+/+
E
(Weeks) 0 6 7 8 9 10 11 12
Intra-peritoneal injection of CA3 (1 mg/kg)
X-ray imaging
Grip strength
Harvest muscle
F
Adult dosing
Grip strength normalized to body weight (gf/gms)
ns
DMSO CA3
Myh3Δ/Δ
G
Adult dosing
No. of myofibers/csa (mm2)
ns
DMSO CA3
Myh3Δ/Δ
H
Adult dosing
DMSO
CA3
Percent of fibers
0-500 500-1000 1000-1500 1500-2000 2000-2500 2500-3000 3000-3500 3500-4000
Myh3Δ/Δ
I
Adult dosing
% sirius red/ Area
ns
DMSO CA3
Myh3Δ/Δ
J
DMSO CA3
MyHC IIa 223 kDa
MyHC IIb 223 kDa
Pax7 55 kDa
GAPDH 37 kDa
K
Adult dosing TA
DMSO
CA3
Fold intensity
ns
0.0014
**
MyHC IIa MyHC IIb Pax 7
Myh3Δ/Δ
L
DMSO CA3
MyHC slow 223 kDa
Pax7 55 kDa
GAPDH 37 kDa
M
Adult dosing Gastroc
DMSO
CA3
Fold intensity
ns
MyHC slow Pax 7
Myh3Δ/Δ
N
TA Gastroc
DMSO CA3 DMSO CA3
YAP 65 kDa
GAPDH 37 kDa
O
Adult dosing YAP
DMSO
CA3
Fold intensity
ns
TA Gastroc
Myh3Δ/Δ
P
TA Gastroc
DMSO CA3 DMSO CA3
CTGF 35 kDa
GAPDH 37 kDa
Q
Adult dosing CTGF
DMSO
CA3
Fold intensity
0.0006
***
ns
TA Gastroc
Myh3Δ/Δ
R
Adult dosing
Cobb angle
ns
DMSO CA3
Myh3Δ/Δ

Figure EV5.

◀ **Figure EV5. Administration of CA3 in adults fails to rescue most abnormalities seen in *Myh3*$^{\Delta/\Delta}$ mice.**

A Schematic depicting CA3 dosing strategy starting from postnatal day 15.

B Quantification of grip strength normalized to body weight of *Myh3*$^{+/+}$ mice at 12–14 weeks of age, treated with DMSO (vehicle) or CA3.

C, D Representative western blots for MyHC-IIa, -IIb, Pax7, YAP, CTGF, and GAPDH using protein lysates from the TA muscle of *Myh3*$^{+/+}$ mice at 12–14 weeks of age, treated with DMSO or CA3 (C) and their densitometric quantification (D) (n = 4 mice per genotype).

E Schematic depicting CA3 dosing strategy starting from 6 weeks after birth (adult dosing).

F Quantification of grip strength normalized to body weight of *Myh3*$^{\Delta/\Delta}$ mice at 12–14 weeks of age, treated with DMSO (vehicle) or CA3 starting from 6 weeks after birth.

G Quantification of the number of myofibers normalized to cross-sectional area (CSA) (mm^2) through the TA muscle of *Myh3*$^{\Delta/\Delta}$ mice at 12–14 weeks of age, treated with DMSO or CA3 starting from 6 weeks after birth (n = 5 mice per genotype).

H Quantification of the number of myofibers grouped according to myofiber area through the TA muscle of *Myh3*$^{\Delta/\Delta}$ mice at 12–14 weeks of age, treated with DMSO or CA3 starting from 6 weeks after birth (n = 3 mice per genotype).

I Quantification of the percentage of Sirius red-positive area as a fraction of total area in transverse sections through the TA muscle of *Myh3*$^{\Delta/\Delta}$ mice at 12–14 weeks of age, treated with DMSO or CA3 starting from 6 weeks after birth.

J, K Representative western blots for MyHC-IIa, -IIb, Pax7, and GAPDH using protein lysates from the TA muscle of *Myh3*$^{\Delta/\Delta}$ mice at 12–14 weeks of age, treated with DMSO or CA3 starting from 6 weeks after birth (J) and their densitometric quantification (K) (n = 5 mice per genotype).

L, M Representative western blots for MyHC-slow, Pax7, and GAPDH using protein lysates from the gastrocnemius muscle of *Myh3*$^{\Delta/\Delta}$ mice at 12–14 weeks of age, treated with DMSO or CA3 starting from 6 weeks after birth (L) and their densitometric quantification (M) (n = 5 mice per genotype).

N, O Representative western blots for total YAP and GAPDH in the TA and the gastrocnemius (N) protein lysates of *Myh3*$^{\Delta/\Delta}$ mice at 12–14 weeks of age, treated with DMSO or CA3 starting from 6 weeks after birth and their densitometric quantification (O) (n = 6 mice per genotype).

P, Q Representative western blots for CTGF and GAPDH in the TA and the gastrocnemius (P) protein lysates of *Myh3*$^{\Delta/\Delta}$ mice at 12–14 weeks of age, treated with DMSO or CA3 starting from 6 weeks after birth and their densitometric quantification (Q) (n = 4 mice per genotype).

R Quantification of the Cobb angle of *Myh3*$^{\Delta/\Delta}$ mice at 12–14 weeks of age, treated with DMSO or CA3 starting from 6 weeks after birth.

Data information: Data are presented as mean ± SEM. Student's t-test was performed, with $P \leq 0.05$ considered significant.

© 2023 The Authors

Musculoskeletal defects associated with Myosin Heavy Chain-embryonic loss of function are mediated by the YAP signaling pathway.

Anushree Bharadwaj[1,*], Jaydeep Sharma[1,*], Jagriti Singh[1], Mahima Kumari[1], Tanushri Dargar[1,2], Bhargab Kalita[1,3] and Sam J. Mathew[1,#]

Appendix

Appendix Table S1: Antibodies used for immunofluorescence and western blots

Appendix Table S2: List of primers

Appendix Table S1: Antibodies used for immunofluorescence and western blots

Antibody	Type	Source	Product No.	Working Dilution
**Primary antibodies**				
Pax7	Mouse IgG1	Developmental Studies Hybridoma Bank	PAX7	1:10 (IF, WB)
Laminin	Rabbit polyclonal	Sigma	L9393	1:400 (IF)
MyHC slow	Mouse IgG1	Sigma	M8421 (NOQ7.5.4D)	1:1000 (IF, WB)
MyHC-IIa	Mouse IgG1	Developmental Studies Hybridoma Bank	SC-71	1:10 (IF, WB)
MyHC-IIb	Mouse IgM	Developmental Studies Hybridoma Bank	BFF3	1:10 (IF, WB)
MyHC-IIx	Mouse IgM	Developmental Studies Hybridoma Bank	6H1	1:50 (IF, WB)
Talin 1	Rabbit IgG1	Cloud-Clone Corp	RPA278Mu01	1:1000 (WB)
YAP (D8H1X)	Rabbit IgG	Cell Signaling Technology	14074	1:1000 (WB)
P-YAP (S109)	Rabbit IgG	Cell Signaling Technology	46931	1:1000 (WB)
P-YAP (S127)	Rabbit IgG	Cell Signaling Technology	13008	1:1000 (WB)
CTGF (D8Z8U)	Rabbit IgG	Cell Signaling Technology	86641	1:1000 (WB)
CYR61 (D4H5D)	Rabbit IgG	Cell Signaling Technology	14479	1:1000 (WB)
NEDD4	Rabbit IgG	Cell Signaling Technology	2740	1:1000 (WB)
Anti-Histone H3	Rabbit IgG	Abcam	Ab1791	1:2000 (WB)
GAPDH	Rabbit IgG	Cloud-Clone Corp.	PAB932Mu01	1:10,000 (WB)
Filamin B	Rabbit IgG	Cell Signaling Technology	12979S	1:1000 (WB)
Pan-TEAD (D3F7L)	Rabbit IgG	Cell Signaling Technology	13295	1:1000 (WB)
TAZ (D316D)	Rabbit IgG	Cell Signaling Technology	70148	1:1000 (WB)
Caspase3	Rabbit IgG	Cloud-Clone Corp.	PAA626Mu01	1:1000 (WB)
MyoD	Rabbit IgG	Santa Cruz	Sc-377460	1:400 (WB)

Secondary antibodies				
Cy2/Cy3 conjugated Goat anti-rabbit	Goat	Jackson ImmunoResearch Laboratories	111-225-144/ 111-165-144	1:500 (IF)
Biotin conjugated Goat anti-mouse	Goat	Jackson ImmunoResearch Laboratories	115-065-020	1:500 (IF)
Biotin conjugated Goat anti-rabbit	Goat	Jackson ImmunoResearch Laboratories	111-065-144	1:500 (IF)
Cy2 conjugated streptavidin	-	Jackson ImmunoResearch Laboratories	016-220-084	1:500 (IF)
Cy3 conjugated streptavidin	-	Jackson ImmunoResearch Laboratories	016-160-084	1:500 (IF)
Peroxidase-AffiniPure Goat anti-rabbit	Goat	Jackson ImmunoResearch Laboratories	111-035-144	1:20,000 (WB)
Peroxidase-AffiniPure Goat anti-mouse	Goat	Jackson ImmunoResearch Laboratories	111-035-003	1:20,000 (WB)

Appendix Table S2: List of primers

Gene	Direction	Primer sequence 5' to 3'	Product size (bp) using cDNA template
CTGF	Forward<br>Reverse	TGGAAGACACATTTGGC<br>GTCATTGGTAACTCGGGT	122
CYR61	Forward<br>Reverse	GAGGCTTCCTGTCTTTGGCAC<br>ACTCTGGGTTGTCATTGGTAA	155
AMOT	Forward<br>Reverse	GATGTGCAACCCAGATAAGCC<br>TCTCTGCATCAGGCTCTTGC	101
MOB1A	Forward<br>Reverse	TCGGAAGTGGCAATCTGAGG<br>CAGCTTGCCTCAGTGCAGAA	145
MOB1B	Forward<br>Reverse	CTACGGATGGCTGTCATGCT<br>GACATCACCGGACAGCTCTC	143
