# Supplementary figures and images for: Musculoskeletal defects associated with myosin heavy chain‐embryonic loss of function are mediated by the YAP signaling pathway
Source: EMBO Mol Med. 2023 Jul 26;15(9):e17187. doi: 10.15252/emmm.202217187 (PMC10493586; doi:10.15252/emmm.202217187)

Figure 1: I-J

6 months TA

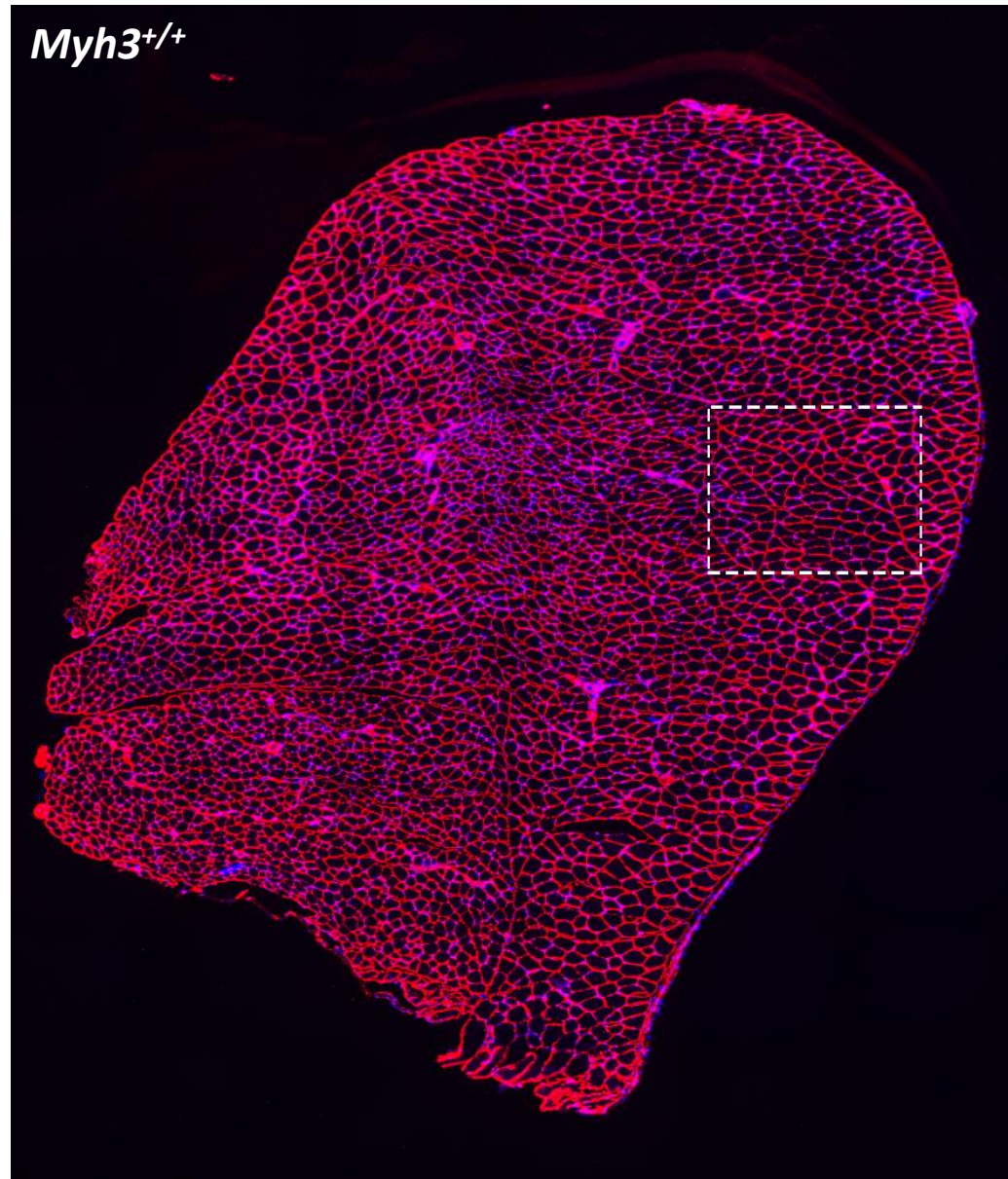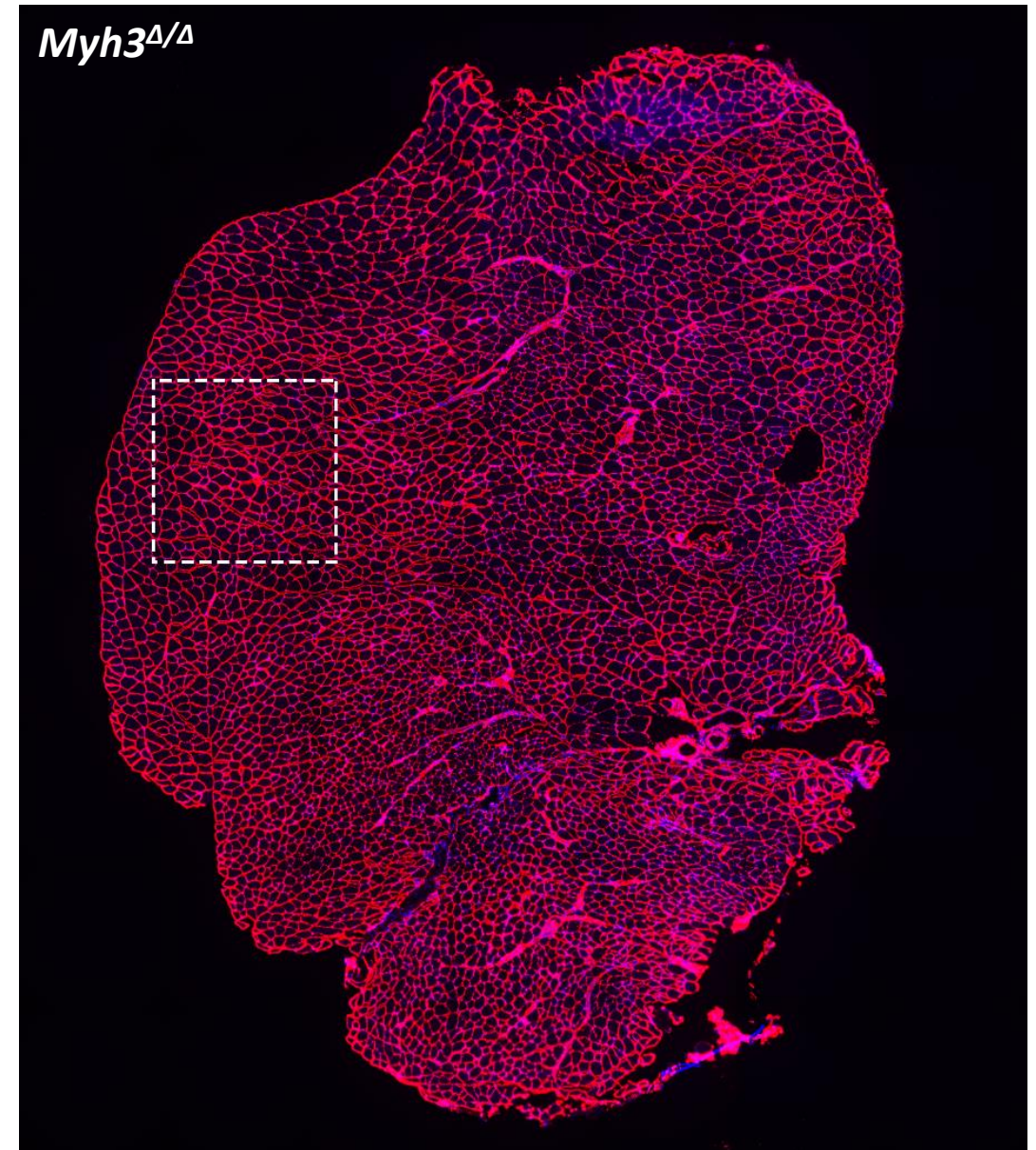

Supplement: Supplementary file 4 — Source Data for Figure 1 [file EMMM-15-e17187-s010.zip › Figure1_Source_Data/Figure1_IF.pdf]

Figure 4E

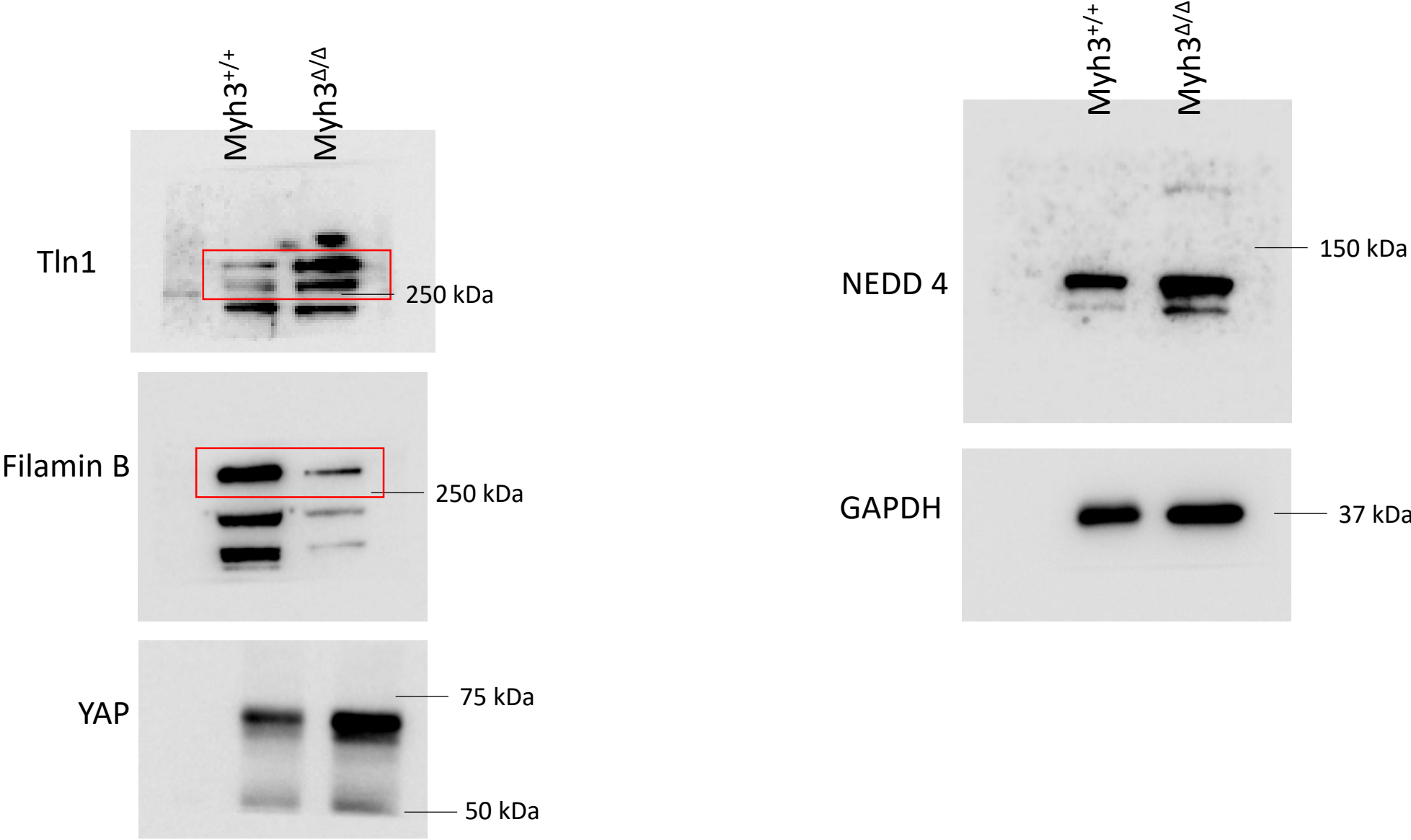

Figure 4H

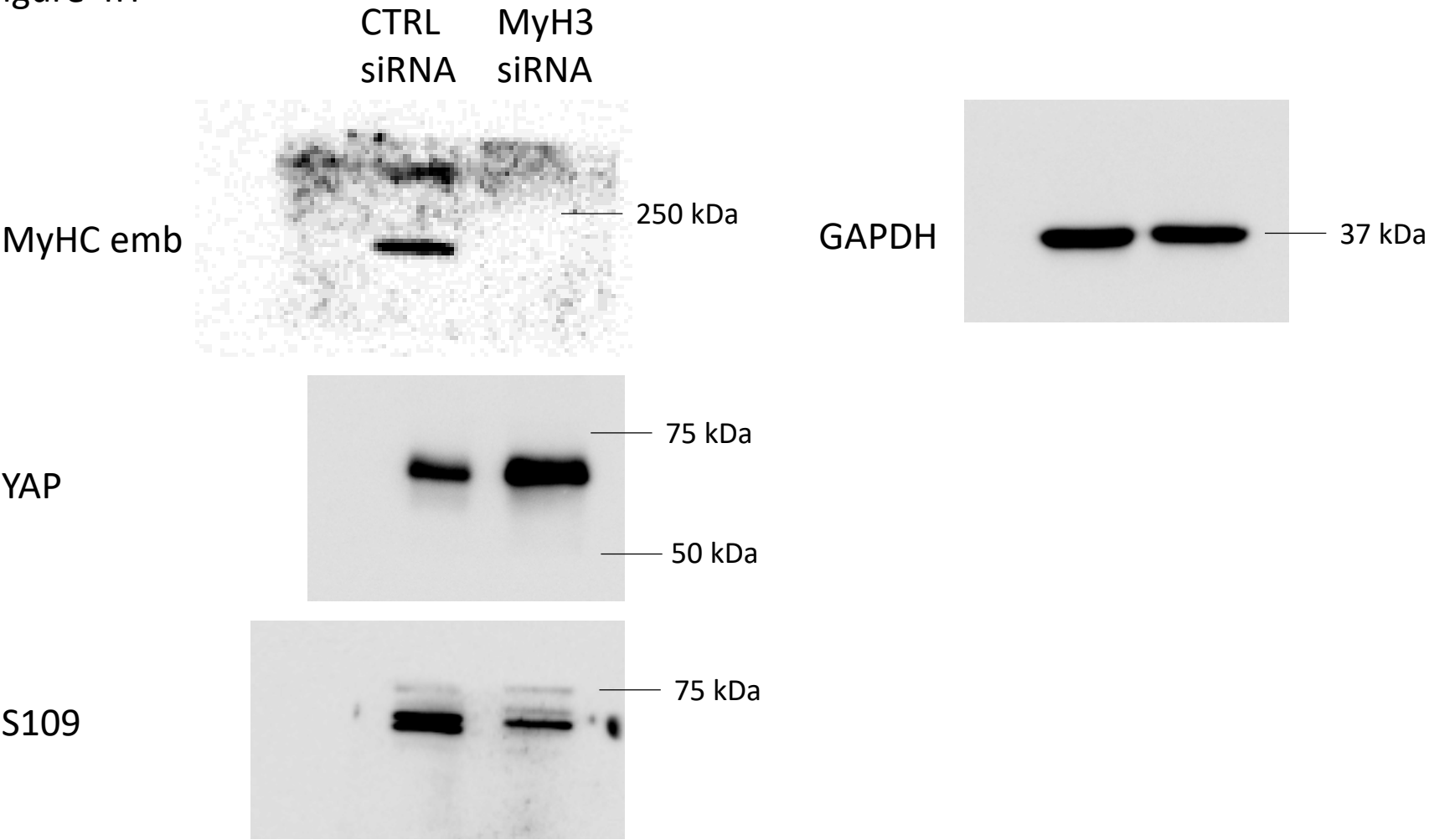

Figure 4J

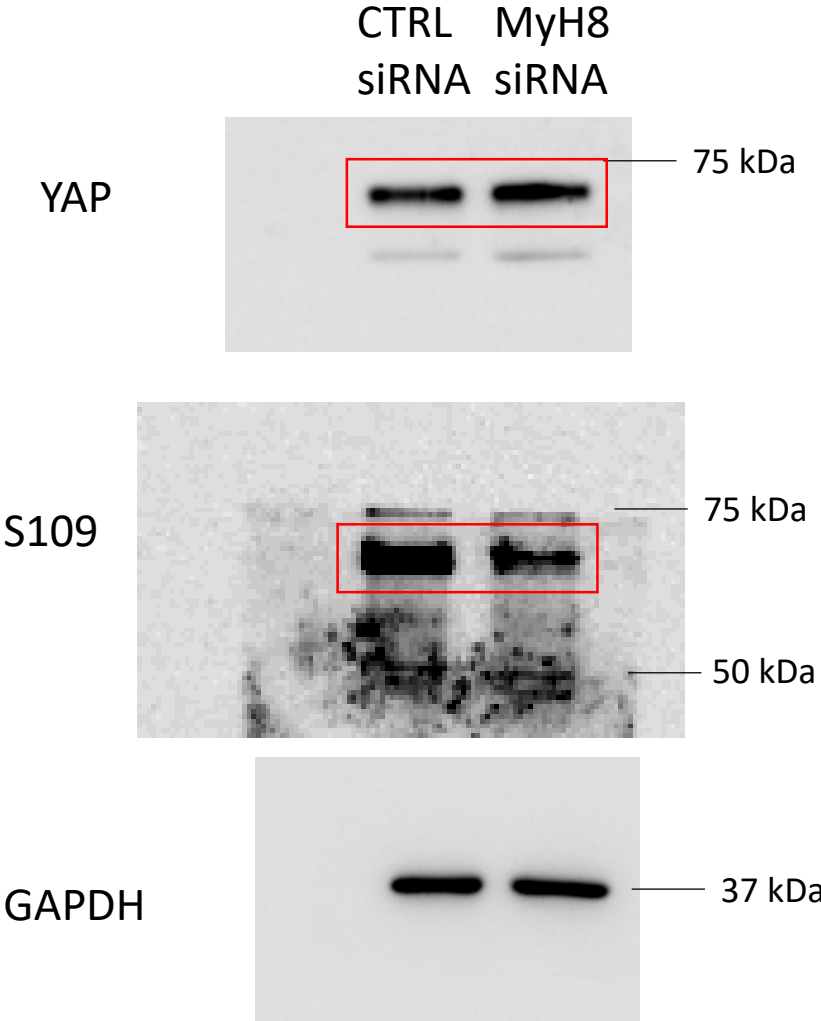

Supplement: Supplementary file 7 — Source Data for Figure 4 [file EMMM-15-e17187-s005.zip › Figure4_Source_Data/Figure4_Western blots.pdf]

Figure 5A

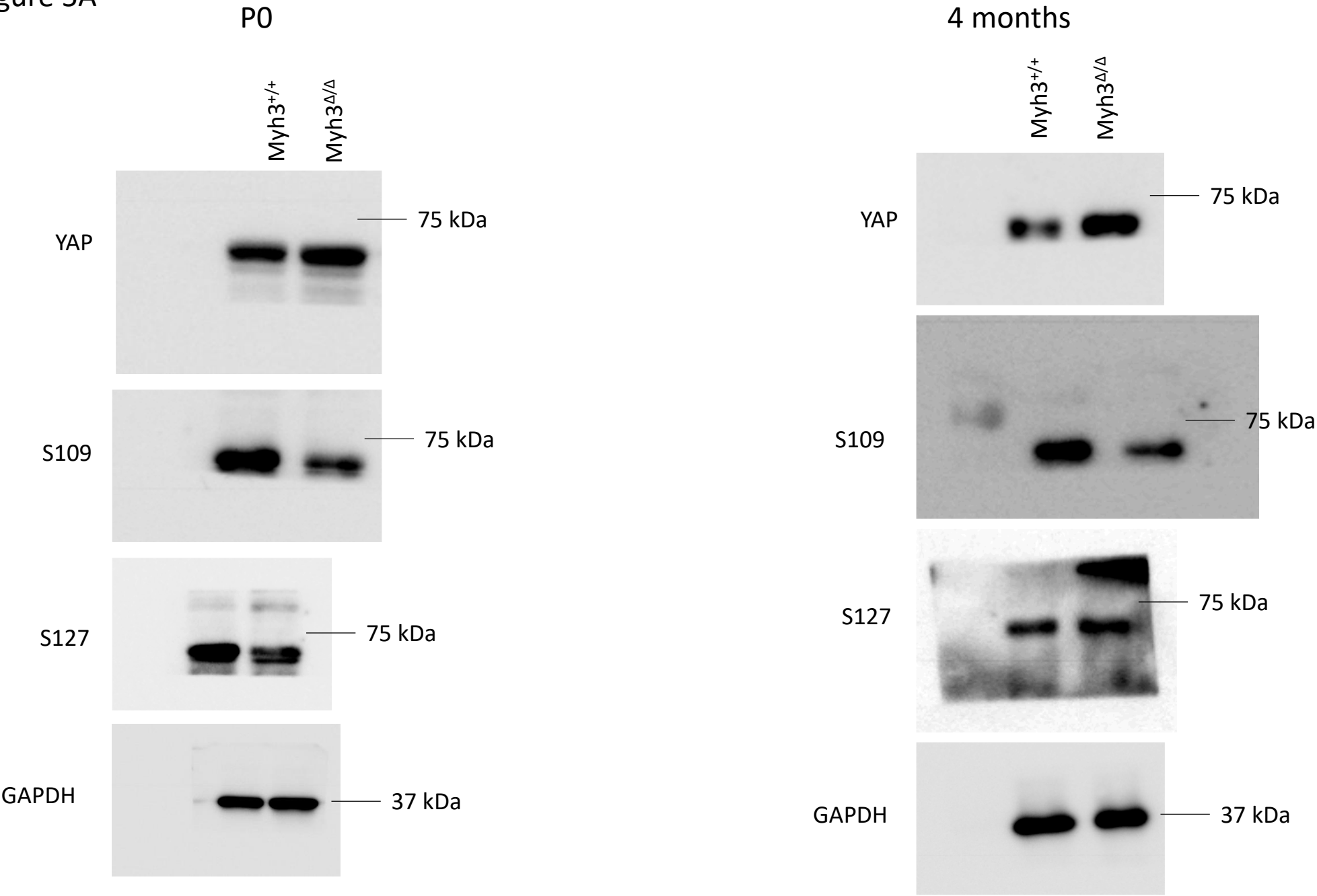

Figure 5E-F

Nuclear fraction

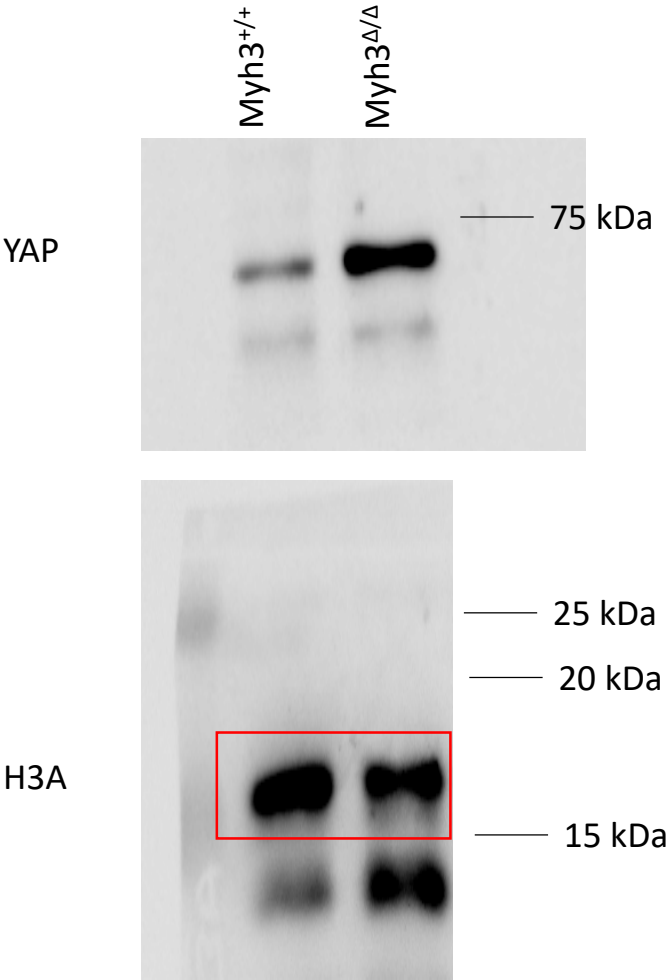

Cytoplasmic fraction

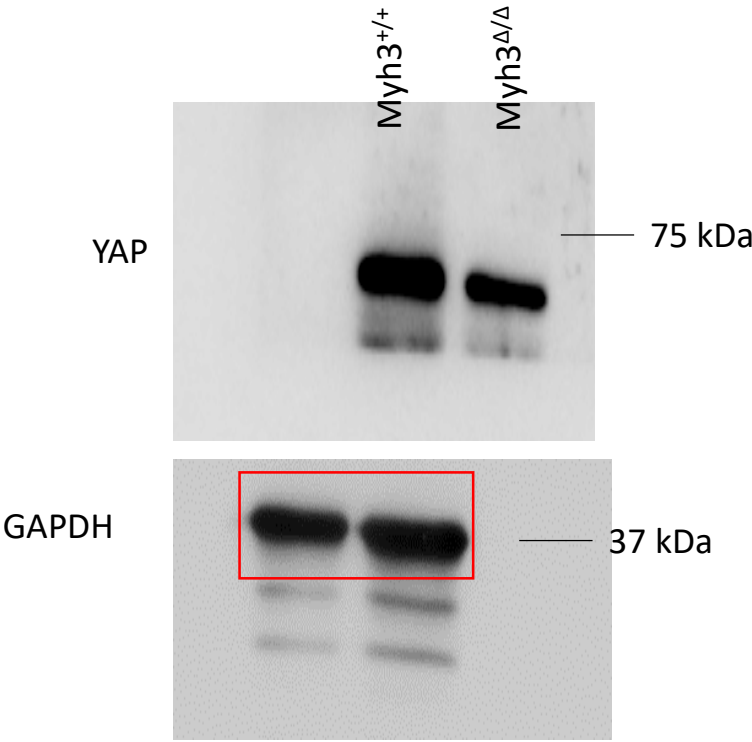

Figure 5J, K

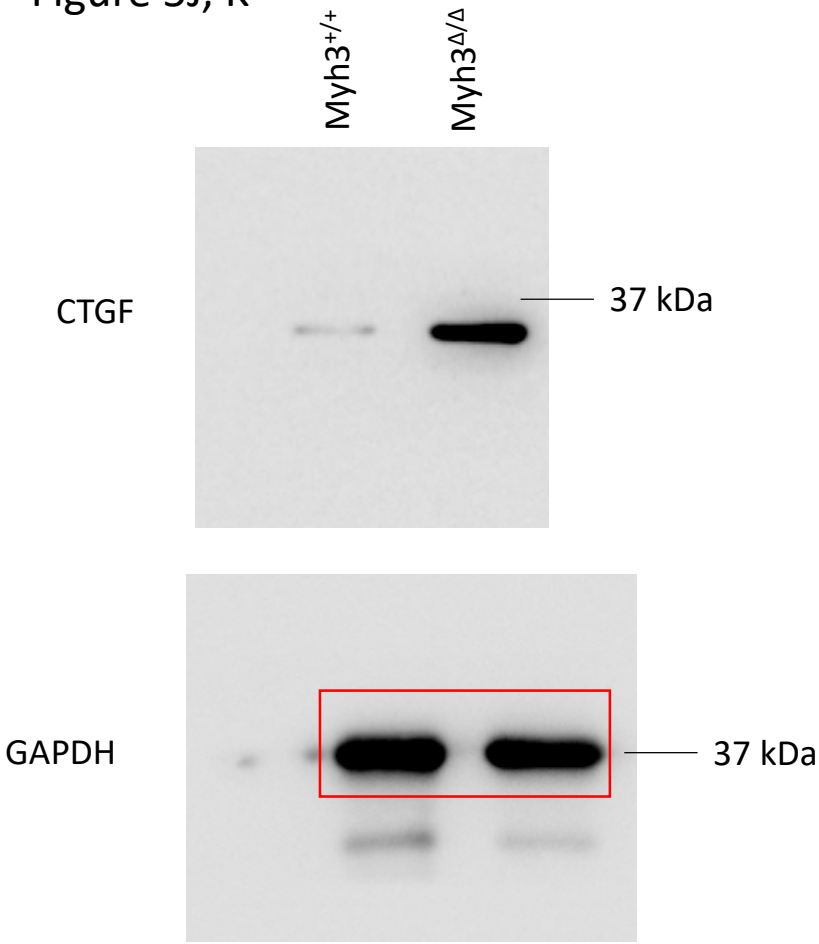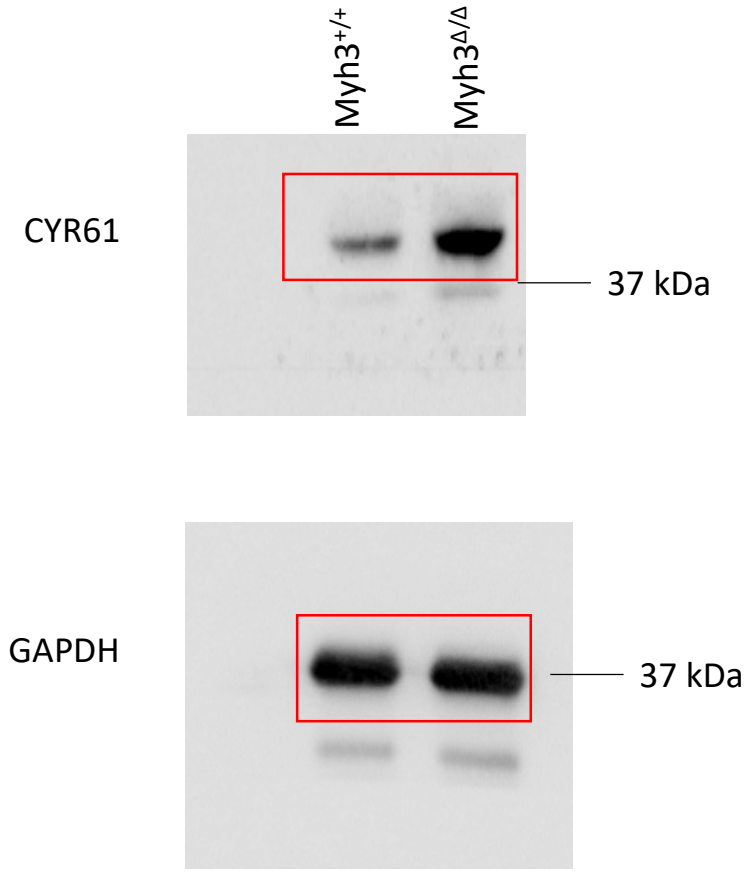

Supplement: Supplementary file 8 — Source Data for Figure 5 [file EMMM-15-e17187-s002.zip › Figure5_Source_Data/Figure5_Western blots.pdf]

Figure 6 D-E

DMSO

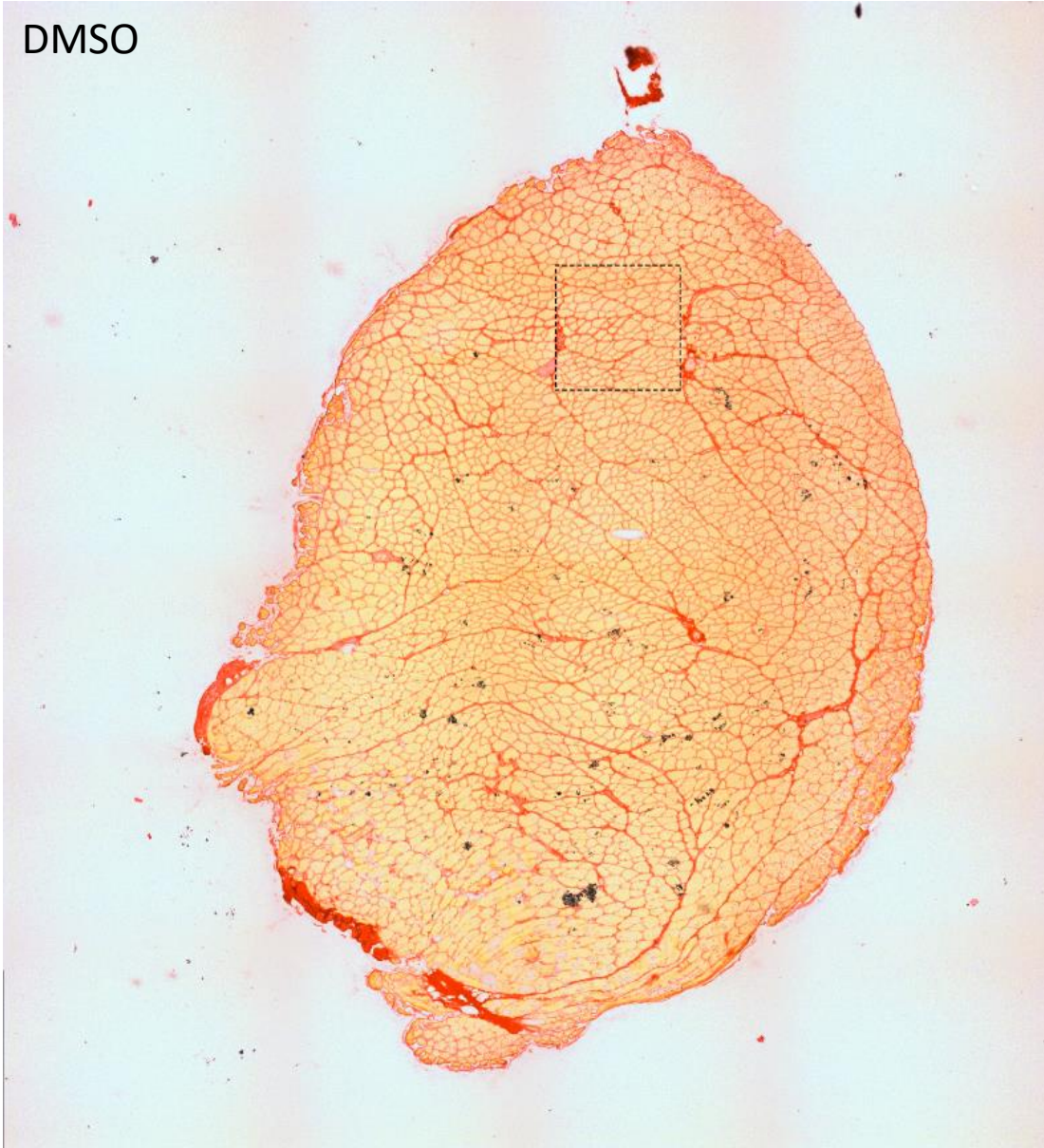

CA3

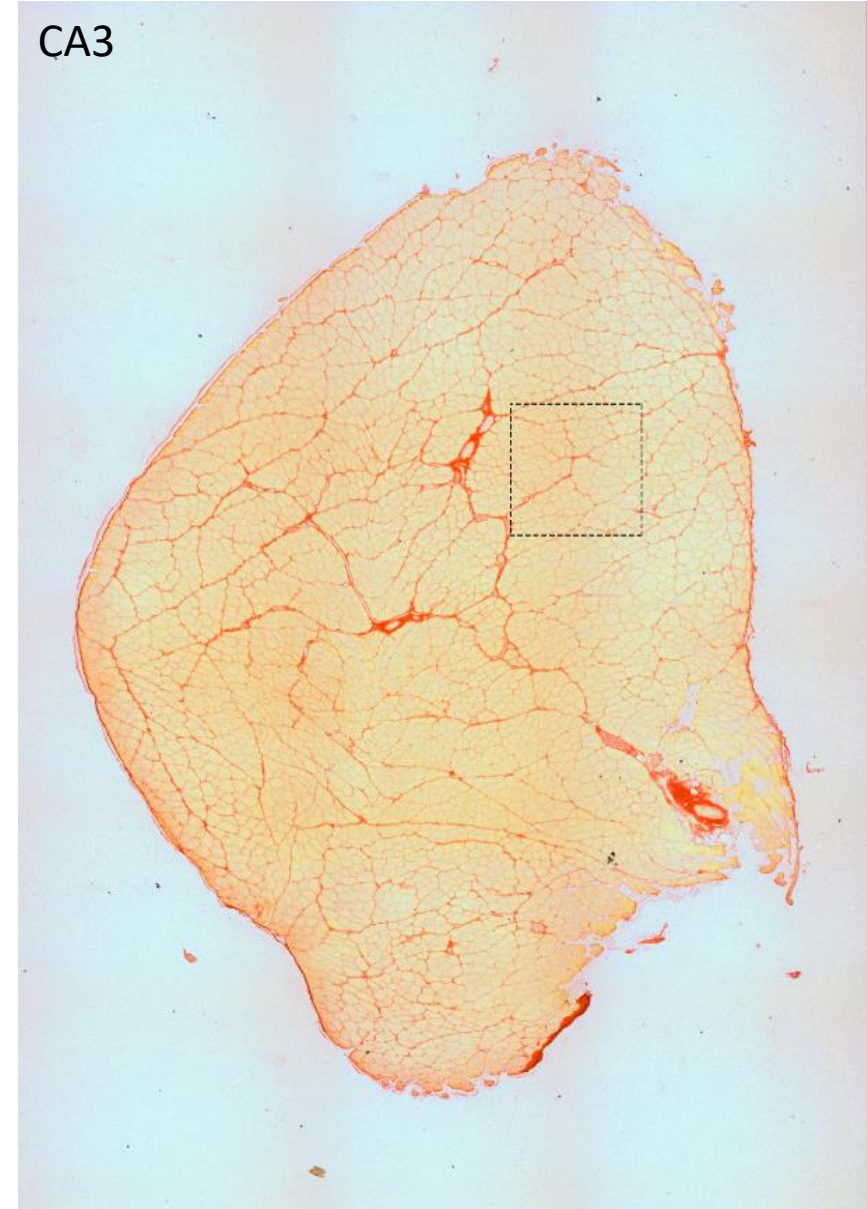

Supplement: Supplementary file 9 — Source Data for Figure 6 [file EMMM-15-e17187-s001.zip › Figure6_Source_Data/Figure6_IHC.pdf]

Figure 6 G

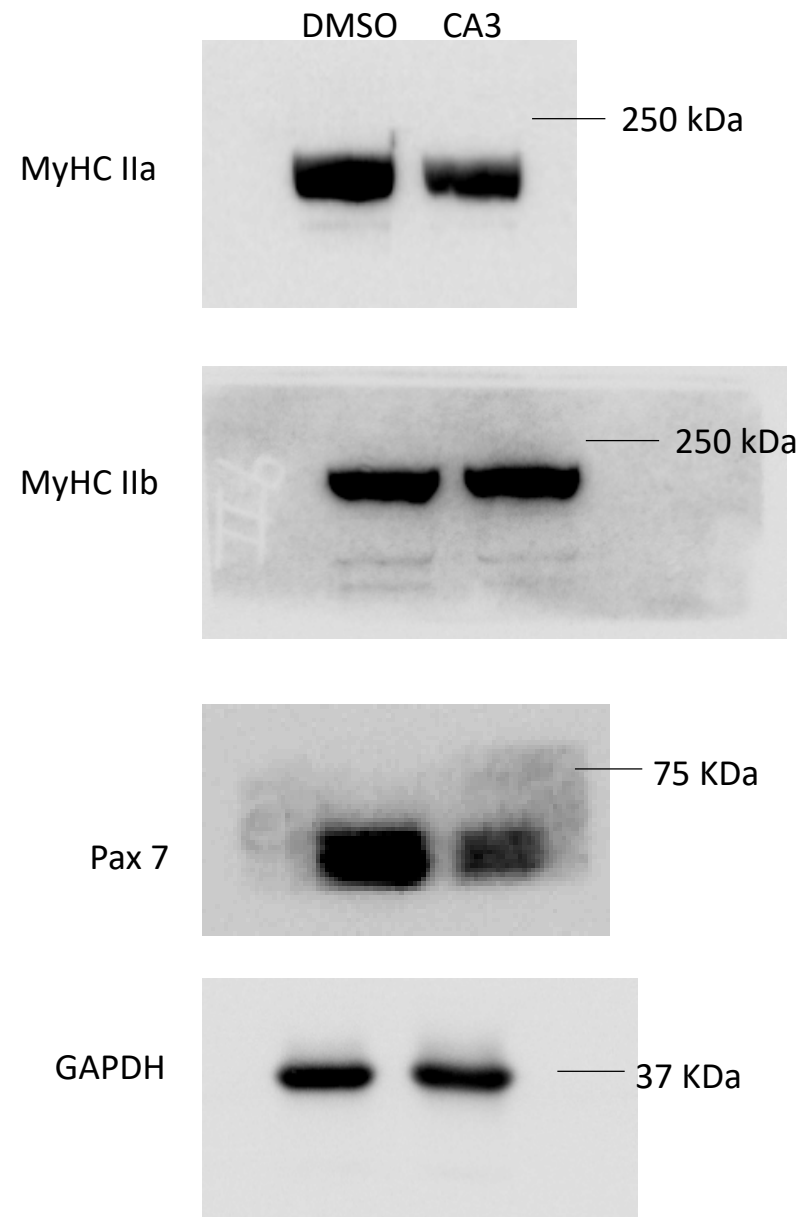

Figure 6 K

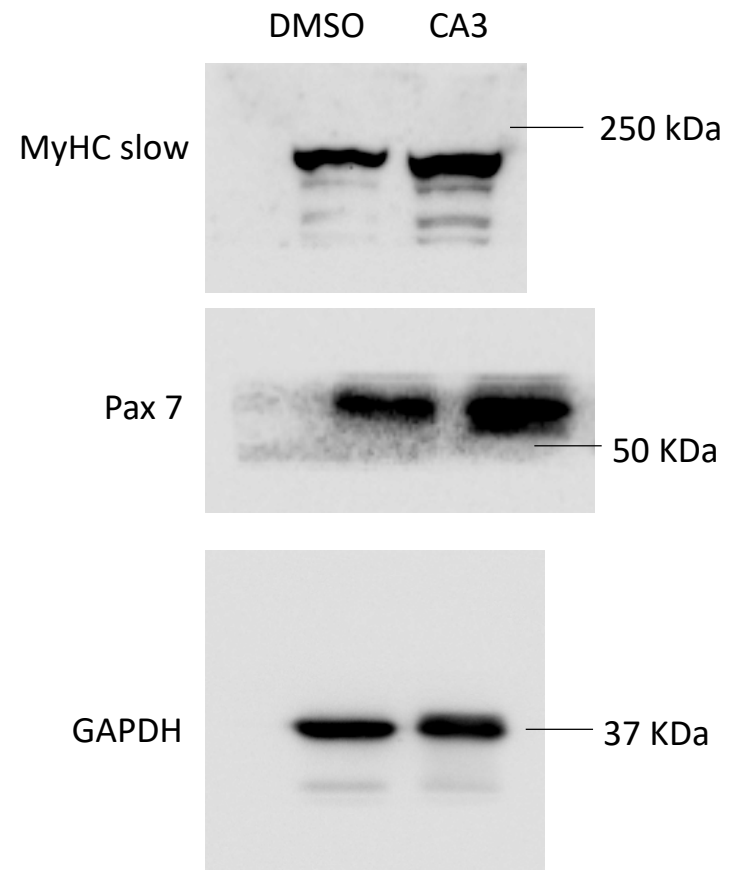

Figure 6 O

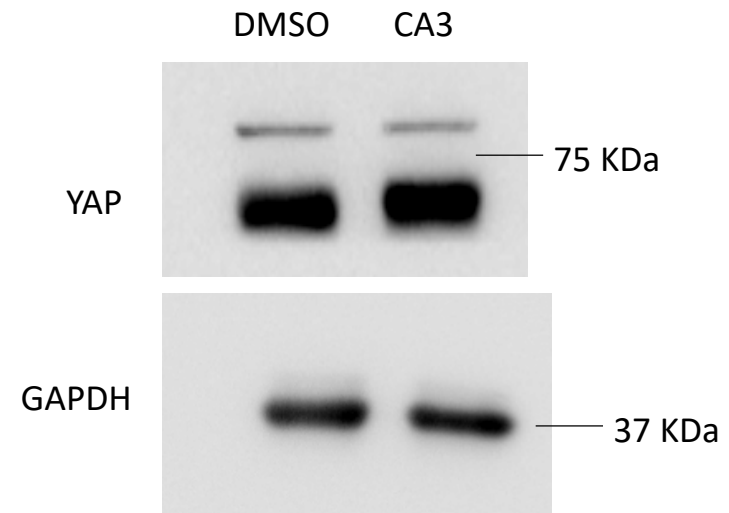

Figure 6 Q

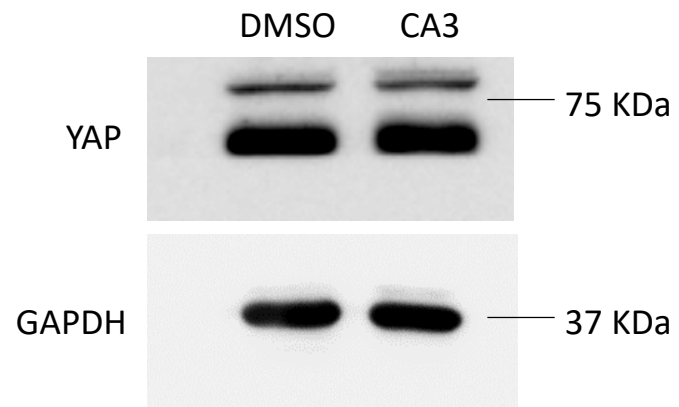

Figure 6 S

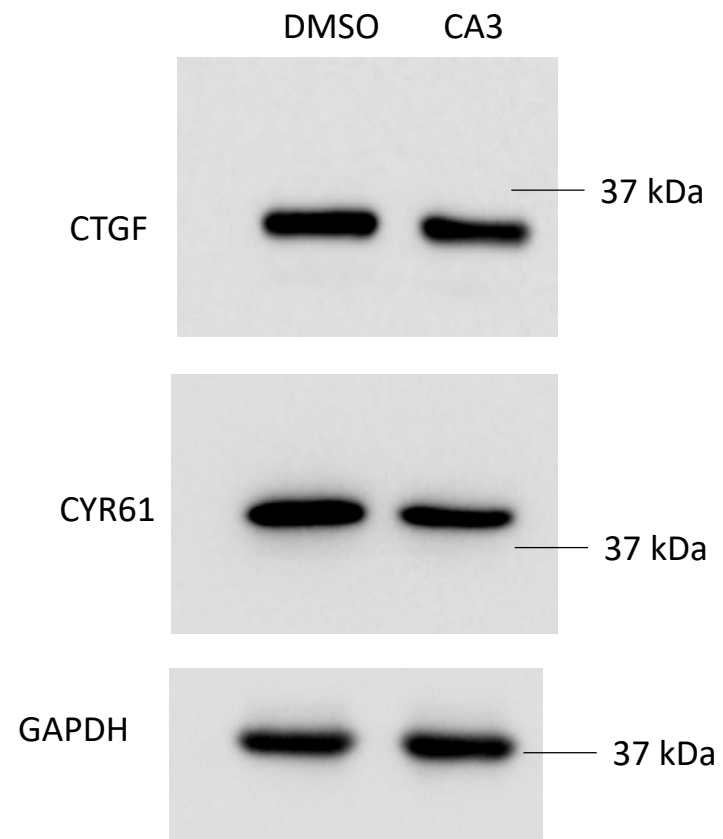

Figure 6U

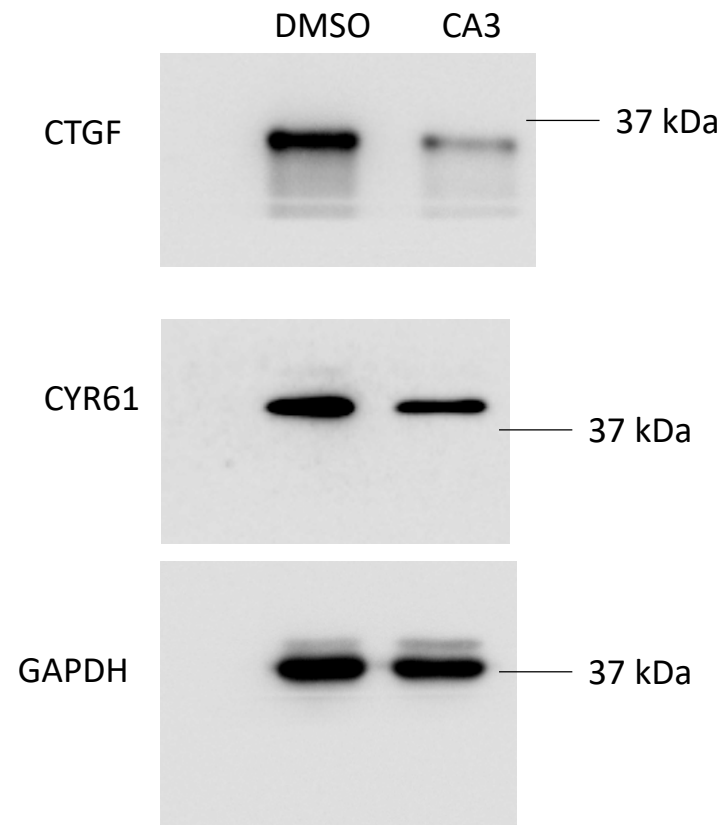

Supplement: Supplementary file 9 — Source Data for Figure 6 [file EMMM-15-e17187-s001.zip › Figure6_Source_Data/Figure6_Western blot.pdf]
