# Supplementary material for: Musculoskeletal defects associated with myosin heavy chain‐embryonic loss of function are mediated by the YAP signaling pathway
Source: EMBO Mol Med. 2023 Jul 26;15(9):e17187. doi: 10.15252/emmm.202217187 (PMC10493586; doi:10.15252/emmm.202217187)

Figure 2A-A’
MyHC IIb

8-10 weeks

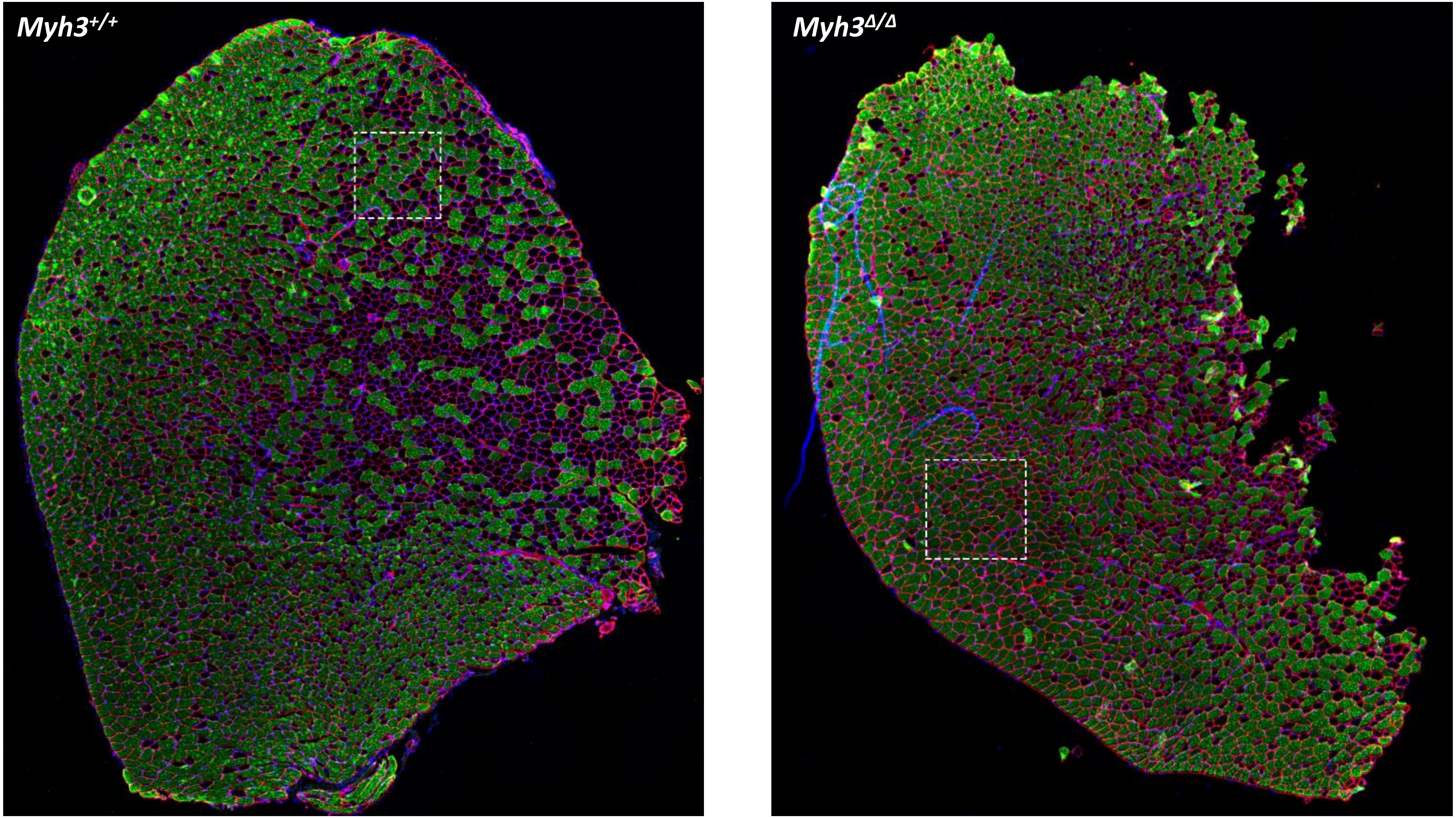

Figure 2B-B’
MyHC IIb

6 months

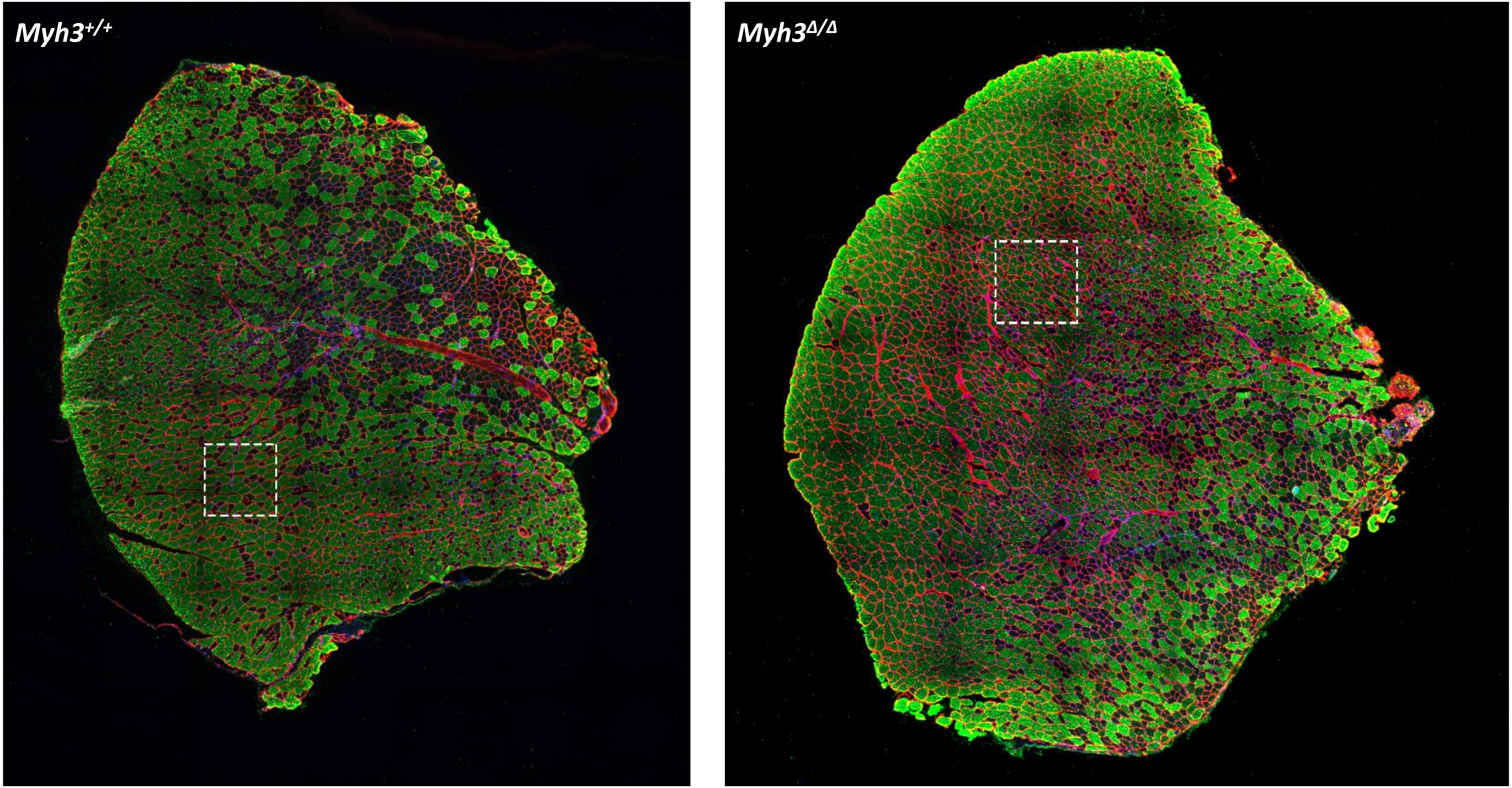

Figure 2E-E'
MyHC IIa

8-10 weeks

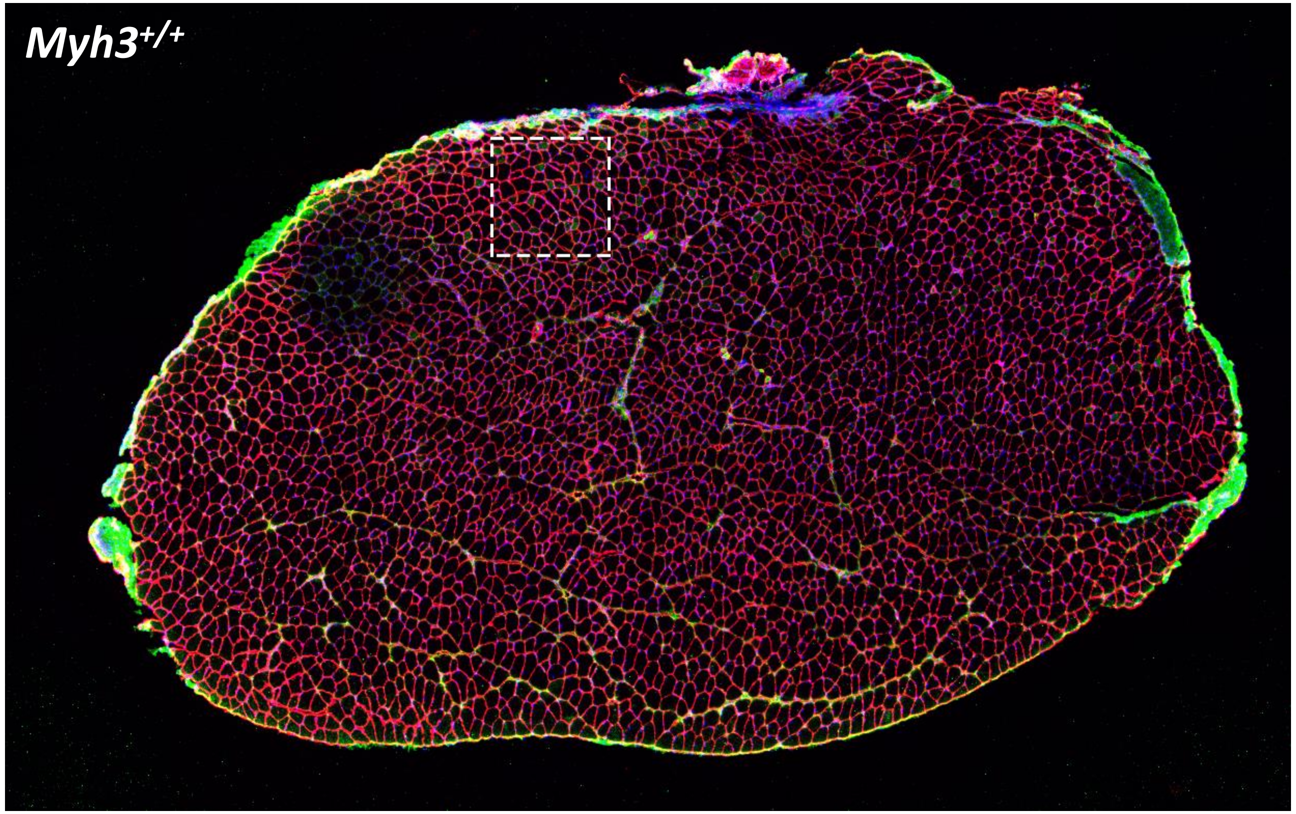

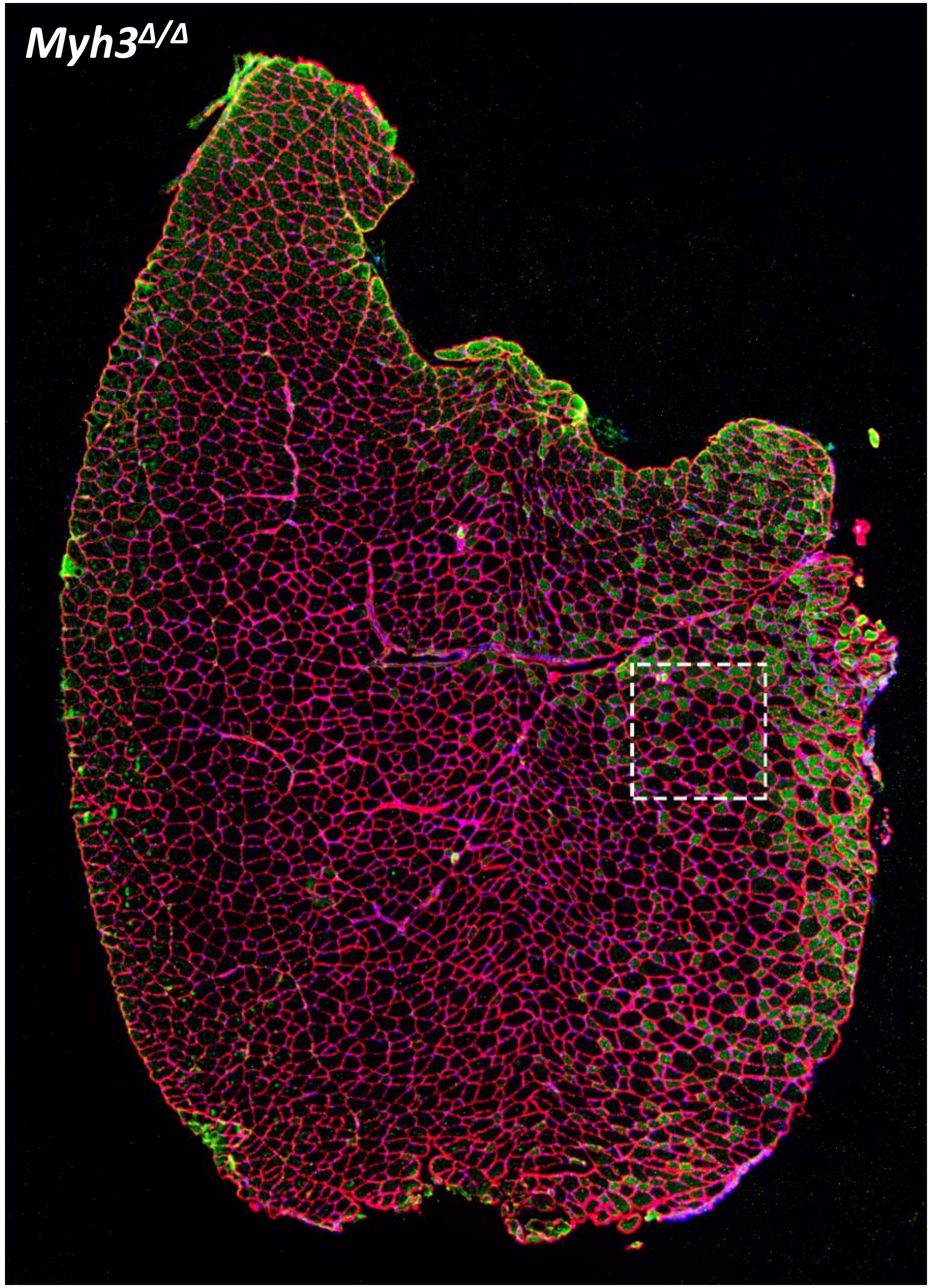

Figure 2F-F''
MyHC IIa

6 months

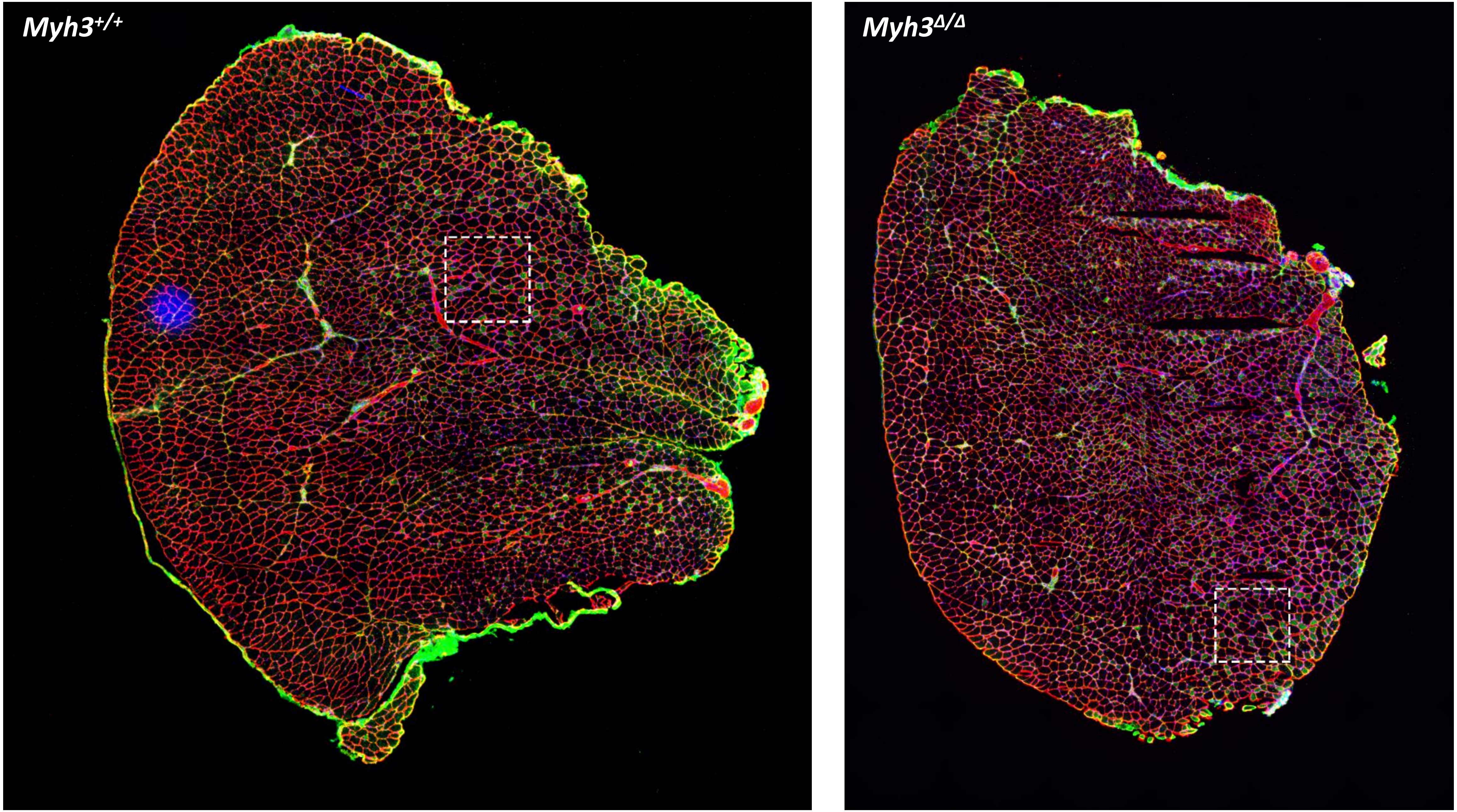

Figure 2I-I’
MyHC IIx

8-10 weeks

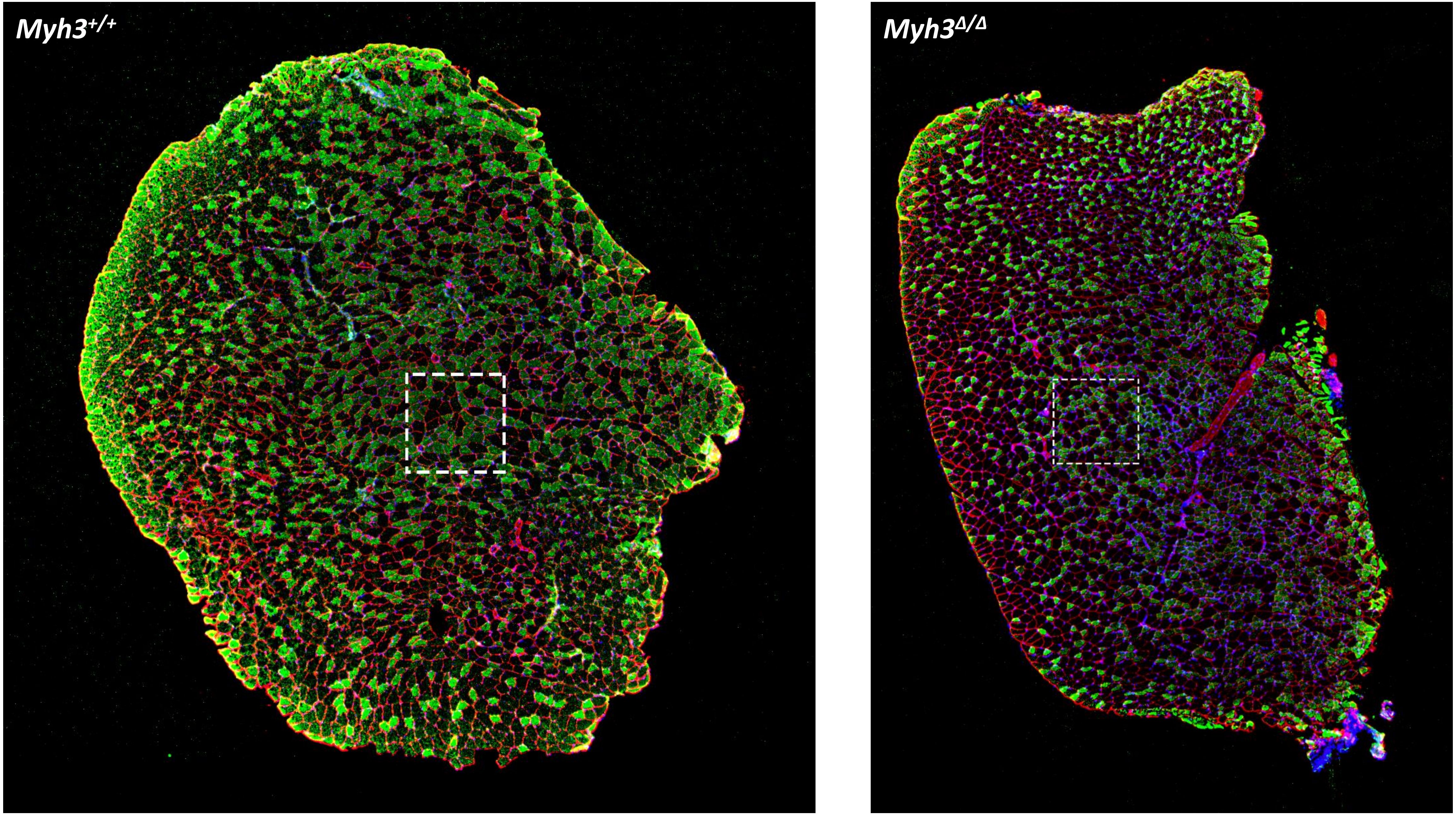

Figure 2J-J'
MyHC IIx

6 months

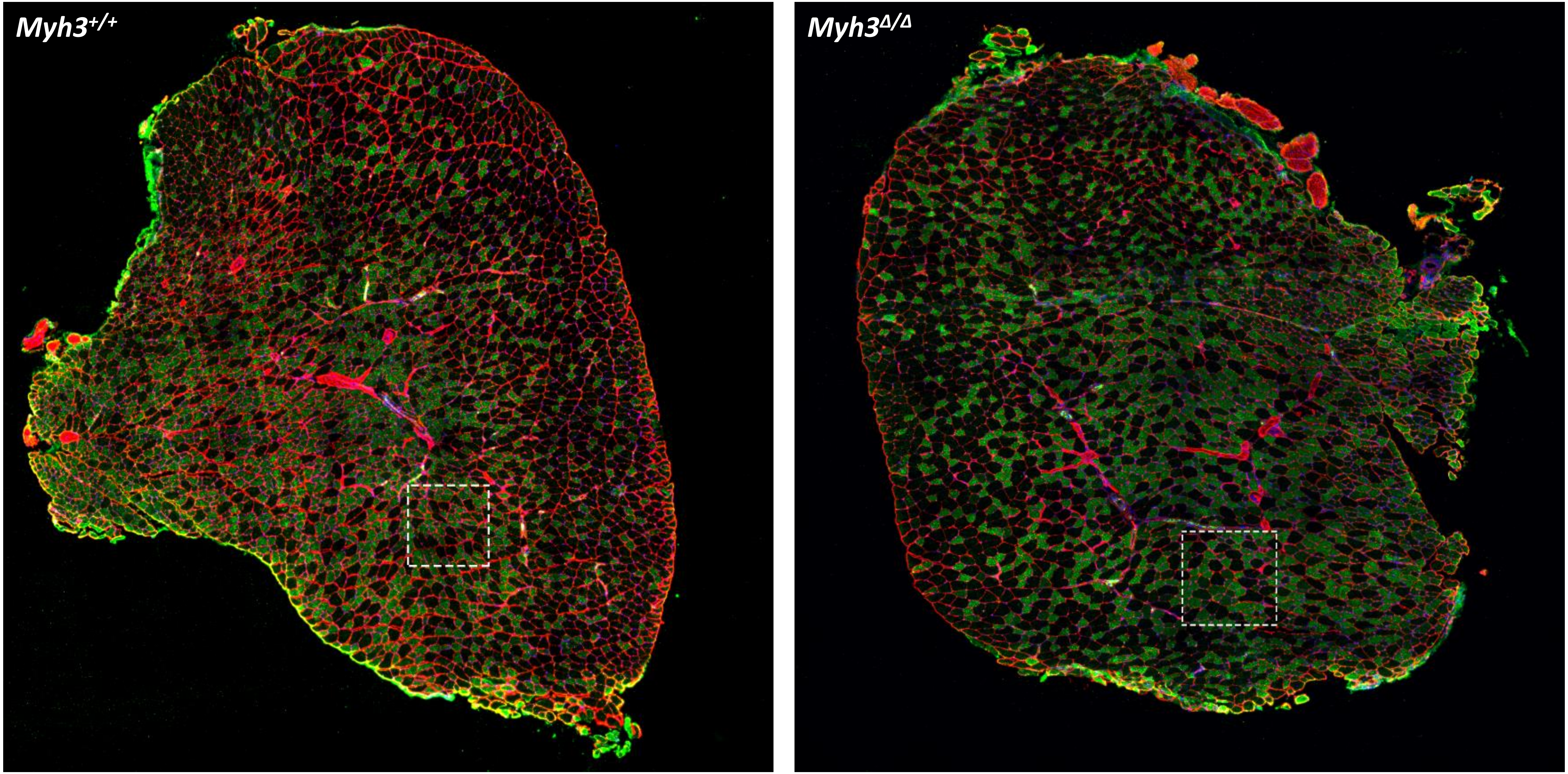

Figure 2M-M'
MyHC slow

8-10 weeks

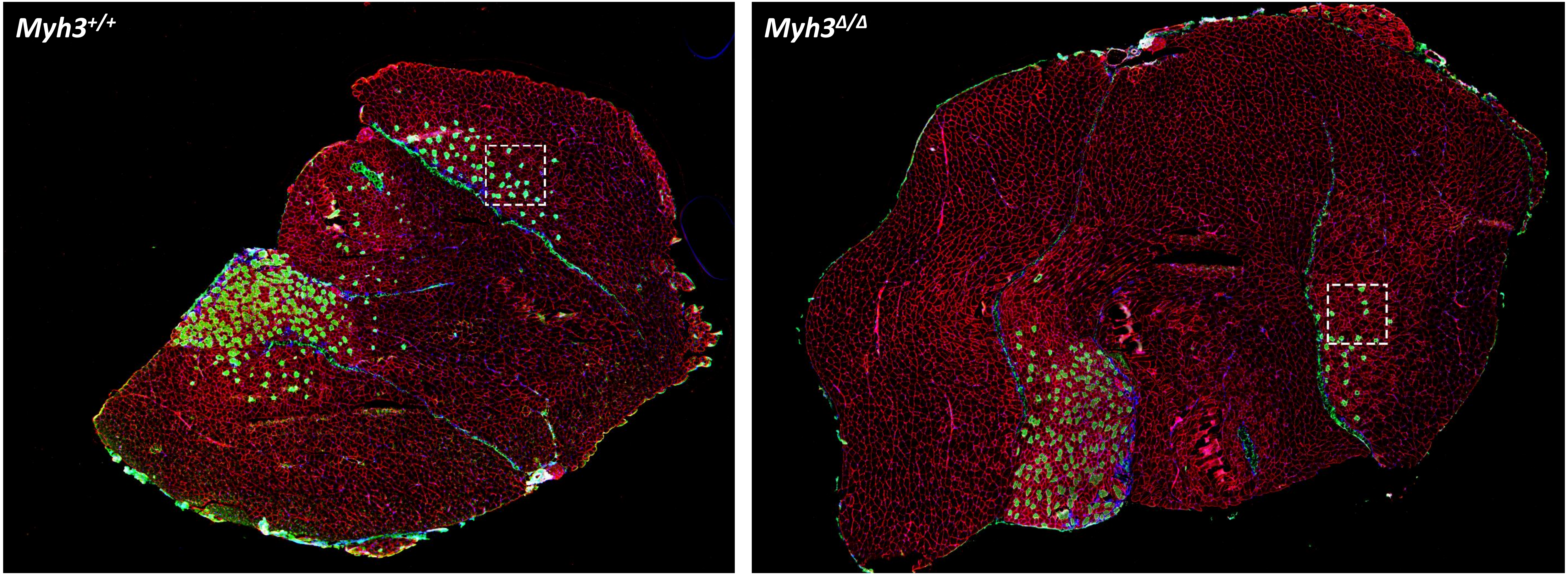

Figure 2N-N’

6 months

MyHC slow

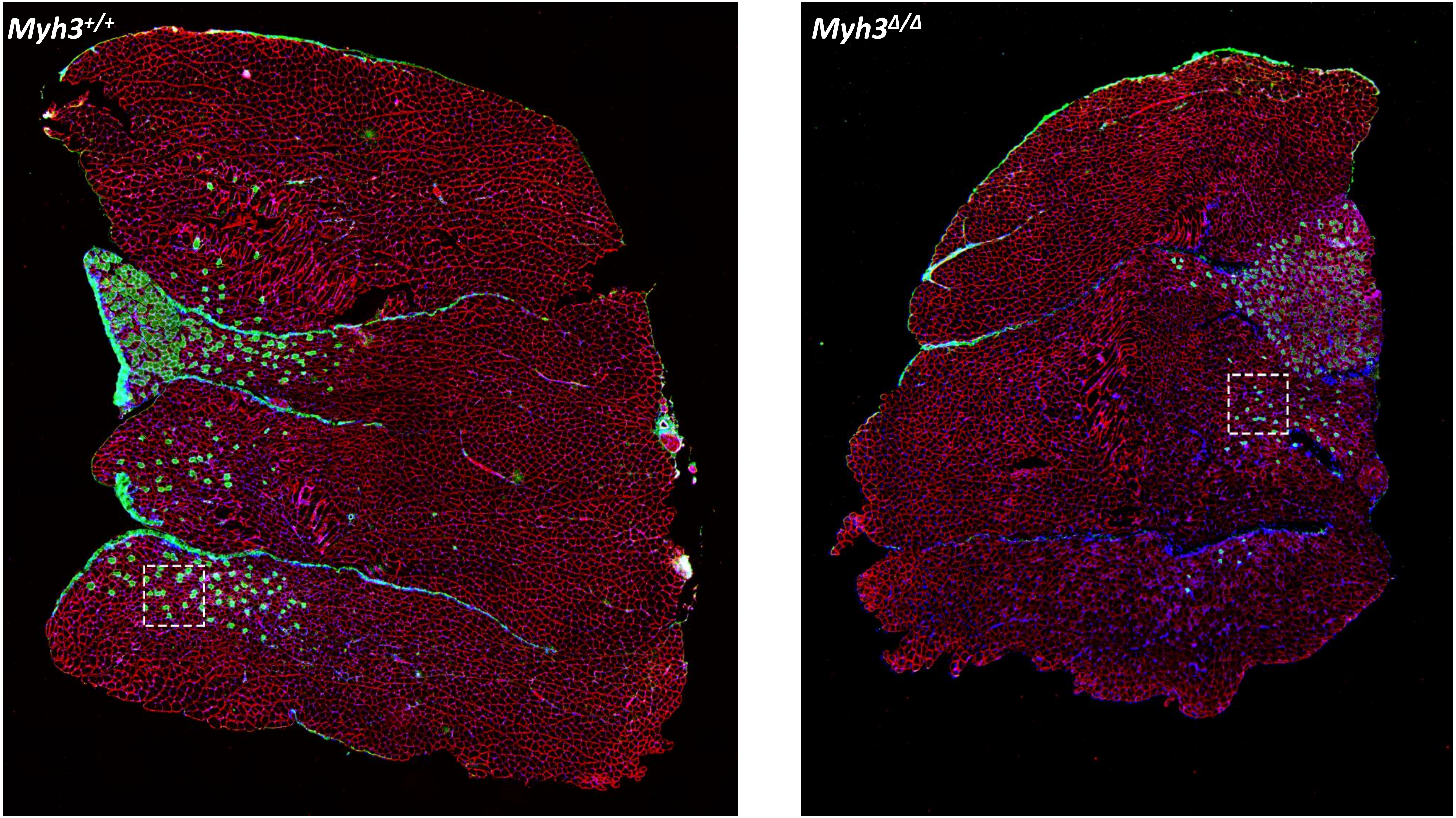

Supplement: Supplementary file 5 — Source Data for Figure 2 [file EMMM-15-e17187-s006.zip › Figure2_Source_Data/Figure2_IF.pdf]
