# Supplementary material for: Musculoskeletal defects associated with myosin heavy chain‐embryonic loss of function are mediated by the YAP signaling pathway
Source: EMBO Mol Med. 2023 Jul 26;15(9):e17187. doi: 10.15252/emmm.202217187 (PMC10493586; doi:10.15252/emmm.202217187)
Supplement: Supplementary file 5 — Source Data for Figure 2 [file EMMM-15-e17187-s006.zip › Figure2_Source_Data/Figure2_Western blot.pdf]

Figure Q

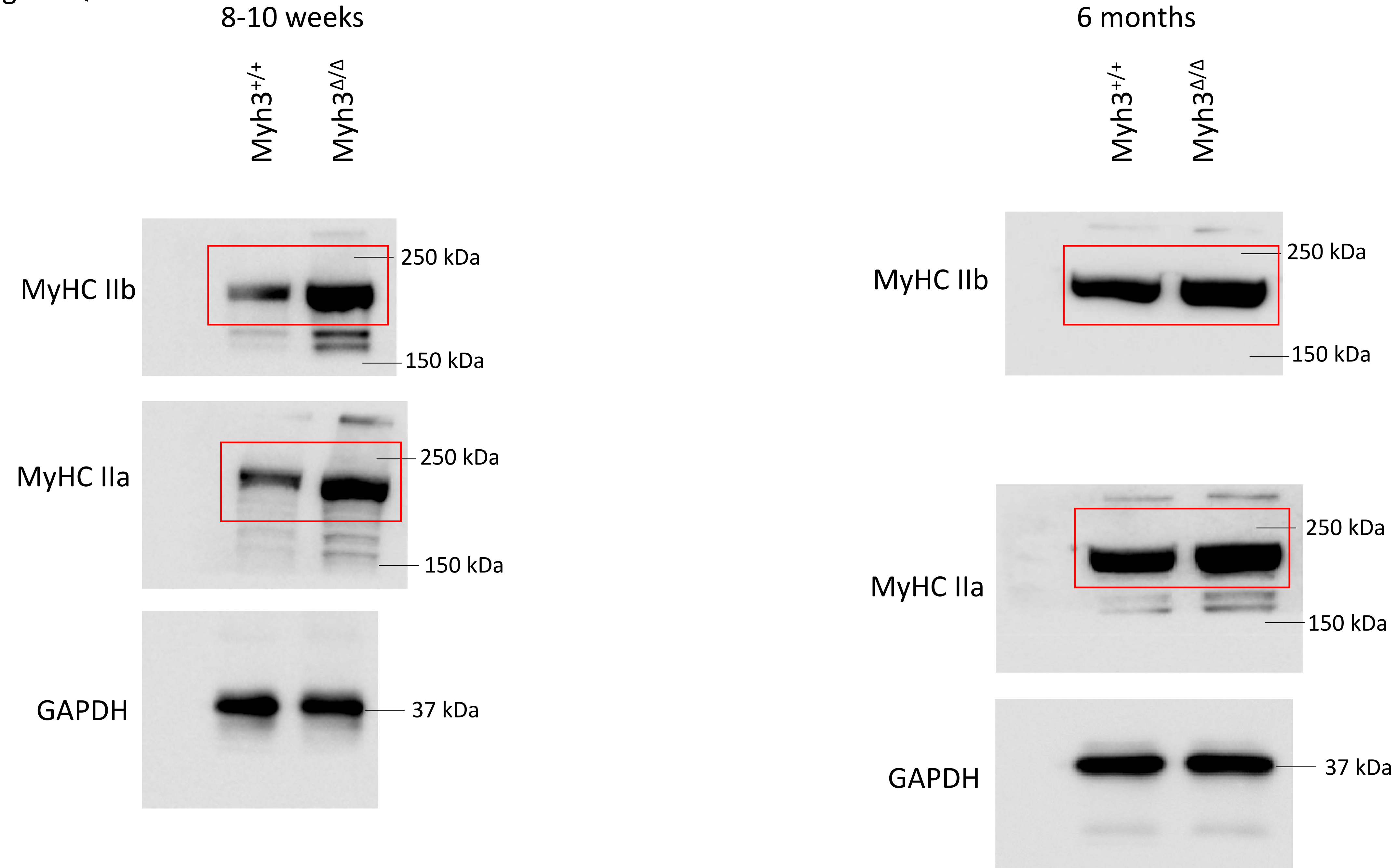

8-10 weeks
6 months
Myh3+/+
Myh3Δ/Δ
Myh3+/+
Myh3Δ/Δ
MyHC IIb
250 kDa
150 kDa
MyHC IIa
250 kDa
150 kDa
GAPDH
37 kDa
MyHC IIb
250 kDa
150 kDa
MyHC IIa
250 kDa
150 kDa
GAPDH
37 kDa

Figure V

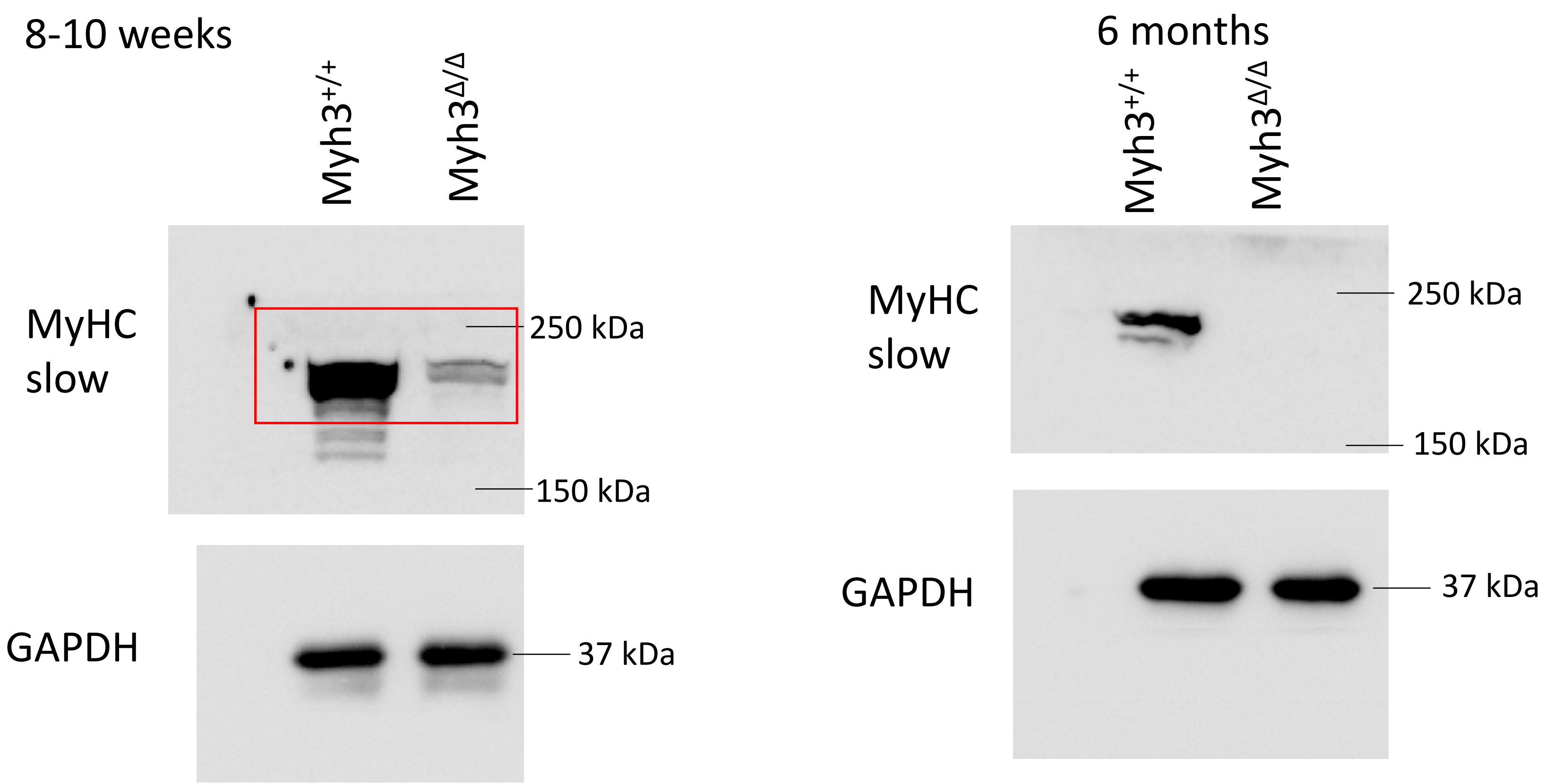
8-10 weeks
Myh3+/+
Myh3Δ/Δ
MyHC slow
250 kDa
150 kDa
GAPDH
37 kDa
6 months
Myh3+/+
Myh3Δ/Δ
MyHC slow
250 kDa
150 kDa
GAPDH
37 kDa
