# Supplementary material for: Musculoskeletal defects associated with myosin heavy chain‐embryonic loss of function are mediated by the YAP signaling pathway
Source: EMBO Mol Med. 2023 Jul 26;15(9):e17187. doi: 10.15252/emmm.202217187 (PMC10493586; doi:10.15252/emmm.202217187)

Figure 3A-B”

8-10 weeks

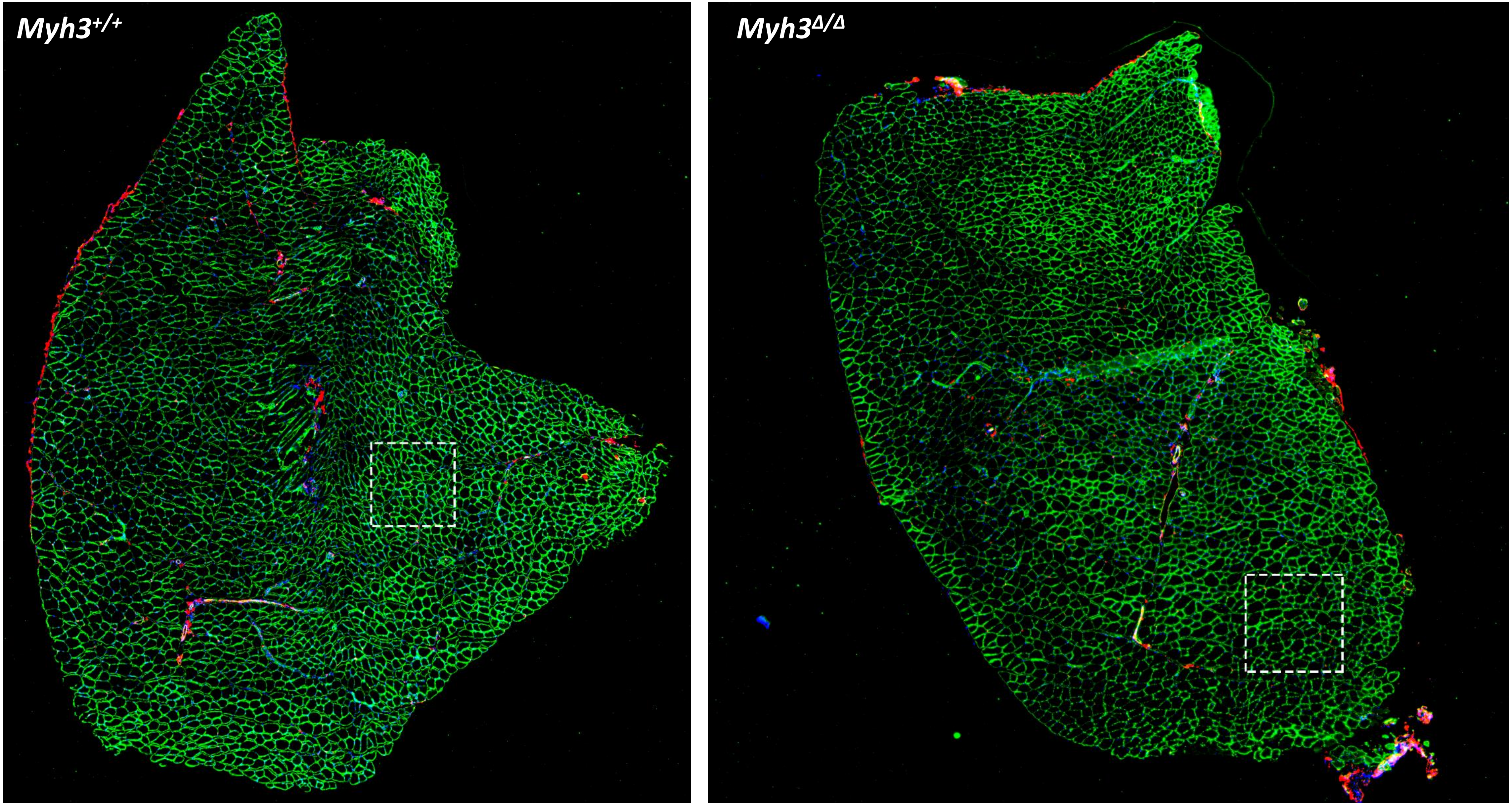

Figure 3D-E”

6 months

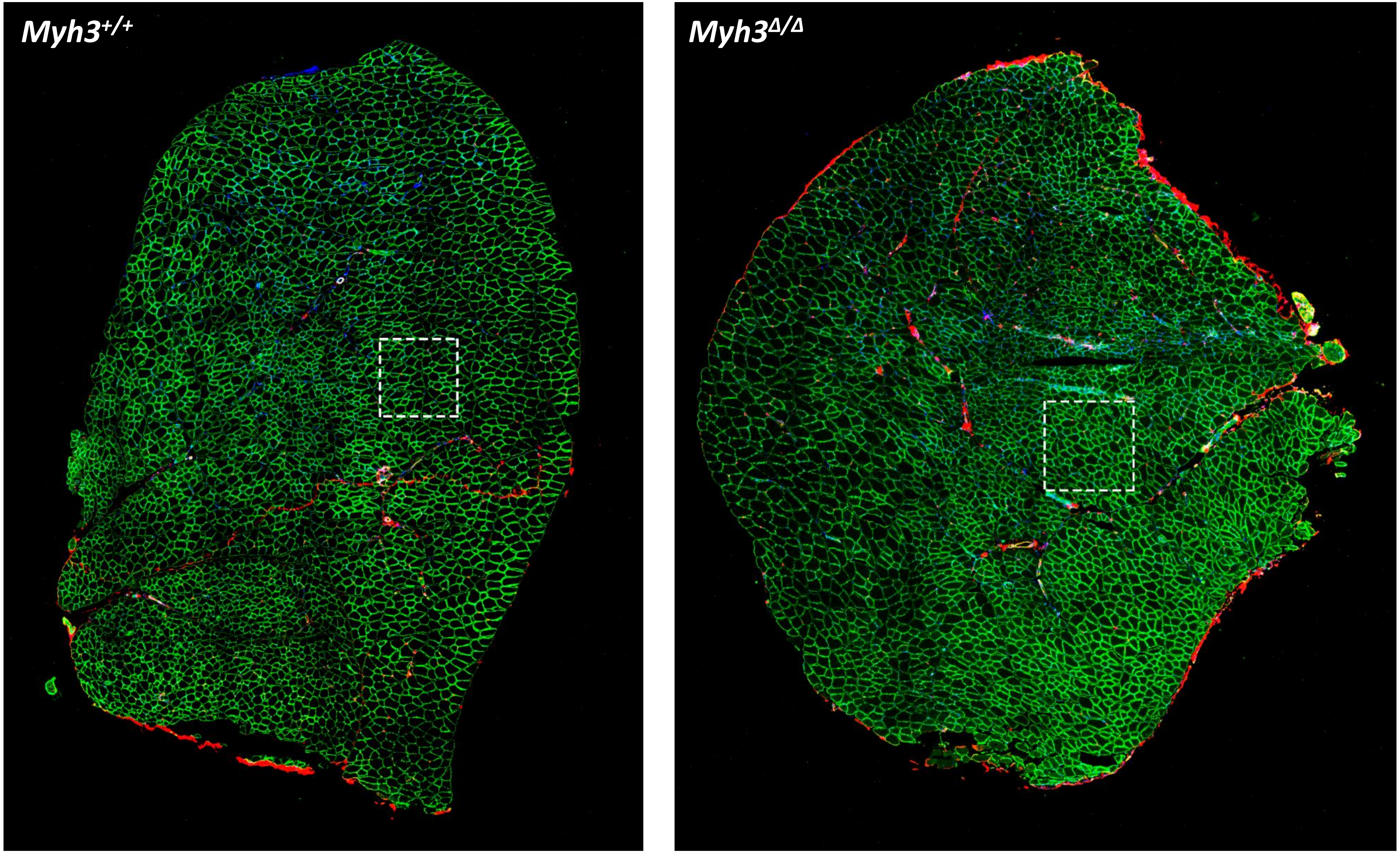

Figure N-O”

6 hrs

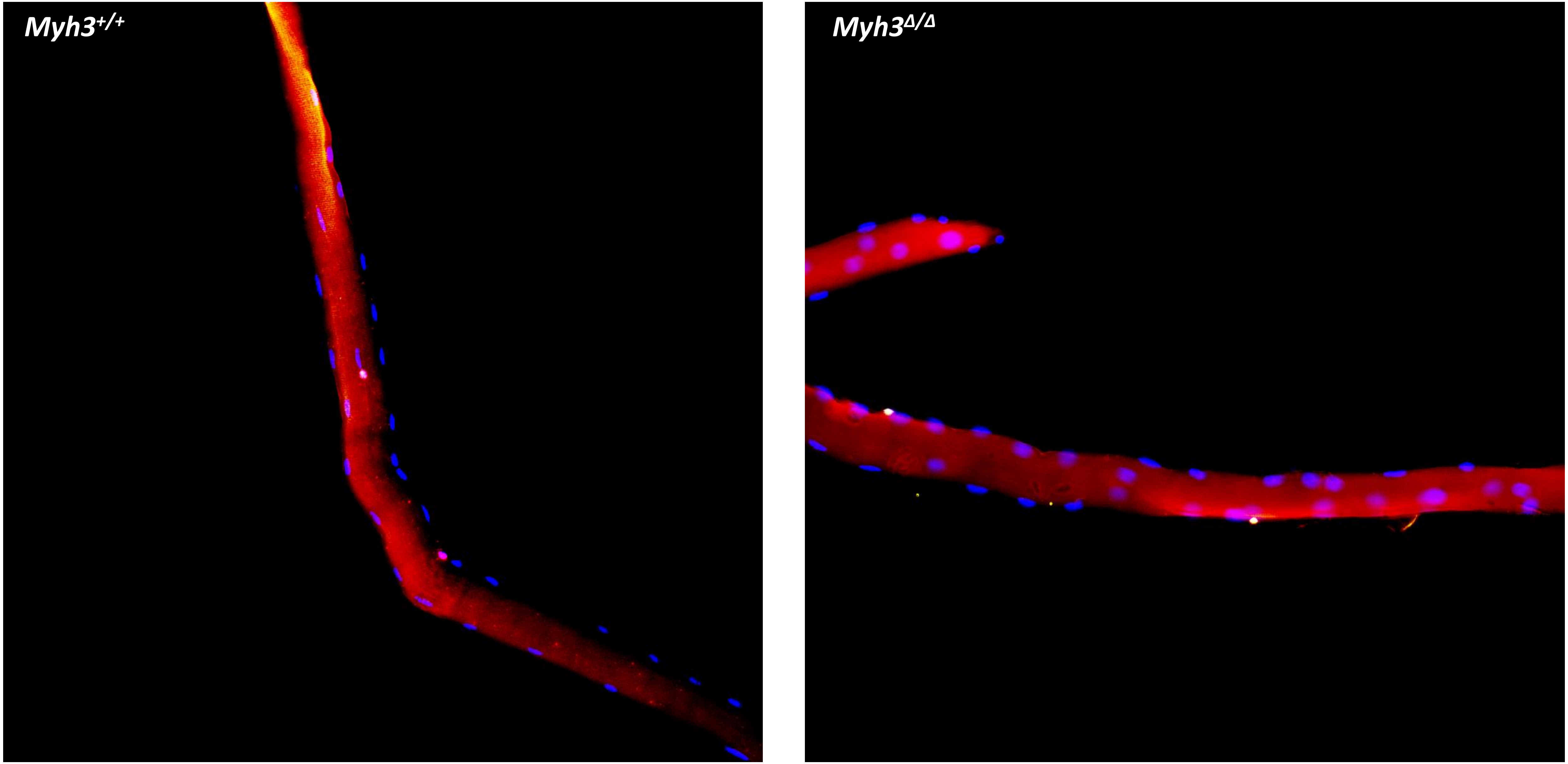

Figure Q-R

8-10 weeks

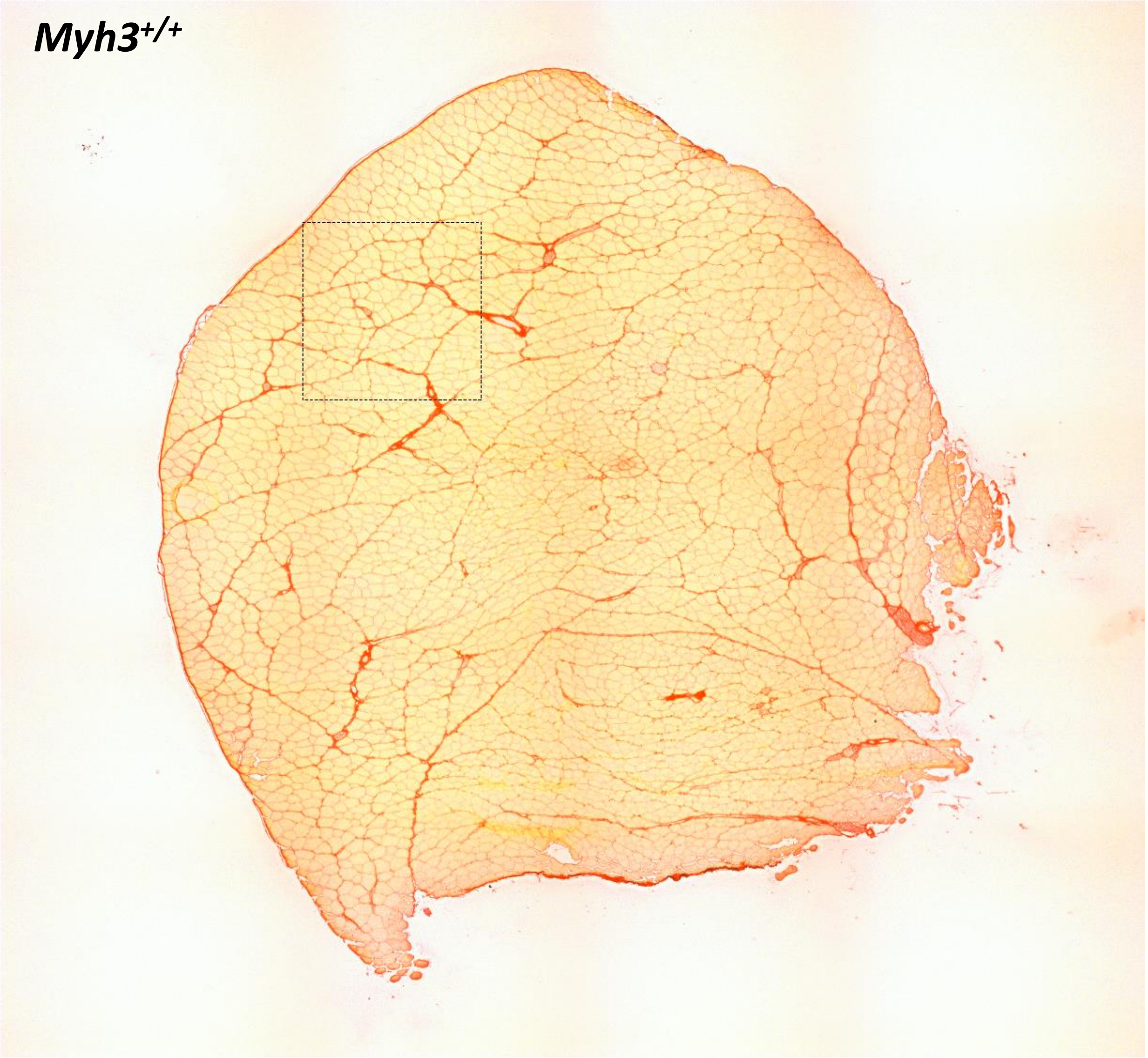

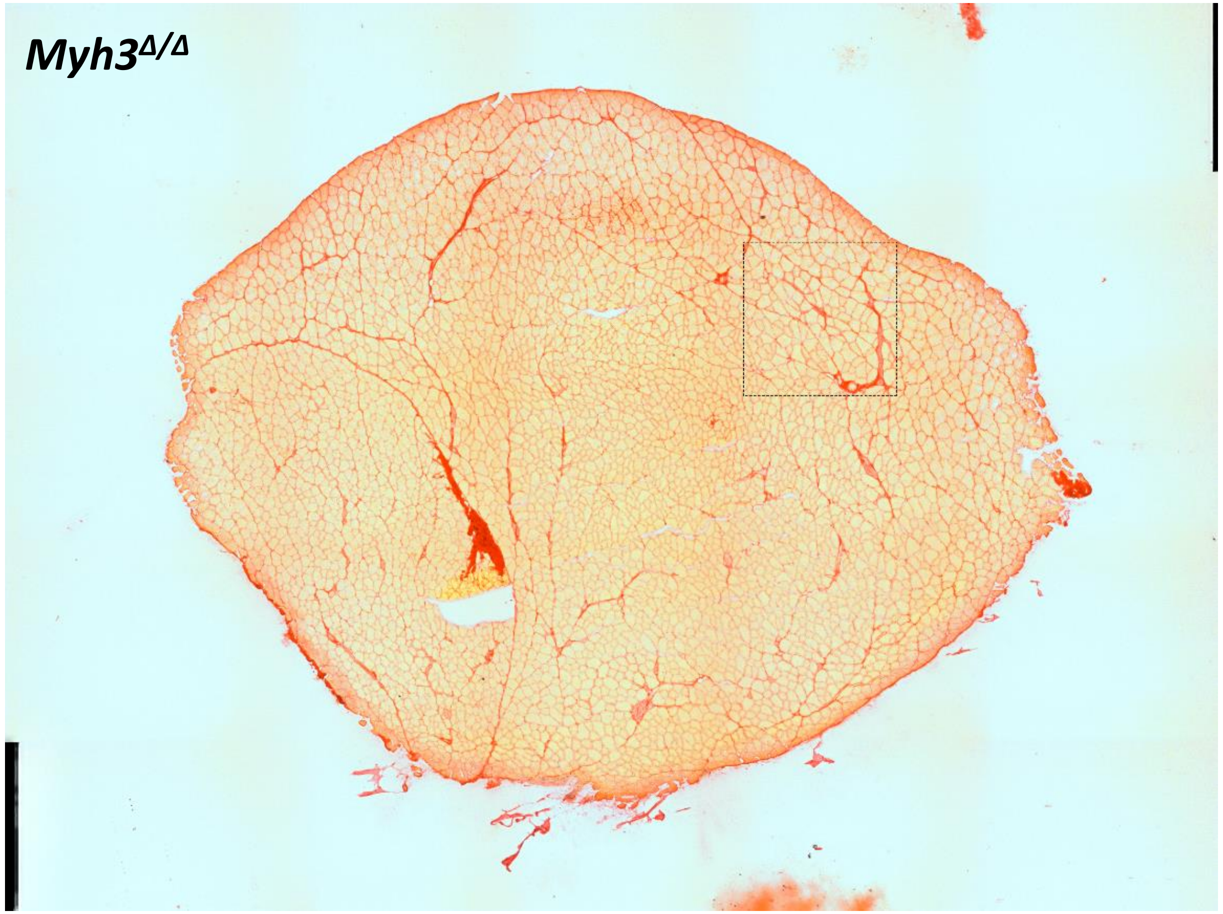

Figure T-U

6 months

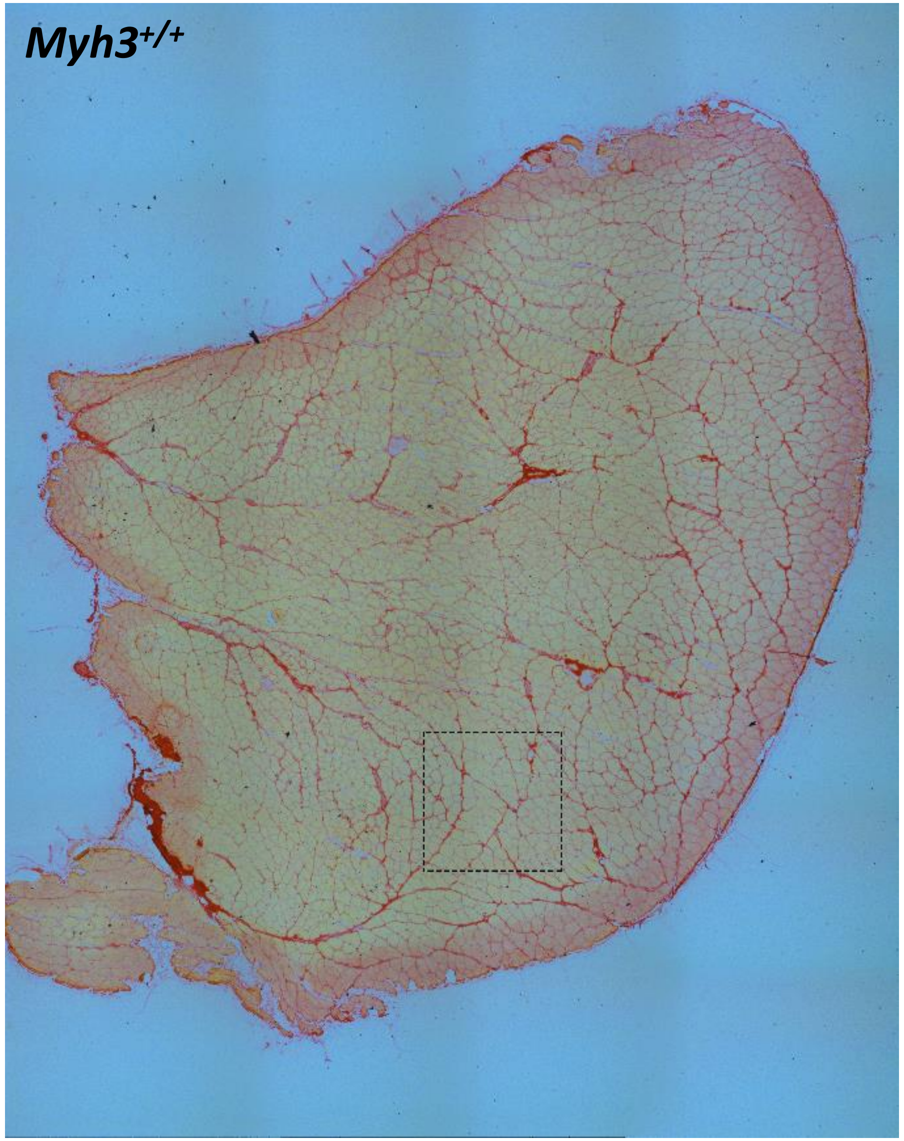

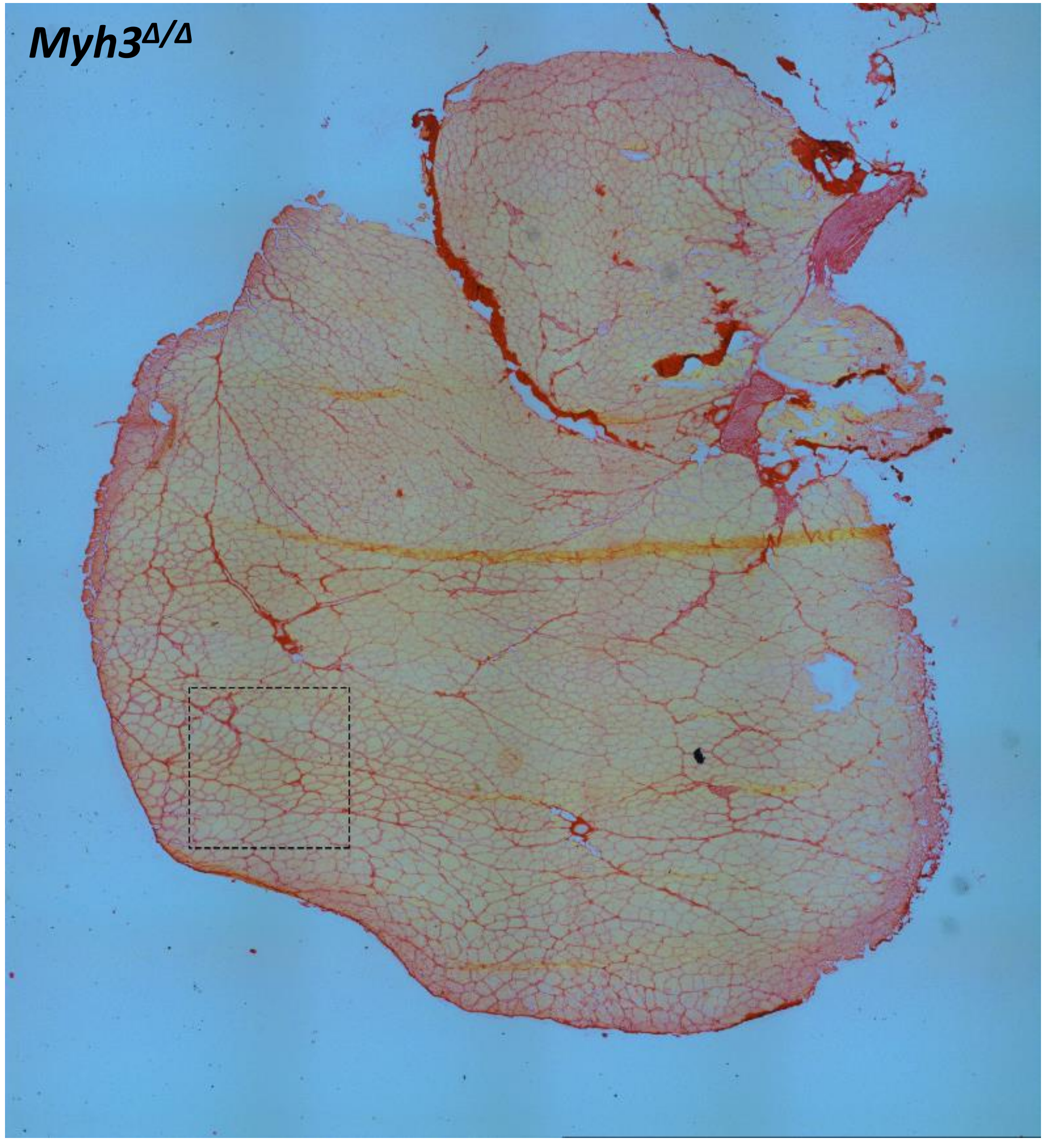

Supplement: Supplementary file 6 — Source Data for Figure 3 [file EMMM-15-e17187-s004.zip › Figure3_Source_Data_13072023/Figure3_IF.pdf]
