# Supplementary material for: Musculoskeletal defects associated with myosin heavy chain‐embryonic loss of function are mediated by the YAP signaling pathway
Source: EMBO Mol Med. 2023 Jul 26;15(9):e17187. doi: 10.15252/emmm.202217187 (PMC10493586; doi:10.15252/emmm.202217187)

Figure 3G

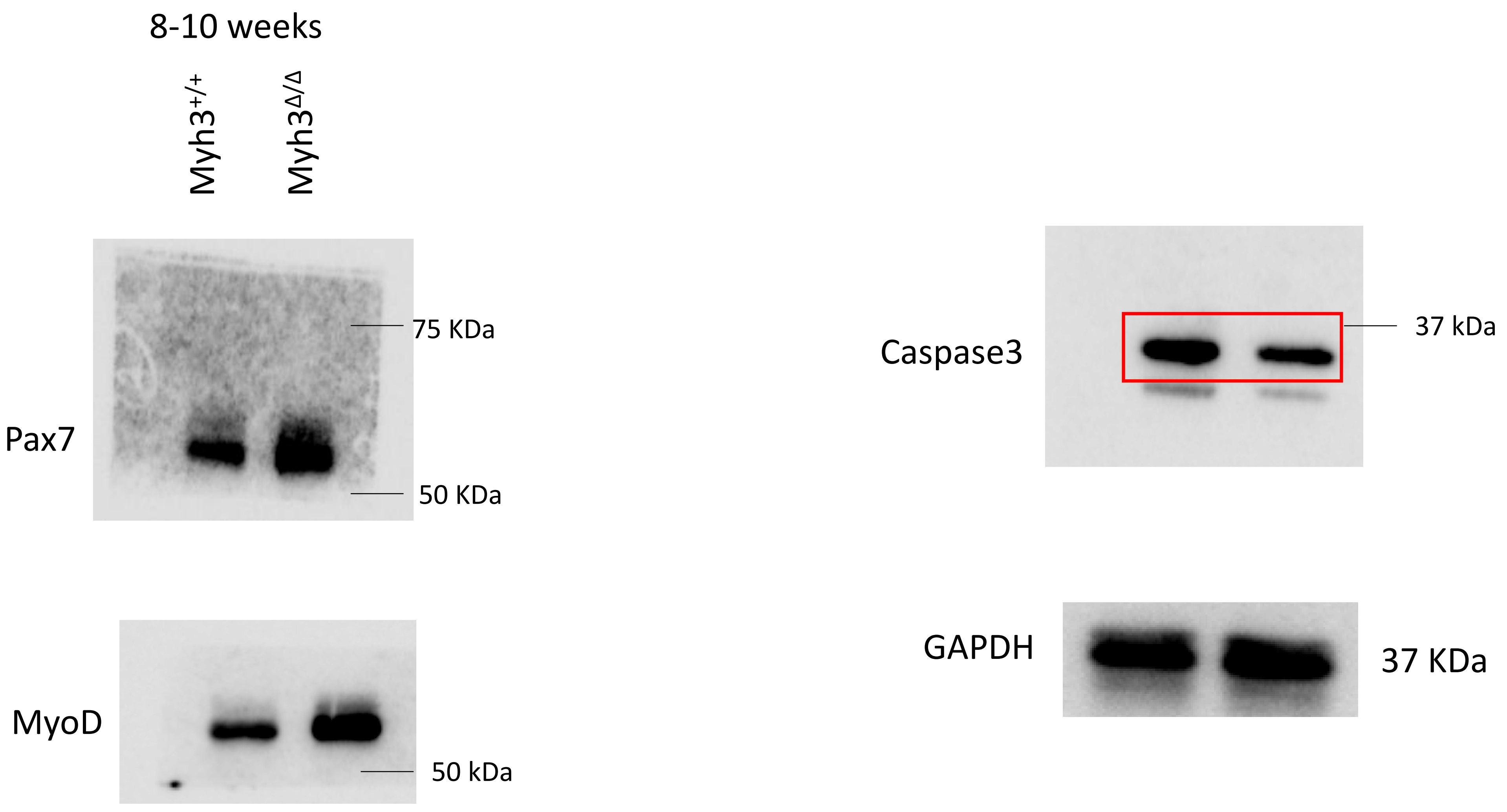

8-10 weeks
Myh3+/+
Myh3Δ/Δ
Pax7
75 KDa
50 KDa
MyoD
50 kDa
Caspase3
37 kDa
GAPDH
37 KDa

Figure 3G

6 months

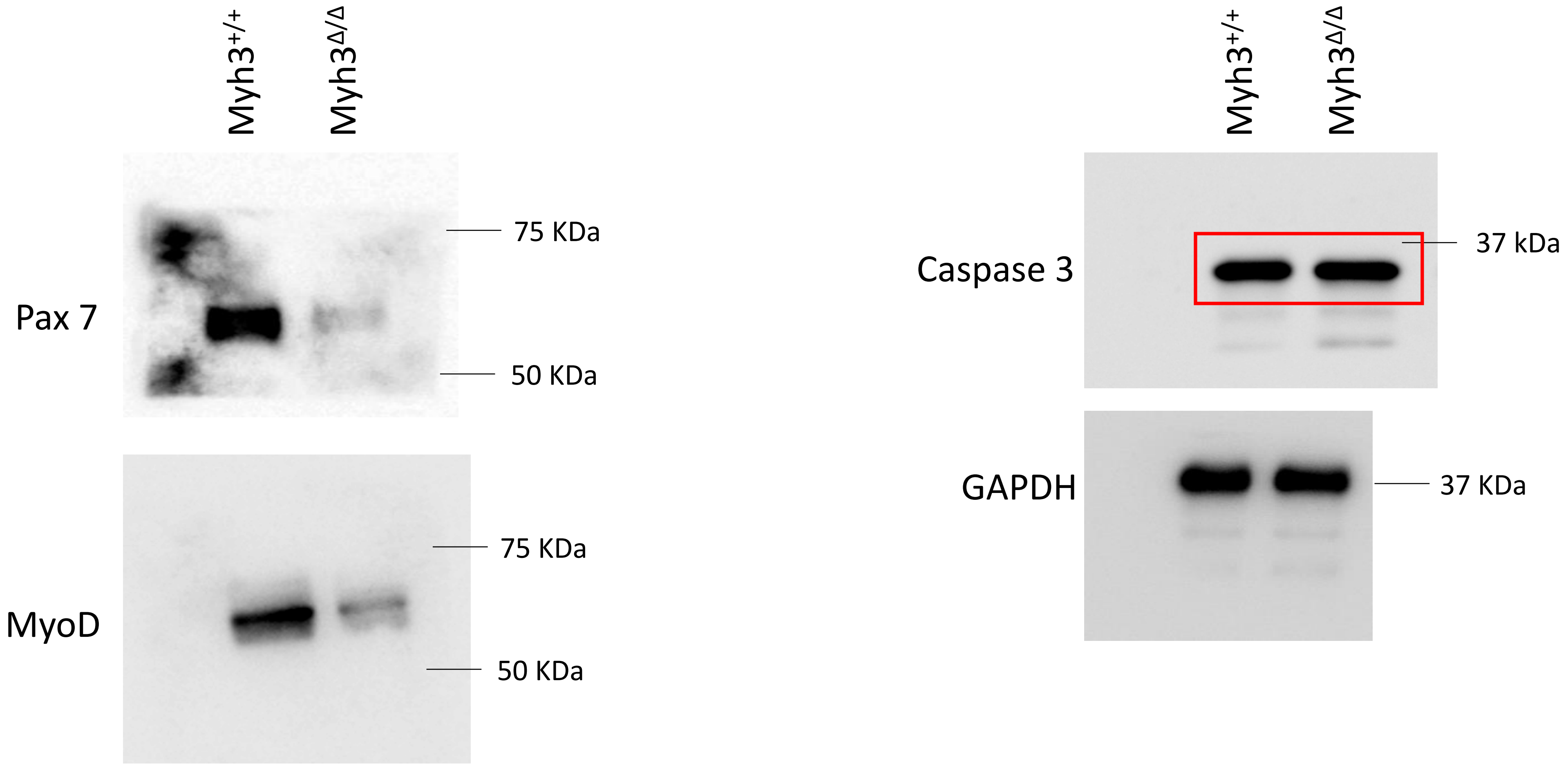

Supplement: Supplementary file 6 — Source Data for Figure 3 [file EMMM-15-e17187-s004.zip › Figure3_Source_Data_13072023/Figure3_Western blots.pdf]
