# Supplementary material for: Musculoskeletal defects associated with myosin heavy chain‐embryonic loss of function are mediated by the YAP signaling pathway
Source: EMBO Mol Med. 2023 Jul 26;15(9):e17187. doi: 10.15252/emmm.202217187 (PMC10493586; doi:10.15252/emmm.202217187)
Supplement: Supplementary file 10 [file EMMM-15-e17187-s007.pdf]

EMBO Molecular Medicine

Musculoskeletal defects associated with Myh3 loss of function are mediated by YAP signaling

Anushree Bharadwaj, Jaydeep Sharma, Jagriti Singh, Mahima Kumari, Tanushri Dargar, Bhargab Kalita, and Sam Mathew
DOI: 10.15252/emmm.202217187

Corresponding author(s): Sam Mathew (sjmathew@rcb.res.in)

Review Timeline:

Submission Date:	16th Nov 22
Editorial Decision:	9th Dec 22
Revision Received:	30th May 23
Editorial Decision:	2nd Jul 23
Revision Received:	13th Jul 23
Accepted:	17th Jul 23

Editor: Zeljko Durdevic

Transaction Report:

(Note: With the exception of the correction of typographical or spelling errors that could be a source of ambiguity, letters and reports are not edited. Depending on transfer agreements, referee reports obtained elsewhere may or may not be included in this compilation. Referee reports are anonymous unless the Referee chooses to sign their reports.)

9th Dec 2022

Dear Dr. Mathew,

Thank you for the submission of your manuscript to EMBO Molecular Medicine. We have now received feedback from the three reviewers who agreed to evaluate your manuscript. As you will see from the reports below, the referee #2 is overall supportive raising important but minor critique, referee #3 highlights the novelty of the study raising important concerns particularly regarding the proposed mechanism, while the referee #1 recognizes interest of the study but also raises serious concerns, particularly regarding the incremental conceptual advance of the study. During our cross-commenting session referees agreed that all the concerns should be addressed in a major revision and that the focus of the revision should be in providing more mechanistic insight, better characterization of the mouse model in the disease context and better highlighting the advance and impact of the study for the field. If you would like to discuss further the points raised by the referees, I am available to do so via email or video. Let me know if you are interested in this option.

Further consideration of a revision that addresses reviewers' concerns in full will entail a second round of review. EMBO Molecular Medicine encourages a single round of revision only and therefore, acceptance or rejection of the manuscript will depend on the completeness of your responses included in the next, final version of the manuscript. For this reason, and to save you from any frustrations in the end, I would strongly advise against returning an incomplete revision.

We would welcome the submission of a revised version within three months for further consideration. Please let us know if you require longer to complete the revision.

I look forward to receiving your revised manuscript.

Yours sincerely,

Zeljko Durdevic

Zeljko Durdevic
Editor
EMBO Molecular Medicine

*****

When submitting your revised manuscript, please carefully review the instructions that follow below. We perform an initial quality control of all revised manuscripts before re-review; failure to include requested items will delay the evaluation of your revision.

We require:

1) A .docx formatted version of the manuscript text (including legends for main figures, EV figures and tables). Please make sure that the changes are highlighted to be clearly visible.

2) Individual production quality figure files as .eps, .tif, .jpg (one file per figure). For guidance, download the 'Figure Guide PDF': (https://www.embopress.org/page/journal/17574684/authorguide#figureformat).

3) A .docx formatted letter INCLUDING the reviewers' reports and your detailed point-by-point responses to their comments. As part of the EMBO Press transparent editorial process, the point-by-point response is part of the Review Process File (RPF), which will be published alongside your paper.

4) A complete author checklist, which you can download from our author guidelines (https://www.embopress.org/page/journal/17574684/authorguide#submissionofrevisions). Please insert information in the checklist that is also reflected in the manuscript. The completed author checklist will also be part of the RPF.

5) Please note that all corresponding authors are required to supply an ORCID ID for their name upon submission of a revised manuscript.

6) It is mandatory to include a 'Data Availability' section after the Materials and Methods. Before submitting your revision, primary datasets produced in this study need to be deposited in an appropriate public database, and the accession numbers and database listed under 'Data Availability'. Please remember to provide a reviewer password if the datasets are not yet public (see https://www.embopress.org/page/journal/17574684/authorguide#dataavailability).

In case you have no data that requires deposition in a public database, please state so in this section. Note that the Data Availability Section is restricted to new primary data that are part of this study.

7) For data quantification: please specify the name of the statistical test used to generate error bars and P values, the number (n) of independent experiments (specify technical or biological replicates) underlying each data point and the test used to calculate p-values in each figure legend. The figure legends should contain a basic description of n, P and the test applied. Graphs must include a description of the bars and the error bars (s.d., s.e.m.). See also 'Figure Legend' guidelines: https://www.embopress.org/page/journal/17574684/authorguide#figureformat

8) At EMBO Press we ask authors to provide source data for the main manuscript figures. Our source data coordinator will contact you to discuss which figure panels we would need source data for and will also provide you with helpful tips on how to upload and organize the files.

9) Our journal encourages inclusion of *data citations in the reference list* to directly cite datasets that were re-used and obtained from public databases. Data citations in the article text are distinct from normal bibliographical citations and should directly link to the database records from which the data can be accessed. In the main text, data citations are formatted as follows: "Data ref: Smith et al, 2001" or "Data ref: NCBI Sequence Read Archive PRJNA342805, 2017". In the Reference list, data citations must be labeled with "[DATASET]". A data reference must provide the database name, accession number/identifiers and a resolvable link to the landing page from which the data can be accessed at the end of the reference. Further instructions are available at .

10) We replaced Supplementary Information with Expanded View (EV) Figures and Tables that are collapsible/expandable online. A maximum of 5 EV Figures can be typeset. EV Figures should be cited as 'Figure EV1, Figure EV2" etc... in the text and their respective legends should be included in the main text after the legends of regular figures.

- For the figures that you do NOT wish to display as Expanded View figures, they should be bundled together with their legends in a single PDF file called *Appendix*, which should start with a short Table of Content. Appendix figures should be referred to in the main text as: "Appendix Figure S1, Appendix Figure S2" etc.

- Additional Tables/Datasets should be labeled and referred to as Table EV1, Dataset EV1, etc. Legends have to be provided in a separate tab in case of .xls files. Alternatively, the legend can be supplied as a separate text file (README) and zipped together with the Table/Dataset file.

See detailed instructions here:

.

11) The paper explained: EMBO Molecular Medicine articles are accompanied by a summary of the articles to emphasize the major findings in the paper and their medical implications for the non-specialist reader. Please provide a draft summary of your article highlighting

- the medical issue you are addressing,

- the results obtained and

- their clinical impact.

This may be edited to ensure that readers understand the significance and context of the research. Please refer to any of our published articles for an example.

12) For more information: There is space at the end of each article to list relevant web links for further consultation by our readers. Could you identify some relevant ones and provide such information as well? Some examples are patient associations, relevant databases, OMIM/proteins/genes links, author's websites, etc...

13) Author contributions: You will be asked to provide CRediT (Contributor Role Taxonomy) terms in the submission system.

These replace a narrative author contribution section in the manuscript.

14) A Conflict of Interest statement should be provided in the main text.

15) Every published paper now includes a 'Synopsis' to further enhance discoverability. Synopses are displayed on the journal webpage and are freely accessible to all readers. They include a short stand first (maximum of 300 characters, including space) as well as 2-5 one-sentences bullet points that summarizes the paper. Please write the bullet points to summarize the key NEW findings. They should be designed to be complementary to the abstract - i.e. not repeat the same text. We encourage inclusion of key acronyms and quantitative information (maximum of 30 words / bullet point). Please use the passive voice. Please attach these in a separate file or send them by email, we will incorporate them accordingly.

Please also suggest a striking image or visual abstract to illustrate your article as a PNG file 550 px wide x 300-600 px high.

EMBO Molecular Medicine has a "scooping protection" policy, whereby similar findings that are published by others during review or revision are not a criterion for rejection. Should you decide to submit a revised version, I do ask that you get in touch after three months if you have not completed it, to update us on the status.

Please note: When submitting your revision you will be prompted to enter your funding and payment information. This will allow Wiley to send you a quote for the article processing charge (APC) in case of acceptance. This quote takes into account any reduction or fee waivers that you may be eligible for. Authors do not need to pay any fees before their manuscript is accepted and transferred to the publisher.

EMBO Press participates in many Publish and Read agreements that allow authors to publish Open Access with reduced/no publication charges. Check your eligibility: https://authorservices.wiley.com/author-resources/Journal-Authors/open-access/affiliation-policies-payments/index.html

***** Reviewer's comments *****

Referee #1 (Comments on Novelty/Model System for Author):

Novelty - This model system was previously published by this group's 2020 Development paper, but without a focus on it as a disease model for SCTS. Recent reports on MYH3 loss of function in this and other syndromes motivated this study and gave the MYH3 null animal a disease application. The results do suggest an exciting new medical approach to treating these rare MYH3 syndromes, but the model system first needs to better reflect the genetics of the disease as it occurs. As the authors point out MYH3 seems to be a difficult target for generating transgenic animals, so the system they have created is a reasonable starting point. Future study is necessary with the mutations to MYH3 observed in humans in addition to this engineered null condition.

Referee #1 (Remarks for Author):

This study from Bharadwaj, Sharma, and colleagues presents new findings regarding the role of the embryonic myosin heavy chain isoform (MYH3) in muscle development and disease. They use a null MYH3 mouse as a model of SCTS and show 1) YAP signaling is perturbed in the absence of MYH3 and 2) signaling can be normalized by treatment with a small molecule inhibitor of YAP. This is the first application of this transgenic model as a potential model of disease, but the study is incremental There are several major and minor concerns/comments, including:

Major

1) Lines 145 - 148 ("Although Myh3 germline knockout mice (Myh3Δ/Δ) exhibited severe muscle defects affecting myofiber number, area, fiber type and satellite cell numbers in embryonic and early postnatal stages, this did not result in lethality (Agarwal et al., 2020)") seem at odds with a statement in their previous paper: "Myh3Δ/Δ homozygous pups were not obtained at the expected 25% frequency but at about 14% (17 out of 126), suggesting that approximately half of Myh3Δ/Δ genotype animals die in utero during embryonic or fetal stages". The authors need to explain these apparently conflicting statements?

2) The timing of the C2C12 MYH3 knockdown experiment should be clarified (Line 296), and greater detailed methods provided to improve its interpretation. Based on the methods, the cells were transfected with siRNA for 24 hours, then transfected with the luciferase construct for 24 hours, allowed to grow for 24 hours, and finally cultured in low serum differentiation media for 24 hours before assay. After 24 hours in differentiation media did either the control or treated cells have assembled sarcomeric structures? If not, do the authors have any insight into how MYH3 contributes to mechano-transduction in the cell prior to assembly? Are the traction forces different in myoblasts with knockdown? Is the same effect observed if the cells are treated with inhibitors of contraction/cell tension?

3) The grip strength data presented in Figures 1 and 6 seems inconsistent for the same genotype. The 12 - 14 week old / in

Figure 1G range from 5 - 8 gf/gms, but the vehicle control 12 - 14 week old / in Figure 6A appear stronger, ~8 - 9.5 gf/gms. The authors should comment on this? The grip strength data for the +/+ control and treated mice used for Fig EV4B, C should also be presented as it seems like a natural comparator given the phenotypic rescue claims in this section. Figures EV4B and EV4C are not currently referenced in-text.

4) In Figure 1, was the muscle weight as a fraction of body weight also different between genotypes?

5) The authors should provide quantitative data on any differences in activity level observed between null and normal animals. Could activity differences explain some of the observed isoform shifts?

6) Do the myosin isoform shifts observed in the TA and GAS have functional consequences? Are there differences in specific force or contractile kinetics?

Minor

1) Fig 3. KLNO - Labels for genotype would be helpful.

2) Fig 4D - What is the data being presented in the heatmap? emPAI? A label should be included on the scale.

3) Fig 5 - Please label panels I and N to more clearly indicating measures are for mRNA.

Referee #2 (Remarks for Author):

This manuscript describes the adult phenotype of Myh3 knockout mice, along with identification of YAP as a crucial signaling molecule that, when inhibited, can partially rescue the knockout phenotype. The description of the adult mouse skeletal muscle is comprehensive, in terms of its characterization, including change in muscle fiber predominance over time. Remarkably, the molecular defects that were detected in RNA-seq analysis of skeletal muscle were then used to treat P15 mice with a YAP small molecule inhibitor and partially reversed the phenotype. The experimental approach and execution are all high quality with appropriate controls for the immunohistochemistry and Western blots. Overall, this is an exciting manuscript that has translational implications because it identifies a molecular pathway that could potentially be harnessed for therapy in human disease.

Major concerns:
(1) The scoliosis phenotype is incompletely described. Were spinal fusions seen? Were there fusions of other skeletal elements as seen in patients with ? Did the authors use proper technique to measure the Cobb angle (as described in Chen et al., BMC Musculoskeletal Disord, Proper positioning of mice for Cobb angle radiographic measurements, 2021), as these can be misleading if performed without proper technique.
(2) Additional discussion should address the time sensitivity of treatment with the YAP inhibitor. This was initiated at P15, but how does this timing correspond with reductions in Myh3 expression? Would initiation of treatment at later stages be expected to rescue the phenotype?

Minor points:
(1) The y-axis for Figure 2 W and X could be similar for consistency.
(2) Figure 4D shows effects of knockout on other myosins. What is the authors interpretation of these results? This should be added to the discussion.

Referee #3 (Comments on Novelty/Model System for Author):

This is a fairly novel manuscript. The analysis of the Myh3 KO defect in combination with a pharmacological manipulation of the Yap inhibitor (CA3) is highly novel for a mechanism of mechanotransduction. There just needs to be some clarification as highlighted in my review.

Referee #3 (Remarks for Author):

The manuscript by Bharadwaj and colleagues is centered on the musculoskeletal defects associated with Myh3-deficiency in

skeletal muscle. Recessive loss-of-function mutations in MYH3 cause spondylocarpotarsal synostosis (SCTS), characterized by vertebral fusions and scoliosis, which is also observed in the Myh3 knockout (KO) mouse model. The authors further characterize these and demonstrate them to exhibit changes in muscle fiber type, altered satellite cell numbers and increased skeletal muscle fibrosis. Their mass spectrometric analysis of embryonic skeletal muscle from Myh3 KO muscles revealed integrin signaling and cytoskeletal regulation as the most affected pathways. YAP signaling is predominantly a regulator of these pathways. The authors evaluated the YAP inhibitor CA3 and showed that it rescued many of the satellite cell and muscle pathophysiological defects in the Myh3 KO mice. The authors conclude that YAP-mediated defects are driving Myh3 pathologies and that modulation of this pathway may be a strategy for therapeutic intervention.

Overall, this is a well-written manuscript with logically-designed experiments. The findings are of interest to the ultra-rare disease community that might be affected with SCTC but could be adapted potentially to other similar mechanotransduction disorders affected by disrupted Yap signaling. However, there are a few questions that warrant probing into the proposed mechanism by the authors. Some examination of the Yap-Taz dynamics and TEAD activation is warranted to validate the proposed mechanism of dysregulated in the Myh3 knockout mice.

Major Comments:

1. It would be helpful to know if the decreased Pax7-positive satellite cells observed in the Myh3 KO mice are due to hyper-activation of the satellite cells resulting in their fusion and/or apoptosis? Can the authors comment or have the authors evaluated the differentiation capacity of the satellite cells from the Myh3 KO mice? If they are in the activated state early on in development (Fig. 3G) but decrease later (Fig. 3I), they are likely exhausted and would be either fusing/differentiating or undergoing apoptosis. Markers for both can be evaluated.

2. Figure 7B/Model proposes that TEAD-mediated Yap target gene activation does not occur because of the loss of Myh3. The authors assume this based on Yap target gene activation; however there are some Yap and Taz independent activation of TEAD myogenic factors (Sun et al., Stem Cells, 2017). The authors should determine if TEAD actually does change in its localization and/or expression in the Myh3 KO muscle. This would go a long way in strengthening their proposed model.

3. Minor comment. I would fix the labeling of the protein immunoblots in Figure 4E, some extra spaces between the numbers.

4. Can the authors comment on whether the other myosins (MYH8 appears to be upregulated; Fig. 4D) similarly have any impact on Yap/Hippo signaling?

5. Does TAZ/WWTR1 change in expression and/or localization in the Myh3 KO mouse muscles? It is not clear to me if the changes observed in Hippo signaling are TAZ dependent or independent?

6. Yap has been proposed to signal through dystrophin/dystrophin-associated protein complex (DAPC) (Iyer et al., AJP-Cell Physiol., 2019). Did dystrophin or other DAPC proteins come up in the authors' mass-spectrometry analyses?

7. Minor comment. Based on the authors' figure legends, were only male mice used? Please clarify in the methods section.

8. Similar to my first question. Supp. EV3. Are the decreased satellite cells due to increased fusion or increased apoptosis of the satellite cells themselves?

Response to Reviewer Comments

We are grateful to the Reviewers for evaluating our manuscript and providing excellent comments and suggestions. We have attempted to address every comment to the best of our ability, resulting in several changes to the revised version of the manuscript that we are submitting. Briefly, new data has been added to Figure 1, Figure 3, Figure 4, Figure EV1, Figure EV4 and Figure EV5. Old Figure EV4 data has been moved to Figure EV5 where new data has been incorporated; revised Figure EV4 has fully new data. Please find below our detailed response to the Reviewer comments inline. The line numbers in the response below refer to the highlighted version of the manuscript; we find that the line numbers are changing during word to PDF conversion on the submission portal and therefore request the Reviewers to use the line numbers with respect to the revised, highlighted manuscript which has been submitted as a Supplementary PDF. In this same manuscript, the changes that have been made to the text during revision are highlighted in yellow and in track change mode to show the parts that have been moved or deleted. We hope that the revised manuscript addresses all the queries raised, improving it substantially.

Referee #1 (Comments on Novelty/Model System for Author):

Novelty - This model system was previously published by this group's 2020 Development paper, but without a focus on it as a disease model for SCTS. Recent reports on MYH3 loss of function in this and other syndromes motivated this study and gave the MYH3 null animal a disease application. The results do suggest an exciting new medical approach to treating these rare MYH3 syndromes, but the model system first needs to better reflect the genetics of the disease as it occurs. As the authors point out MYH3 seems to be a difficult target for generating transgenic animals, so the system they have created is a reasonable starting point. Future study is necessary with the mutations to MYH3 observed in humans in addition to this engineered null condition.
We thank the Reviewer for the comments and agree that this study could be the starting point for future studies on human disorders characterized by *MYH3* mutations. As pointed out by the Reviewer, our results identify YAP signalling as a novel potential target in such disorders, which could be valuable in future patient-directed approaches.

Referee #1 (Remarks for Author):

This study from Bharadwaj, Sharma, and colleagues presents new findings regarding the role of the embryonic myosin heavy chain isoform (MYH3) in muscle development and disease. They use a null MYH3 mouse as a model of SCTS and show 1) YAP signaling is perturbed in the absence of MYH3 and 2) signaling can be normalized by treatment with a small molecule inhibitor of YAP. This is the first application of this transgenic model as a potential model of disease, but the study is incremental. There are several major and minor concerns/comments, including:

Major

1) Lines 145 - 148 ("Although Myh3 germline knockout mice (Myh3Δ/Δ) exhibited severe muscle defects affecting myofiber number, area, fiber type and satellite cell numbers in embryonic and early postnatal stages, this did not result in lethality (Agarwal et al., 2020)") seem at odds with a statement in their previous paper: "Myh3Δ/Δ homozygous pups were not

obtained at the expected 25% frequency but at about 14% (17 out of 126), suggesting that approximately half of Myh3Δ/Δ genotype animals die in utero during embryonic or fetal stages". The authors need to explain these apparently conflicting statements?

We thank the Reviewer for pointing this out. What we meant to convey was that germline knockout of *Myh3* did not result in complete lethality; as explained in the earlier publication (Agarwal et al., 2020), in a cross between *Myh3*$^{Δ/+}$ male to *Myh3*$^{Δ/+}$ female, of the expected 25% *Myh3*$^{Δ/Δ}$ pups, only 14% were born. The remaining 11% die in utero during embryonic or fetal stages of development. The surviving 14% of *Myh3*$^{Δ/Δ}$ pups that are born were used in this study. Based on the Reviewer suggestion, we have added additional sentences and modified the section as follows: "As previously described, we observed 11% lethality of *Myh3* germline knockout (*Myh3*$^{Δ/Δ}$) embryos during developmental stages; the remaining 14% completed embryonic development and were born as pups (Agarwal et al., 2020). Although these surviving *Myh3* germline knockout mice ~~(*Myh3*$^{Δ/Δ}$)~~ exhibited severe muscle defects affecting myofiber number, area, fiber type and satellite cell numbers in embryonic and early postnatal stages, this did not result in increased postnatal lethality compared to controls (Agarwal et al., 2020)" lines 143-148.

2) The timing of the C2C12 MYH3 knockdown experiment should be clarified (Line 296), and greater detailed methods provided to improve its interpretation. Based on the methods, the cells were transfected with siRNA for 24 hours, then transfected with the luciferase construct for 24 hours, allowed to grow for 24 hours, and finally cultured in low serum differentiation media for 24 hours before assay. After 24 hours in differentiation media did either the control or treated cells have assembled sarcomeric structures? If not, do the authors have any insight into how MYH3 contributes to mechano-transduction in the cell prior to assembly?

We have added more details for the C2C12 *Myh3* knockdown experiment as suggested by the Reviewer. The sentence has been modified to "To confirm the increased YAP activity, we carried out a luciferase reporter assay by transfecting the YAP responsive *8xGTIIC-luciferase* plasmid into C2C12 mouse myogenic cells treated with a control siRNA or *Myh3* siRNA and allowing the cells to differentiate for 24 hours (Dupont *et al.*, 2011)" (lines 315-318). The methodology used for the experiment is as described by the Reviewer (cells transfected with siRNA for 24 hours, then transfected with the *8xGTIIC-luciferase* plasmid for 24 hours, allowed to grow for 24 hours, followed by change to differentiation media and culturing for 24 hours). The luciferase assay is carried out 96 hours after siRNA transfection. The reason to do the experiment in this manner is twofold: first, MyHC-embryonic is the earliest MyHC to be expressed and might have roles in early stages of sarcomere formation, which can be captured by observing early stages of differentiation; second, the effect of the siRNA knockdown is transient and looking at early time points of differentiation ensures that the knockdown persists during the assay. To address the Reviewer comment, we have done MyHC-embryonic blots on the samples, which clearly shows expression in control siRNA treated samples and knockdown in *Myh3* siRNA treated samples (Figure 4H-I). Total and phospho-YAP expression is seen in C2C12 cells at this time point (Figure 4H-I). Differentiating myofibers can be clearly observed in these cells and alpha-actinin, a Z-disc protein in the sarcomere, is expressed in C2C12 cells treated in this manner (see image below showing alpha-actinin labelling in red, where arrows point to alpha-actinin puncta marking sarcomere formation). Since MyHC-embryonic and alpha-actinin expression are seen, sarcomere formation has initiated in C2C12 cells where the luciferase assay has been carried out. MyHC-embryonic probably contributes to mechano-transduction during sarcomere assembly, since most studies indicate that MyHC-embryonic expression occurs in differentiating muscle cells, where sarcomere assembly is underway. However, whether MyHC-embryonic has non-sarcomeric functions is unclear, especially since

its expression has been reported in non-skeletal muscle cell types in some studies (Rice and Leinwand, J Cell Biol 2003; Zieba et al, Scientific Reports 2017).

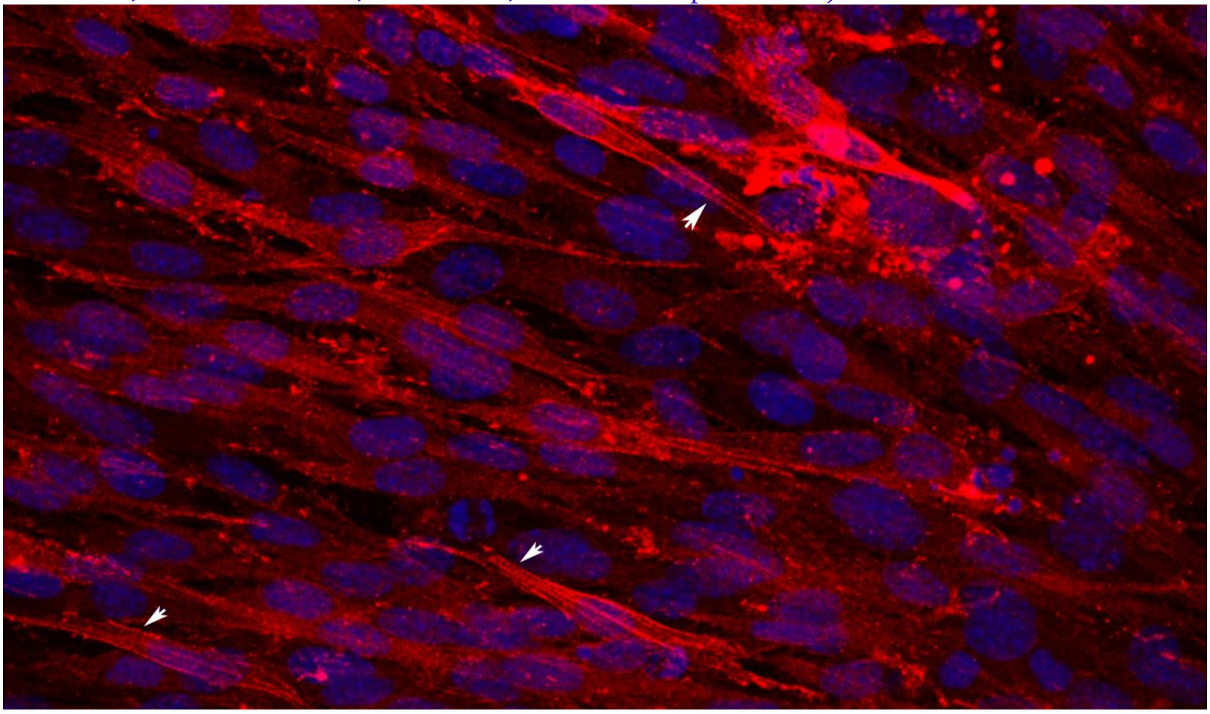

Are the traction forces different in myoblasts with knockdown? Is the same effect observed if the cells are treated with inhibitors of contraction/cell tension.

These are very interesting points raised by the Reviewer. For traction force, we carried out a contractility measurement assay by growing control and *Myh3* siRNA treated differentiating C2C12 cells on a specialized matrix which shrinks based on the contractile forces exerted by the cells, which was quantified and found to be reduced in *Myh3* depleted cells (Figure EV4E-F and lines 365-368). ATPase activity of myosin protein isolated from E16.5 control and *Myh3* knockout mouse embryos clearly suggest reduced ATPase activity in *Myh3* knockout embryos, indicating the requirement of MyHC-embryonic (Figure EV1M and Lines 182-183).

Based on the Reviewer comment, we treated C2C12 cells with para-aminoblebbistatin, a myosin inhibitor, which led to increased levels of total YAP and reduced levels of phospho-YAP (Figure EV4A-B, lines 359-361 and 537-539). This is similar to the effect we observed in *Myh3* knockouts (Figure 4H-I and Lines 321-323). Interestingly, another contractility inhibitor, 2,3-Butanedione monoxime (BDM) had a somewhat different effect, where its treatment caused no change in total YAP levels but increase in phospho-YAP levels (Figure EV4C-D and Lines 361-363). The differences observed could be related to the specific effects of these inhibitors on different myosin isoforms and the particular activity they target. Overall, these experiments clearly indicate that inhibitors of contraction do have effects on YAP signalling in skeletal muscle cells, confirming the effects mediated by MyHC-embryonic on YAP that we observe.

3) The grip strength data presented in Figures 1 and 6 seems inconsistent for the same genotype. The 12 - 14 week old Δ/Δ in Figure 1G range from 5 - 8 gf/gms, but the vehicle control 12 - 14 week old Δ/Δ in Figure 6A appear stronger, ~8 - 9.5 gf/gms. The authors should comment on this? The grip strength data for the +/+ control and treated mice used for Fig EV4B, C should also be presented as it seems like a natural comparator given the phenotypic rescue claims in this section. Figures EV4B and EV4C are not currently referenced in-text.

There is some amount of variability observed in the grip strength, especially at the 12-week time point (Figure 1R, Figure 6A). We agree that there is a slight increase in grip strength in the *Myh3*$^{\Delta/\Delta}$ vehicle controls in Figure 6A, compared to Figure 1R. The only difference between these mice is the treatment with DMSO as the vehicle control in Figure 6. Several studies report that DMSO might have effects on skeletal muscle contractility, excitation-contraction coupling and differentiation. We have not explored this further, since our direct comparisons are between the vehicle treated mice and the CA3 treated mice (where CA3 is reconstituted in DMSO). We have now also included new data on vehicle and CA3 treatment starting in adult stage (6 weeks) instead of perinatal stage, where we observe a similar, slightly increased grip strength (Figure EV5F). We have added a sentence to address this comment "A slight increase in grip strength is observed in the vehicle treated *Myh3*$^{\Delta/\Delta}$ mice (Figure 6A and EV5F) compared to untreated ones (Figure 1R), which could suggest an effect of the vehicle DMSO on muscle function" (lines 387-389). As suggested by the Reviewer, we have included the *Myh3*$^{+/+}$ data for treated mice used (Figure EV5D). We thank the Reviewer for pointing out some panels that were not referenced in the text previously, which have now been referenced (lines 409-411).

4) In Figure 1, was the muscle weight as a fraction of body weight also different between genotypes?
The muscle weight as a fraction of body weight was significantly different for the tibialis anterior (TA) and the quadriceps, but was not significantly different for the gastrocnemius. Thus, loss of MyHC-embryonic might affect specific muscles in diverse ways, possibly as a consequence of the fiber type composition of the specific muscle. The gastrocnemius has more MyHC-slow+ fibers compared to the TA and quadriceps, and loss of MyHC-embryonic reduces MyHC-slow levels drastically (Figure 2M-P, V-X and lines 195-198, 201-202, 204-206). However, the total number of myofibers are unchanged in the gastrocnemius of *Myh3*$^{\Delta/\Delta}$ mice (Figure EV1A, E). This indicates that replacement of the MyHC-slow+ fibers by MyHC-IIa+ and IIb+ fibers is able to compensate for weight in the case of the gastrocnemius (Figure EV2C-G).

5) The authors should provide quantitative data on any differences in activity level observed between null and normal animals. Could activity differences explain some of the observed isoform shifts?
We thank the Reviewer for this suggestion. To address this, we have carried out rotarod and treadmill assays comparing activity levels between control and *Myh3*$^{\Delta/\Delta}$ mice. This data has been included in Figure 1S-T and Figure EV1J, referred to in lines 179-183 in the revised manuscript. We observe a significant reduction in activity in the *Myh3*$^{\Delta/\Delta}$ mice using both techniques. It is likely that the decreased activity underlies some of the MyHC isoform shifts that are observed in the adult *Myh3*$^{\Delta/\Delta}$ mice. However, we cannot rule out the possibility that the isoform shifts lead to the decreased activity. This raises the interesting conundrum as to whether the decreased activity precedes the isoform shift or vice versa, which is a difficult question to address.

6) Do the myosin isoform shifts observed in the TA and GAS have functional consequences? Are there differences in specific force or contractile kinetics?
Yes, there are functional consequences as seen with the effect on activity levels on rotarod and treadmill assays as described for the previous comment. We observe a reduction in the myosin ATPase activity in the E16.5 *Myh3*$^{\Delta/\Delta}$ embryos compared to controls (Figure EV1M and Lines 182-183). We also observe changes in contractility in C2C12 cells where *Myh3* has been depleted using siRNA (Figure EV4E-F and Lines 365-368).

Minor

1) Fig 3. KLNO - Labels for genotype would be helpful.
Genotype labels have been added to these panels as suggested.

2) Fig 4D - What is the data being presented in the heatmap? emPAI? A label should be included on the scale.
Yes, the data presented in the heatmap is emPAI, which has now been mentioned on the scale.

3) Fig 5 - Please label panels I and N to more clearly indicating measures are for mRNA
We have now made the Y-axis "mRNA Relative fold change" for these panels to indicate that transcript levels are quantified in these graphs.

Referee #2 (Remarks for Author):

This manuscript describes the adult phenotype of Myh3 knockout mice, along with identification of YAP as a crucial signaling molecule that, when inhibited, can partially rescue the knockout phenotype. The description of the adult mouse skeletal muscle is comprehensive, in terms of its characterization, including change in muscle fiber predominance over time. Remarkably, the molecular defects that were detected in RNA-seq analysis of skeletal muscle were then used to treat P15 mice with a YAP small molecule inhibitor and partially reversed the phenotype. The experimental approach and execution are all high quality with appropriate controls for the immunohistochemistry and Western blots. Overall, this is an exciting manuscript that has translational implications because it identifies a molecular pathway that could potentially be harnessed for therapy in human disease.
We thank the Reviewer for the comments and agree that the YAP pathway could be of great interest as a molecular target to treat *MYH3*-associated human musculoskeletal disorders.

Major concerns:

(1) The scoliosis phenotype is incompletely described. Were spinal fusions seen? Were there fusions of other skeletal elements as seen in patients with ? Did the authors use proper technique to measure the Cobb angle (as described in Chen et al., BMC Musculoskeletal Disord, Proper positioning of mice for Cobb angle radiographic measurements, 2021), as these can be misleading if performed without proper technique.
We thank the Reviewer for this suggestion and looked more closely for other phenotypes characteristic of *MYH3* mutations. We observe severe spinal fusions in the *Myh3*$^{\Delta/\Delta}$ mice, representative images and quantification of which have now been incorporated in the revised version of the manuscript (Figure 1N-Q; Figure EV1K, L,N,O). We have ensured that the mice are oriented properly for the Cobb angle measurements and followed the technique suggested by Chen et al and have now cited this paper in the materials and methods.

(2) Additional discussion should address the time sensitivity of treatment with the YAP inhibitor. This was initiated at P15, but how does this timing correspond with reductions in Myh3 expression? Would initiation of treatment at later stages be expected to rescue the phenotype?
Based on this suggestion by the Reviewer, we have carried out new experiments where YAP inhibitor treatment was initiated later in postnatal life in *Myh3* knockouts, starting at 6 weeks after birth. This fails to rescue most of the phenotypes exhibited by *Myh3* knockouts, such as

reduced grip strength, increased number of myofibers per cross sectional area, reduction in myofiber size, elevated fibrosis, altered MyHC-IIb, -IIa and -slow levels and severity of scoliosis. The only phenotypes which exhibited a rescue were decrease in Pax7 levels in the TA and reduction in expression of the YAP target gene CTGF in the TA. Therefore, we conclude that inhibiting YAP late during postnatal stages does not lead to a proper rescue of the abnormalities exhibited by *Myh3*$^{\Delta/\Delta}$ mice. All of this data has been incorporated in Figure EV5E-R and is referred to in lines 415-428 and 432-436.

Minor points:

(1) The y-axis for Figure 2 W and X could be similar for consistency.
We have made this change as suggested by the Reviewer (Figure 2W, X).

(2) Figure 4D shows effects of knockout on other myosins. What is the authors interpretation of these results? This should be added to the discussion.
We have added a paragraph to the discussion in the revised manuscript as suggested by the Reviewer, detailing the effect of *Myh3* knockout on other myosins (lines 517-539).

Referee #3 (Comments on Novelty/Model System for Author):

This is a fairly novel manuscript. The analysis of the Myh3 KO defect in combination with a pharmacological manipulation of the Yap inhibitor (CA3) is highly novel for a mechanism of mechanotransduction. There just needs to be some clarification as highlighted in my review.
We are thankful to the Reviewer for the comments and agree that YAP signalling opens up a novel molecular target for *MYH3*-associated musculoskeletal disorders, which needs to be explored further.

Referee #3 (Remarks for Author):

The manuscript by Bharadwaj and colleagues is centered on the musculoskeletal defects associated with Myh3-deficiency in skeletal muscle. Recessive loss-of-function mutations in MYH3 cause spondylocarpotarsal synostosis (SCTS), characterized by vertebral fusions and scoliosis, which is also observed in the Myh3 knockout (KO) mouse model. The authors further characterize these and demonstrate them to exhibit changes in muscle fiber type, altered satellite cell numbers and increased skeletal muscle fibrosis. Their mass spectrometric analysis of embryonic skeletal muscle from Myh3 KO muscles revealed integrin signaling and cytoskeletal regulation as the most affected pathways. YAP signaling is predominantly a regulator of these pathways. The authors evaluated the YAP inhibitor CA3 and showed that it rescued many of the satellite cell and muscle pathophysiological defects in the Myh3 KO mice. The authors conclude that YAP-mediated defects are driving Myh3 pathologies and that modulation of this pathway may be a strategy for therapeutic intervention.

Overall, this is a well-written manuscript with logically-designed experiments. The findings are of interest to the ultra-rare disease community that might be affected with SCTC but could be adapted potentially to other similar mechanotransduction disorders affected by disrupted Yap signaling. However, there are a few questions that warrant probing into the proposed mechanism by the authors. Some examination of the Yap-Taz dynamics and TEAD activation is warranted to validate the proposed mechanism of dysregulated in the Myh3 knockout mice.

Major Comments:

1. It would be helpful to know if the decreased Pax7-positive satellite cells observed in the Myh3 KO mice are due to hyper-activation of the satellite cells resulting in their fusion and/or apoptosis?
Can the authors comment or have the authors evaluated the differentiation capacity of the satellite cells from the Myh3 KO mice?
If they are in the activated state early on in development (Fig. 3G) but decrease later (Fig. 3I), they are likely exhausted and would be either fusing/differentiating or undergoing apoptosis. Markers for both can be evaluated.
These are all excellent questions and suggestions by the Reviewer and we have tried to address them as follows.
Satellite cells in *Myh3* KO mice indeed seem to be hyper-activated. We performed single muscle fiber isolation (along with satellite cells resident on the fibers) and culture experiments from 8-10-week old control and *Myh3* KO mice. We find that the satellite cells of *Myh3* KO mice exhibit increased activation as shown by greater proportion of satellite cells having MyoD expression. This data has been added to the manuscript (Figure 3N-P) and described (lines 252-257 and 510-513).
Regarding the differentiation capability of satellite cells from *Myh3* KO mice, we have already shown this data in our previous paper characterizing the embryonic and perinatal role of Myh3 (Figure 5K and Figure S5C-D in Agarwal et al, Development, 2020). We do observe a significant reduction in fusion index in satellite cells from *Myh3* KO mice, most likely due to exhaustion of the satellite cell pool.
To address the third part of this comment, we carried out western blots for MyoD (differentiation marker) and Caspase3 (apoptosis marker). We observe elevated MyoD levels at the early time point (8-10 weeks) and reduced MyoD levels at the later time point (6 months), indicating exhaustion of the satellite cell pool with age (Figure 3G, I, L and lines 242-252). With respect to apoptosis, Caspase3 levels are significantly reduced at 8-10 weeks and trends towards an increase which is not significant at 6 months (Figure 3G, J, M and lines 242-252).

2. Figure 7B/Model proposes that TEAD-mediated Yap target gene activation does not occur because of the loss of Myh3. The authors assume this based on Yap target gene activation; however there are some Yap and Taz independent activation of TEAD myogenic factors (Sun et al., Stem Cells, 2017). The authors should determine if TEAD actually does change in its localization and/or expression in the Myh3 KO muscle. This would go a long way in strengthening their proposed model
We thank the Reviewer and agree that YAP-TAZ independent TEAD activation does occur in many instances as pointed out. To clarify this, we looked at TEAD localization in the cytoplasmic and nuclear fractions in skeletal muscle lysates from 4-month old control and *Myh3*$^{\Delta/\Delta}$ mice. We were unable to detect TEAD in the cytoplasmic fraction but observed similar TEAD levels in the nuclear and total muscle lysates, indicating that TEAD localization or levels do not undergo significant changes in *Myh3* KO mice (Figure EV4G-L and lines 370-374).

3. Minor comment. I would fix the labeling of the protein immunoblots in Figure 4E, some extra spaces between the numbers.
We thank the Reviewer for pointing this out and have made corrections to these labels.

4. Can the authors comment on whether the other myosins (MYH8 appears to be upregulated; Fig. 4D) similarly have any impact on Yap/Hippo signaling?

Based on the Reviewer comment, we performed knockdown of *Myh8* which codes for MyHC-perinatal, another skeletal muscle myosin heavy chain expressed during development and regeneration, in C2C12 cells. Interestingly, total YAP levels are increased and phospho-YAP (S109) levels are decreased upon *Myh8* knockdown, indicating that the effect on YAP signalling might be a conserved phenomenon exhibited by developmental MyHCs (Figure 4J-K; lines 323-327 and 525-527).

5. Does TAZ/WWTR1 change in expression and/or localization in the Myh3 KO mouse muscles? It is not clear to me if the changes observed in Hippo signaling are TAZ dependent or independent?
Similar to TEAD, TAZ levels do not change in *Myh3* KO mice, although unlike TEAD, we do observe cytoplasmic TAZ in addition to nuclear TAZ (Figure EV4E-J and lines 369-374).

6. Yap has been proposed to signal through dystrophin/dystrophin-associated protein complex (DAPC) (Iyer et al., AJP-Cell Physiol., 2019). Did dystrophin or other DAPC proteins come up in the authors' mass-spectrometry analyses?
Dystrophin or DAPC proteins did not come up in our mass spectrometry analysis but Filamin B was the top downregulated protein in *Myh3* KO. Filamin B does interact with Dystrophin and mutations in Filamin B lead to spondylocarpotarsal synostosis (SCTS), the same congenital disorder. We have confirmed the Filamin B downregulation by western blots (Figure 4E-F) and added this to the text (lines 299-310 and 457-459).

7. Minor comment. Based on the authors' figure legends, were only male mice used? Please clarify in the methods section.
We are grateful for this suggestion. Male and female segregation was done for body weight, muscle weight, grip strength, rotarod and treadmill assays, where there are known differences between sexes. For other parameters, such segregation was not done. We have now stated this in the methods (lines 604-606).

8. Similar to my first question. Supp. EV3. Are the decreased satellite cells due to increased fusion or increased apoptosis of the satellite cells themselves?
Based on the single fiber experiments, we believe that the decreased satellite cell numbers are due to increased satellite cell activation and fusion (Figure 3N-P; lines 252-257 and 510-513).

2nd Jul 2023

Dear Dr. Mathew,

Thank you for the submission of your revised manuscript to EMBO Molecular Medicine. I am pleased to inform you that we will be able to accept your manuscript pending the following final amendments:

1) Please address the referee #2 minor suggestion by discussing the relationship between MYH3 and FLNB.
2) In the main manuscript file, please do the following:
- Correct/answer the track changes suggested by our data editors by working from the attached document.
- Reduce number of keywords to max. 5.
- Please add callouts for supplementary (Appendix) tables at appropriate places in the text. Further, all figures and panels should be called out in a sequential order. Currently Fig. 3I is called out after Fig. 3K, Fig. 6J after Fig. 6K and Fig. 6M after Fig 6N. Please correct. Also, please call out all individual panels of Figure EV1 and EV5 sequentially.
- In M&M, the statistical paragraph should reflect all information that you have filled in the Authors Checklist, especially regarding randomization, blinding, replication.
- Please rename "Conflict of Interest" to "Disclosure Statement & Competing Interests". We updated our journal's competing interests policy in January 2022 and request authors to consider both actual and perceived competing interests. Please review the policy https://www.embopress.org/competing-interests and update your competing interests if necessary.
- Author contributions: Please remove it from the manuscript and specify author contributions in our submission system. CRediT has replaced the traditional author contributions section because it offers a systematic machine-readable author contributions format that allows for more effective research assessment. You are encouraged to use the free text boxes beneath each contributing author's name to add specific details on the author's contribution. More information is available in our guide to authors:
https://www.embopress.org/page/journal/17574684/authorguide#authorshipguidelines
3) Appendix: Please rename "Supporting information" to "Appendix" and remove all EV Figures and their legends and leave only the 2 tables. Tables should be renamed to Appendix Table S1 and S2 and the table of content amended accordingly. EV Figure legends should be moved to the main manuscript file and placed after the main figure legends.
4) Funding: Please make sure the information about funding are complete in both our submission systema and in the manuscript. Currently information about the Regional Centre for Biotechnology (RCB), Indian Council of Medical Research (ICMR) , the University Grants Commission (UGC), Department of Science and Technology INSPIRE junior research fellowship, ICMR junior research fellowship and the Science and Engineering Research Board are missing in our submission system.
5) The Paper Explained: Please provide "The Paper Explained" and add it to the main manuscript text. Please refer to any of our published primary research articles for an example. Check "Author Guidelines" for more information.
https://www.embopress.org/page/journal/17574684/authorguide#researcharticleguide
6) Synopsis: Every published paper now includes a 'Synopsis' to further enhance discoverability. Synopses are displayed on the journal webpage and are freely accessible to all readers. They include separate synopsis image and synopsis text.
- Synopsis image: Please provide a striking image or visual abstract as a high-resolution jpeg file 550 px-wide x (250-400)-px high to illustrate your article.
- Synopsis text: Please provide a short standfirst (maximum of 300 characters, including space) as well as 2-5 one sentence bullet points that summarise the paper as a .doc file. Please write the bullet points to summarise the key NEW findings. They should be designed to be complementary to the abstract - i.e. not repeat the same text. We encourage inclusion of key acronyms and quantitative information (maximum of 30 words / bullet point). Please use the passive voice.
- Please check your synopsis text and image before submission with your revised manuscript. Please be aware that in the proof stage minor corrections only are allowed (e.g., typos).
7) For more information: This space should be used to list relevant web links for further consultation by our readers. Could you identify some relevant ones and provide such information as well? Some examples are patient associations, relevant databases, OMIM/proteins/genes links, author's websites, etc...
8) As part of the EMBO Publications transparent editorial process initiative (see our Editorial at http://embomolmed.embopress.org/content/2/9/329), EMBO Molecular Medicine will publish online a Review Process File (RPF) to accompany accepted manuscripts. This file will be published in conjunction with your paper and will include the anonymous referee reports, your point-by-point response and all pertinent correspondence relating to the manuscript. Let us know whether you agree with the publication of the RPF and as here, if you want to remove or not any figures from it prior to publication. Please note that the Authors checklist will be published at the end of the RPF.
9) Please provide a point-by-point letter INCLUDING my comments as well as the reviewer's reports and your detailed responses (as Word file).

I look forward to reading a new revised version of your manuscript as soon as possible.

Yours sincerely,

Zeljko Durdevic

Zeljko Durdevic
Editor
EMBO Molecular Medicine

*** Instructions to submit your revised manuscript ***

*** PLEASE NOTE *** As part of the EMBO Publications transparent editorial process initiative (see our Editorial at https://www.embopress.org/doi/pdf/10.1002/emmm.201000094), EMBO Molecular Medicine will publish online a Review Process File to accompany accepted manuscripts.

In the event of acceptance, this file will be published in conjunction with your paper and will include the anonymous referee reports, your point-by-point response and all pertinent correspondence relating to the manuscript. If you do NOT want this file to be published, please inform the editorial office at contact@embomolmed.org.

When submitting your revised manuscript, please include:

1) a .docx formatted version of the manuscript text (including Figure legends and tables)

2) Separate figure files*

3) supplemental information as Expanded View and/or Appendix. Please carefully check the authors guidelines for formatting Expanded view and Appendix figures and tables at https://www.embopress.org/page/journal/17574684/authorguide#expandedview

4) a letter INCLUDING the reviewer's reports and your detailed responses to their comments (as Word file).

5) The paper explained: EMBO Molecular Medicine articles are accompanied by a summary of the articles to emphasize the major findings in the paper and their medical implications for the non-specialist reader. Please provide a draft summary of your article highlighting
- the medical issue you are addressing,
- the results obtained and
- their clinical impact.
This may be edited to ensure that readers understand the significance and context of the research.
Please refer to any of our published articles for an example.

6) For more information: There is space at the end of each article to list relevant web links for further consultation by our readers. Could you identify some relevant ones and provide such information as well? Some examples are patient associations, relevant databases, OMIM/proteins/genes links, author's websites, etc...

7) Author contributions: the contribution of every author must be detailed in a separate section.

8) EMBO Molecular Medicine now requires a complete author checklist (https://www.embopress.org/page/journal/17574684/authorguide) to be submitted with all revised manuscripts. Please use the checklist as guideline for the sort of information we need WITHIN the manuscript. The checklist should only be filled with page numbers were the information can be found. This is particularly important for animal reporting, antibody dilutions (missing) and exact values and n that should be indicted instead of a range.

9) Every published paper now includes a 'Synopsis' to further enhance discoverability. Synopses are displayed on the journal webpage and are freely accessible to all readers. They include a short stand first (maximum of 300 characters, including space) as well as 2-5 one sentence bullet points that summarise the paper. Please write the bullet points to summarise the key NEW findings. They should be designed to be complementary to the abstract - i.e. not repeat the same text. We encourage inclusion of key acronyms and quantitative information (maximum of 30 words / bullet point). Please use the passive voice. Please attach

these in a separate file or send them by email, we will incorporate them accordingly.

You are also welcome to suggest a striking image or visual abstract to illustrate your article. If you do please provide a jpeg file 550 px-wide x 400-px high.

10) A Conflict of Interest statement should be provided in the main text

11) Please note that we now mandate that all corresponding authors list an ORCID digital identifier. This takes <90 seconds to complete. We encourage all authors to supply an ORCID identifier, which will be linked to their name for unambiguous name identification.

Currently, our records indicate that the ORCID for your account is 0000-0001-7908-6094.

Please click the link below to modify this ORCID:
Link Not Available

12) The system will prompt you to fill in your funding and payment information. This will allow Wiley to send you a quote for the article processing charge (APC) in case of acceptance. This quote takes into account any reduction or fee waivers that you may be eligible for. Authors do not need to pay any fees before their manuscript is accepted and transferred to our publisher.

*Additional important information regarding Figures

Each figure should be given in a separate file and should have the following resolution:
Graphs 800-1,200 DPI
Photos 400-800 DPI
Colour (only CMYK) 300-400 DPI"

Figures are not edited by the production team. All lettering should be the same size and style; figure panels should be indicated by capital letters (A, B, C etc). Gridlines are not allowed except for log plots. Figures should be numbered in the order of their appearance in the text with Arabic numerals. Each Figure must have a separate legend and a caption is needed for each panel.

*Additional important information regarding figures and illustrations can be found at https://bit.ly/EMBOPressFigurePreparationGuideline. See also figure legend preparation guidelines: https://www.embopress.org/page/journal/17574684/authorguide#figureformat

The system will prompt you to fill in your funding and payment information. This will allow Wiley to send you a quote for the article processing charge (APC) in case of acceptance. This quote takes into account any reduction or fee waivers that you may be eligible for. Authors do not need to pay any fees before their manuscript is accepted and transferred to our publisher.

***** Reviewer's comments *****

Referee #2 (Comments on Novelty/Model System for Author):

This is an exciting manuscript linking early contractility to YAP/hippo signaling. The effects of early loss of MYH3 are striking on these pathways and the rescue of the phenotype with YAP inhibitors may lead to novel human treatments.

Referee #2 (Remarks for Author):

The authors have carefully responded to all prior reviews with additional experiments that provide additional support for their conclusions, even that the hippo pathway is also altered by changes in other early expressed myosin genes. My only minor suggestion would be to add 1-3 sentences that emphasize the relationship between MYH3 and FLNB. Knockout of MYH3 caused marked reduction in FLNB and both alterations of both genes cause SCTS, which were noted by the reviewers. Is there existing data about loss of FLNB that showing similar effects on downstream targets?

All prior questions have been adequately addressed in the manuscript and in the response to reviewers.

Referee #3 (Comments on Novelty/Model System for Author):

The manuscript is appropriate to evaluate the roles of Myosin Heavy Chain (specifically Myh3) which has an impact on the human spondylocarpotarsal synostosis (SCTS) disorder. The ability to dissect out individual isoforms and adequately model their function is appropriate in a knockout mouse model. No concerns are noted with the model choice.

Referee #3 (Remarks for Author):

The authors have done an impressive job in response to my and the other reviewer comments. I appreciate the additional work elucidating additional satellite cell expression and acknowledge that some of their previous work focused on satellite cells in the Myh3 knockout. Additional functional testing of the Myh3 knockout mice is supportive of the model and the authors' hypotheses. Additional supportive data on TEAD localization is also well conducted. I have no additional concerns and believe that the manuscript is suitable for publication in EMBO Molecular Medicine.

Response to Editor and Reviewer Comments

Thank you for the submission of your revised manuscript to EMBO Molecular Medicine. I am pleased to inform you that we will be able to accept your manuscript pending the following final amendments:

1) Please address the referee #2 minor suggestion by discussing the relationship between MYH3 and FLNB.
We have now added a sentence in the discussion (line numbers 485-487) and cited two references to address this suggestion.

2) In the main manuscript file, please do the following:
- Correct/answer the track changes suggested by our data editors by working from the attached document.
We have made the changes as suggested.
- Reduce number of keywords to max. 5.
Done.
- Please add callouts for supplementary (Appendix) tables at appropriate places in the text. Further, all figures and panels should be called out in a sequential order. Currently Fig. 3I is called out after Fig. 3K, Fig. 6J after Fig. 6K and Fig. 6M after Fig 6N. Please correct. Also, please call out all individual panels of Figure EV1 and EV5 sequentially.
We have made the changes either by changing the order in which figure panels are called out in the text or by changing the position of panels in the figures.
- In M&M, the statistical paragraph should reflect all information that you have filled in the Authors Checklist, especially regarding randomization, blinding, replication.
We have made changes as suggested.
- Please rename "Conflict of Interest" to "Disclosure Statement & Competing Interests". We updated our journal's competing interests policy in January 2022 and request authors to consider both actual and perceived competing interests. Please review the policy https://www.embopress.org/competing-interests and update your competing interests if necessary.
We have made changes as suggested.
- Author contributions: Please remove it from the manuscript and specify author contributions in our submission system. CRediT has replaced the traditional author contributions section because it offers a systematic machine-readable author contributions format that allows for more effective research assessment. You are encouraged to use the free text boxes beneath each contributing author's name to add specific details on the author's contribution. More information is available in our guide to authors: https://www.embopress.org/page/journal/17574684/authorguide#authorshipguidelines
We have made changes as suggested.

3) Appendix: Please rename "Supporting information" to "Appendix" and remove all EV Figures and their legends and leave only the 2 tables. Tables should be renamed to Appendix Table S1 and S2 and the table of content amended accordingly. EV Figure legends should be moved to the main manuscript file and placed after the main figure legends.
We have made changes as suggested.

4) Funding: Please make sure the information about funding are complete in both our submission systema and in the manuscript. Currently information about the Regional Centre for Biotechnology (RCB), Indian Council of Medical Research (ICMR), the University Grants

Commission (UGC), Department of Science and Technology INSPIRE junior research fellowship, ICMR junior research fellowship and the Science and Engineering Research Board are missing in our submission system.
We have added the funding information that was missing in the system submission.

5) The Paper Explained: Please provide "The Paper Explained" and add it to the main manuscript text. Please refer to any of our published primary research articles for an example. Check "Author Guidelines" for more information. https://www.embopress.org/page/journal/17574684/authorguide#researcharticleguide
"The Paper Explained" has been added to the main manuscript text.

6) Synopsis: Every published paper now includes a 'Synopsis' to further enhance discoverability. Synopses are displayed on the journal webpage and are freely accessible to all readers. They include separate synopsis image and synopsis text.
- Synopsis image: Please provide a striking image or visual abstract as a high-resolution jpeg file 550 px-wide x (250-400)-px high to illustrate your article.
- Synopsis text: Please provide a short standfirst (maximum of 300 characters, including space) as well as 2-5 one sentence bullet points that summarise the paper as a .doc file. Please write the bullet points to summarise the key NEW findings. They should be designed to be complementary to the abstract - i.e. not repeat the same text. We encourage inclusion of key acronyms and quantitative information (maximum of 30 words / bullet point). Please use the passive voice.
- Please check your synopsis text and image before submission with your revised manuscript. Please be aware that in the proof stage minor corrections only are allowed (e.g., typos).
Synopsis text and image have been included.

7) For more information: This space should be used to list relevant web links for further consultation by our readers. Could you identify some relevant ones and provide such information as well? Some examples are patient associations, relevant databases, OMIM/proteins/genes links, author's websites, etc...
We have added the OMIM link for SCTS under this section.

8) As part of the EMBO Publications transparent editorial process initiative (see our Editorial at http://embomolmed.embopress.org/content/2/9/329), EMBO Molecular Medicine will publish online a Review Process File (RPF) to accompany accepted manuscripts. This file will be published in conjunction with your paper and will include the anonymous referee reports, your point-by-point response and all pertinent correspondence relating to the manuscript. Let us know whether you agree with the publication of the RPF and as here, if you want to remove or not any figures from it prior to publication. Please note that the Authors checklist will be published at the end of the RPF.
We agree for the complete RPF and authors checklist to be published with this manuscript.

9) Please provide a point-by-point letter INCLUDING my comments as well as the reviewer's reports and your detailed responses (as Word file).
Included.

I look forward to reading a new revised version of your manuscript as soon as possible.

Yours sincerely,

Zeljko Durdevic
Editor
EMBO Molecular Medicine

***** Reviewer's comments *****
Referee #2 (Comments on Novelty/Model System for Author):

This is an exciting manuscript linking early contractility to YAP/hippo signaling. The effects of early loss of MYH3 are striking on these pathways and the rescue of the phenotype with YAP inhibitors may lead to novel human treatments.

Referee #2 (Remarks for Author):

The authors have carefully responded to all prior reviews with additional experiments that provide additional support for their conclusions, even that the hippo pathway is also altered by changes in other early expressed myosin genes. My only minor suggestion would be to add 1-3 sentences that emphasize the relationship between MYH3 and FLNB. Knockout of MYH3 caused marked reduction in FLNB and both alterations of both genes cause SCTS, which were noted by the reviewers. Is there existing data about loss of FLNB that showing similar effects on downstream targets?
All prior questions have been adequately addressed in the manuscript and in the response to reviewers.
We thank the Reviewer for reviewing our manuscript and are grateful for the comments and suggestions, which have been on point and has helped us improve the manuscript considerably. We have added a sentence in the discussion (line numbers 485-487) and cited two papers to address the minor comment by the Reviewer.

Referee #3 (Comments on Novelty/Model System for Author):

The manuscript is appropriate to evaluate the roles of Myosin Heavy Chain (specifically Myh3) which has an impact on the human spondylocarpotarsal synostosis (SCTS) disorder. The ability to dissect out individual isoforms and adequately model their function is appropriate in a knockout mouse model. No concerns are noted with the model choice.

Referee #3 (Remarks for Author):

The authors have done an impressive job in response to my and the other reviewer comments. I appreciate the additional work elucidating additional satellite cell expression and acknowledge that some of their previous work focused on satellite cells in the Myh3 knockout. Additional functional testing of the Myh3 knockout mice is supportive of the model and the authors' hypotheses. Additional supportive data on TEAD localization is also well conducted. I have no additional concerns and believe that the manuscript is suitable for publication in EMBO Molecular Medicine.
We are grateful to the Reviewer for reviewing our manuscript and coming up with excellent suggestions and comments. This has indeed been of great help for us to improve the manuscript.

17th Jul 2023

Dear Dr. Mathew,

We are pleased to inform you that your manuscript is accepted for publication and is now being sent to our publisher to be included in the next available issue of EMBO Molecular Medicine.

Please read below for additional IMPORTANT information regarding your article, its publication and the production process.

Congratulations on your interesting work,

Zeljko Durdevic

Zeljko Durdevic
Editor
EMBO Molecular Medicine

Follow us on Twitter @EmboMolMed
Sign up for eTOCs at embopress.org/alertsfeeds

*** *** *** IMPORTANT INFORMATION *** *** ***

SPEED OF PUBLICATION
The journal aims for rapid publication of papers, using using the advance online publication "Early View" to expedite the process: A properly copy-edited and formatted version will be published as "Early View" after the proofs have been corrected. Please help the Editors and publisher avoid delays by providing e-mail address(es), telephone and fax numbers at which author(s) can be contacted.

Should you be planning a Press Release on your article, please get in contact with embomolmed@wiley.com as early as possible, in order to coordinate publication and release dates.

LICENSE AND PAYMENT:

All articles published in EMBO Molecular Medicine are fully open access: immediately and freely available to read, download and share.

EMBO Molecular Medicine charges an article processing charge (APC) to cover the publication costs. You, as the corresponding author for this manuscript, should have already received a quote with the article processing fee separately. Please let us know in case this quote has not been received.

Once your article is at Wiley for editorial production you will receive an email from Wiley's Author Services system, which will ask you to log in and will present you with the publication license form for completion. Within the same system the publication fee can be paid by credit card, an invoice, pro forma invoice or purchase order can be requested.

Payment of the publication charge and the signed Open Access Agreement form must be received before the article can be published online.

PROOFS

You will receive the proofs by e-mail approximately 2 weeks after all relevant files have been sent o our Production Office. Please return them within 48 hours and if there should be any problems, please contact the production office at embopressproduction@wiley.com.

Please inform us if there is likely to be any difficulty in reaching you at the above address at that time. Failure to meet our

deadlines may result in a delay of publication.

All further communications concerning your paper proofs should quote reference number EMM-2022-17187-V3 and be directed to the production office at embopressproduction@wiley.com.

Thank you,

Zeljko Durdevic
Editor
EMBO Molecular Medicine

EMBO Press Author Checklist

Corresponding Author Name: Sam J. Mathew
Journal Submitted to: EMBO Molecular Medicine
Manuscript Number: EMM-2022-17187

USEFUL LINKS FOR COMPLETING THIS FORM
The EMBO Journal - Author Guidelines
EMBO Reports - Author Guidelines
Molecular Systems Biology - Author Guidelines
EMBO Molecular Medicine - Author Guidelines

Reporting Checklist for Life Science Articles (updated January
This checklist is adapted from Materials Design Analysis Reporting (MDAR) Checklist for Authors. MDAR establishes a minimum set of requirements in transparent reporting in the life sciences (see Statement of Task: 10.31222/osf.io/9sm4x). Please follow the journal's guidelines in preparing your manuscript.
Please note that a copy of this checklist will be published alongside your article.

Abridged guidelines for figures
1. Data
The data shown in figures should satisfy the following conditions:
- the data were obtained and processed according to the field’s best practice and are presented to reflect the results of the experiments in an accurate and unbiased manner.
- ideally, figure panels should include only measurements that are directly comparable to each other and obtained with the same assay.
- plots include clearly labeled error bars for independent experiments and sample sizes. Unless justified, error bars should not be shown for technical
- if n<5, the individual data points from each experiment should be plotted. Any statistical test employed should be justified.
- Source Data should be included to report the data underlying figures according to the guidelines set out in the authorship guidelines on Data

2. Captions
Each figure caption should contain the following information, for each panel where they are relevant:
- a specification of the experimental system investigated (eg cell line, species name).
- the assay(s) and method(s) used to carry out the reported observations and measurements.
- an explicit mention of the biological and chemical entity(ies) that are being measured.
- an explicit mention of the biological and chemical entity(ies) that are altered/varied/perturbed in a controlled manner.
- the exact sample size (n) for each experimental group/condition, given as a number, not a range;
- a description of the sample collection allowing the reader to understand whether the samples represent technical or biological replicates (including how many animals, litters, cultures, etc.).
- a statement of how many times the experiment shown was independently replicated in the laboratory.
- definitions of statistical methods and measures:
  - common tests, such as t-test (please specify whether paired vs. unpaired), simple χ2 tests, Wilcoxon and Mann-Whitney tests, can be unambiguously identified by name only, but more complex techniques should be described in the methods section;
  - are tests one-sided or two-sided?
  - are there adjustments for multiple comparisons?
  - exact statistical test results, e.g., P values = x but not P values < x;
  - definition of ‘center values’ as median or average;
  - definition of error bars as s.d. or s.e.m.

Please complete ALL of the questions below.
Select "Not Applicable" only when the requested information is not relevant for your study.

Materials

Newly Created Materials	Information included in the manuscript?	In which section is the information available? (Reagents and Tools Table, Materials and Methods, Figures, Data Availability Section)
New materials and reagents need to be available; do any restrictions apply?	Not Applicable	

Antibodies	Information included in the manuscript?	In which section is the information available? (Reagents and Tools Table, Materials and Methods, Figures, Data Availability Section)
For **antibodies** provide the following information:<br>- Commercial antibodies: RRID (if possible) or supplier name, catalogue number and or/clone number<br>- Non-commercial: RRID or citation	Yes	Appendix: Table S1

DNA and RNA sequences	Information included in the manuscript?	In which section is the information available? (Reagents and Tools Table, Materials and Methods, Figures, Data Availability Section)
**Short novel DNA or RNA including primers, probes**: provide the sequences.	Yes	Appendix: Table S2

Cell materials	Information included in the manuscript?	In which section is the information available? (Reagents and Tools Table, Materials and Methods, Figures, Data Availability Section)
**Cell lines:** Provide species information, strain. Provide accession number in repository **OR** supplier name, catalog number, clone number, and/**OR** RRID.	Yes	Materials and Methods
**Primary cultures:** Provide species, strain, sex of origin, genetic modification status.	Yes	Materials and Methods
Report if the cell lines were recently **authenticated** (e.g., by STR profiling) and tested for mycoplasma contamination.	Not Applicable	

Experimental animals	Information included in the manuscript?	In which section is the information available? (Reagents and Tools Table, Materials and Methods, Figures, Data Availability Section)
**Laboratory animals or Model organisms:** Provide species, strain, sex, age, genetic modification status. Provide accession number in repository **OR** supplier name, catalog number, clone number, **OR** RRID.	Yes	Materials and Methods
**Animal observed in or captured from the field:** Provide species, sex, and age where possible.	Not Applicable	
Please detail **housing and husbandry conditions.**	Yes	Materials and Methods

Plants and microbes	Information included in the manuscript?	In which section is the information available? (Reagents and Tools Table, Materials and Methods, Figures, Data Availability Section)
**Plants:** provide species and strain, ecotype and cultivar where relevant, unique accession number if available, and source (including location for collected wild specimens).	Not Applicable	
**Microbes:** provide species and strain, unique accession number if available, and source.	Not Applicable	

Human research participants	Information included in the manuscript?	In which section is the information available? (Reagents and Tools Table, Materials and Methods, Figures, Data Availability Section)
If collected and within the bounds of privacy constraints report on age, sex and gender or ethnicity for all study participants.	Not Applicable	

Core facilities	Information included in the manuscript?	In which section is the information available? (Reagents and Tools Table, Materials and Methods, Figures, Data Availability Section)
If your work benefited from core facilities, was their service mentioned in the acknowledgments section?	Yes	Acknowledgements

Design

Study protocol	Information included in the manuscript?	In which section is the information available? (Reagents and Tools Table, Materials and Methods, Figures, Data Availability Section)
If study protocol has been **pre-registered, provide DOI in the manuscript**. For clinical trials, provide the trial registration number **OR** cite DOI.	Not Applicable	
Report the **clinical trial registration number** (at ClinicalTrials.gov or equivalent), where applicable.	Not Applicable	

Laboratory protocol	Information included in the manuscript?	In which section is the information available? (Reagents and Tools Table, Materials and Methods, Figures, Data Availability Section)
Provide DOI OR other citation details if **external detailed step-by-step protocols** are available.	Yes	Materials and Methods

Experimental study design and statistics	Information included in the manuscript?	In which section is the information available? (Reagents and Tools Table, Materials and Methods, Figures, Data Availability Section)
Include a statement about **sample size** estimate even if no statistical methods were used.	Yes	Figure Legends
Were any steps taken to minimize the effects of subjective bias when allocating animals/samples to treatment (e.g. **randomization procedure**)? If yes, have they been described?	Yes	Material and Methods
Include a statement about **blinding** even if no blinding was done.	Yes	Material and Methods
Describe **inclusion/exclusion criteria** if samples or animals were excluded from the analysis. Were the criteria pre-established?<br><br>If sample or data points were omitted from analysis, report if this was due to attrition or intentional exclusion and provide justification.	Not Applicable	
For every figure, are **statistical tests** justified as appropriate? Do the data meet the assumptions of the tests (e.g., normal distribution)? Describe any methods used to assess it. Is there an estimate of variation within each group of data? Is the variance similar between the groups that are being statistically compared?	Yes	Figure Legends

Sample definition and in-laboratory replication	Information included in the manuscript?	In which section is the information available? (Reagents and Tools Table, Materials and Methods, Figures, Data Availability Section)
In the figure legends: state number of times the experiment was **replicated** in laboratory.	Yes	Figure Legends
In the figure legends: define whether data describe **technical or biological replicates**.	Yes	Figure Legends, Material and Methods

Ethics

Ethics	Information included in the manuscript?	In which section is the information available? (Reagents and Tools Table, Materials and Methods, Figures, Data Availability Section)
Studies involving **human participants**: State details of **authority granting ethics approval** (IRB or equivalent committee(s), provide reference number for approval.	Not Applicable	
Studies involving **human participants**: Include a statement confirming that **informed consent** was obtained from all subjects and that the experiments conformed to the principles set out in the WMA Declaration of Helsinki and the Department of Health and Human Services Belmont Report.	Not Applicable	
Studies involving **human participants:** For publication of **patient photos**, include a statement confirming that consent to publish was obtained.	Not Applicable	
Studies involving experimental **animals**: State details of **authority granting ethics approva**l (IRB or equivalent committee(s), provide reference number for approval. Include a statement of compliance with ethical regulations.	Yes	Material and Methods
Studies involving **specimen and field samples:** State if relevant **permits** obtained, provide details of authority approving study; if none were required, explain why.	Not Applicable	

Dual Use Research of Concern (DURC)	Information included in the manuscript?	In which section is the information available? (Reagents and Tools Table, Materials and Methods, Figures, Data Availability Section)
Could your study fall under dual use research restrictions? Please check biosecurity documents and list of **select agents and toxins** (CDC): https://www.selectagents.gov/sat/list.htm	Not Applicable	
If you used a select agent, is the security level of the lab appropriate and reported in the manuscript?	Not Applicable	
If a study is subject to dual use research of concern regulations, is the name of the **authority granting approval and reference number** for the regulatory approval provided in the manuscript?	Not Applicable	

Reporting

The MDAR framework recommends adoption of discipline-specific guidelines, established and endorsed through community initiatives. Journals have their own policy about requiring specific guidelines and recommendations to complement MDAR.

Adherence to community standards	Information included in the manuscript?	In which section is the information available? (Reagents and Tools Table, Materials and Methods, Figures, Data Availability Section)
State if relevant guidelines or checklists (e.g., **ICMJE, MIBBI, ARRIVE, PRISMA**) have been followed or provided.	Not Applicable	
For **tumor marker prognostic studies,** we recommend that you follow the **REMARK** reporting guidelines (see link list at top right). See author guidelines, under 'Reporting Guidelines'. Please confirm you have followed these guidelines.	Not Applicable	
For **phase II and III randomized controlled trials**, please refer to the **CONSORT** flow diagram (see link list at top right) and submit the CONSORT checklist (see link list at top right) with your submission. See author guidelines, under 'Reporting Guidelines'. Please confirm you have submitted this list.	Not Applicable	

Data Availability

Data availability	Information included in the manuscript?	In which section is the information available? (Reagents and Tools Table, Materials and Methods, Figures, Data Availability Section)
Have **primary datasets** been deposited according to the journal's guidelines (see 'Data Deposition' section) and the respective accession numbers provided in the Data Availability Section?	Not Applicable	
Were **human clinical and genomic datasets** deposited in a public access-controlled repository in accordance to ethical obligations to the patients and to the applicable consent agreement?	Not Applicable	
Are **computational models** that are central and integral to a study available without restrictions in a machine-readable form? Were the relevant accession numbers or links provided?	Not Applicable	
If publicly available data were reused, provide the respective **data citations in the reference list**.	Not Applicable	
